# Supplementary material for: LoCHAid: An ultra-low-cost hearing aid for age-related hearing loss
Source: PLoS One. 2020 Sep 23;15(9):e0238922. doi: 10.1371/journal.pone.0238922 (PMC7510997; doi:10.1371/journal.pone.0238922)
Supplement: S1 File — (PDF) [file pone.0238922.s005.pdf]

Supplementary Information - The LoCHAid: An low-cost, open-source hearing aid for Age Related Hearing Loss

Soham Sinha,[1] Urvaksh D. Irani,[2] Vinaya Manchaiah,[3] and M. Saad Bhamla[1,*]

[1]*School of Chemical & Biomolecular Engineering, Georgia Institute of Technology, 311 Ferst Drive NW, Altanta, GA 30332, USA*

[2]*School of Mechanical Engineering, Georgia Institute of Technology, 311 Ferst Drive NW, Atlanta, GA 30332, USA*

[3]*Department of Speech and Hearing Sciences, Lamar University, 4400 S M L King Jr Pkwy, Beaumont, TX 77705, USA*

(Dated: July 22, 2020)

CONTENTS

I. Data Availability 2

II. Supplementary Movies 2
- A. S1: Construction of the LoCHAid 2
- B. S2: Preparing Earphones for Testing 2
- C. S3: Water Depth Testing of Device 2
- D. S4: Drop Testing Device 2

III. Schematic 2

IV. PCB Layout 2

V. ARHL Profiles 2

VI. Discussion on Equivalent Input Noise 3

VII. Supplementary Master Graphs of Audiological Profiles and Targets (dB SPL) 3
- A. ISTS 55 dB SPL Master Graph 3
- B. ISTS 80 dB SPL Master Graph 3
- C. G.R.A.S KEMAR Left Ear ISTS 65 dB SPL Master Graph 3
- D. G.R.A.S KEMAR Right Ear ISTS 65 dB SPL Master Graph 3
- E. G.R.A.S KEMAR Right Ear Graph with ISTS 65 Speechmap Response (dB SPL) 3
- F. G.R.A.S KEMAR Left Ear Graph with ISTS 65 Speechmap Response (dB SPL) 3

VIII. Quantification of Fits via Strict and Loose Criteria 3

IX. Supplementary Individual Graphs of Audiological Profiles and Targets (dB SPL) 3
- A. Male Age 60-69 Left Ear 3
  1. ISTS 55 dB SPL 3
  2. ISTS 65 dB SPL 4
  3. ISTS 80 dB SPL 4
  4. KEMAR Right Ear 65 dB SPL 4
  5. KEMAR Left Ear 65 dB SPL 4
- B. Male Age 60-69 Right Ear 4
  1. ISTS 55 dB SPL 4
  2. ISTS 65 dB SPL 4
  3. ISTS 80 dB SPL 4
  4. KEMAR Right Ear 65 dB SPL 4
  5. KEMAR Left Ear 65 dB SPL 4
- C. Male Age 70-79 Left Ear 4
  1. ISTS 55 dB SPL 4
  2. ISTS 65 dB SPL 4
  3. ISTS 80 dB SPL 4
  4. KEMAR Right Ear 65 dB SPL 4
  5. KEMAR Left Ear 65 dB SPL 4
- D. Male Age 70-79 Right Ear 4
  1. ISTS 55 dB SPL 4
  2. ISTS 65 dB SPL 4
  3. ISTS 80 dB SPL 4
  4. KEMAR Right Ear 65 dB SPL 4
  5. KEMAR Left Ear 65 dB SPL 4
- E. Female Age 60-69 Left Ear 4
  1. ISTS 55 dB SPL 4
  2. ISTS 65 dB SPL 4
  3. ISTS 80 dB SPL 4
  4. KEMAR Right Ear 65 dB SPL 5
  5. KEMAR Left Ear 65 dB SPL 5
- F. Female Age 60-69 Right Ear 5
  1. ISTS 55 dB SPL 5
  2. ISTS 65 dB SPL 5
  3. ISTS 80 dB SPL 5
  4. KEMAR Right Ear 65 dB SPL 5
  5. KEMAR Left Ear 65 dB SPL 5
- G. Female Age 70-79 Left Ear 5
  1. ISTS 55 dB SPL 5
  2. ISTS 65 dB SPL 5
  3. ISTS 80 dB SPL 5
  4. KEMAR Right Ear 65 dB SPL 5
  5. KEMAR Left Ear 65 dB SPL 5
- H. Female Age 70-79 Right Ear 5
  1. ISTS 55 dB SPL 5
  2. ISTS 65 dB SPL 5
  3. ISTS 80 dB SPL 5
  4. KEMAR Right Ear 65 dB SPL 5
  5. KEMAR Left Ear 65 dB SPL 5
- I. Hearing Profile X 5
  1. ISTS 55 dB SPL 5
  2. ISTS 65 dB SPL 5
  3. ISTS 80 dB SPL 5

* Please address correspondence to: M.S.B (saadb@chbe.gatech.edu)

4. KEMAR Right Ear 65 dB SPL 5
5. KEMAR Left Ear 65 dB SPL 5
J. Hearing Profile Y 6
1. ISTS 55 dB SPL 6
2. ISTS 65 dB SPL 6
3. ISTS 80 dB SPL 6
4. KEMAR Right Ear 65 dB SPL 6
5. KEMAR Left Ear 65 dB SPL 6
K. Hearing Profile Y 6
1. ISTS 55 dB SPL 6
2. ISTS 65 dB SPL 6
3. ISTS 80 dB SPL 6
4. KEMAR Right Ear 65 dB SPL 6
5. KEMAR Left Ear 65 dB SPL 6
L. Hearing Profile Z 6
1. ISTS 55 dB SPL 6
2. ISTS 65 dB SPL 6
3. ISTS 80 dB SPL 6
4. KEMAR Right Ear 65 dB SPL 6
5. KEMAR Left Ear 65 dB SPL 6

References 7

I. DATA AVAILABILITY

The authors maintain that all data is available and can be found in this Github link.

II. SUPPLEMENTARY MOVIES

A. S1: Construction of the LoCHAid

Video outlining construction of the LoCHAid with Lithium Ion Coin Cell Battery. Video speed has been increased to 15x; however, the average time of construction is 25 minutes. Figure S6 shows the schematic of the hearing aid.

B. S2: Preparing Earphones for Testing

Video outlining how to properly set up device earbuds for audiological testing in AudioScan Verifit with 0.2-cc coupler.

C. S3: Water Depth Testing of Device

Video showing device after being submerged in 6cm of water, and shows that it still is in working condition. A still photo is shown in Figure S5b.

D. S4: Drop Testing Device

Video showing device after repeated drop tested (n=10) from a height of 5 feet, and showing that the device is in working condition. A still photo is shown in Figure S5a.

III. SCHEMATIC

The schematic of the LoCHAid is given in Figure S6. The .brd files can be found in the github folder associated with this manuscript, which can be uploaded to fab-house website such as OSHPARK (www.oshpark.com) for PCB fabrication.

IV. PCB LAYOUT

The layout of the LoCHAid in the smallest configuration is given in Figure S7. The manufactured device is given in Figure S8. The .brd files can be found in the github folder associated with this manuscript, and this can be uploaded to fab-house wesbiste as Macrofab (www.macrofab.com) for both PCB and parts assembly.

V. ARHL PROFILES

Figure S1 shows the audiograms used for analysis and comparison. They contain both male and female aged 60-69, 70-79 for both left and right ears. The male audiograms are steeper and have sharper fall offs, as compared to female audiograms. The data comes from two studies. Cruikshanks, et al from 1993-1995 [1] conducted hearing tests of both ears of people living in Beaver Dam, Wisconsion, and Ciletti and Flamme [2] who conducted hearing loss analysis cross country in USA. Cruikshanks, et al from 1993-1995 conducted hearing tests of both ears of people living in Beaver Dam, Wisconsion, and pulls data from 566 females from the age of 60-69, 534 females from ages 70-79, 489 males from ages 60-69, and 355 males from the ages of 70-79 and detail left and right ear hearing losses. Hearing Profiles X,Y, and Z are gender neutral audiograms displaying ARHL characteristics extracted from a study done by Ciletti and Flamme, which was conducted over 1999-2005, based on the National Health and Nutrition Examination Survey (NHANES) which was a cross-country sample study of common audiogram trends, based on 2819 women, and 2525 men ages 20-69. These results are in agreement with other work [3, 4].

VI. DISCUSSION ON EQUIVALENT INPUT NOISE

EIN at 40 dB SPL can be problematic as many people with mild hearing loss at a threshold of 0-20 dB HL are likely to hear this sound and this internal noise can interfere with their speech understanding. However, for the majority of the hearing loss profiles investigated the average threshold is 40 dB HL [1, 2]. A study conducted by Macae and Dillon in 1996 specified a relaxation criteria up to 46.3 dB SPL for a gain of 15 dB at a threshold of 20-50 dB HL. Based on this, we think that the EIN will interfere very little for patients exhibiting mild to moderate ARHL [5].

We were able to track the source of the EIN In the circuit to the MAX9814 module itself at 30 nV/$\sqrt{Hz}$ [6], and to the best of our knowledge, no filter was able to drop the EIN to below 40 dB SPL. We undertook experimentation with different Maxim models such as MAX4466, Silicon MEMS microphone SPW2430 (www.adafruit.com). We undertook different model configurations with using op-amp TLV2462 in replacing the MAX98306, and found out that it did not only did it not reduce the EIN, but it also impacted the frequency response. Overall, we tested 12 different configurations with different components, both passive and active filter circuits, to reduce EIN, but we were not successful in bringing down the EIN at the cost boundary of 1 USD. Hence, a decision was made not to address this feature at this stage as the electroacoustic analysis showed that the LoCHAid has high frequency gain that is necessary for mild-moderate ARHL.

VII. SUPPLEMENTARY MASTER GRAPHS OF AUDIOLOGICAL PROFILES AND TARGETS (DB SPL)

The following figures detail all audiological profiles and their respective Targets (dB SPL) for each differing input ISTS 55 dB SPL,65 dB SPL, 80 dB SPL, and G.R.A.S KEMAR Left and Right Ears at ISTS 65 dB SPL

A. ISTS 55 dB SPL Master Graph

The graph is shown in figure number S9.

B. ISTS 80 dB SPL Master Graph

The graph is shown in figure number S10

C. G.R.A.S KEMAR Left Ear ISTS 65 dB SPL Master Graph

The graph is shown in figure number S12.

D. G.R.A.S KEMAR Right Ear ISTS 65 dB SPL Master Graph

The graph is shown in figure number S11.

E. G.R.A.S KEMAR Right Ear Graph with ISTS 65 Speechmap Response (dB SPL)

The graph is shown in figure number S13.

F. G.R.A.S KEMAR Left Ear Graph with ISTS 65 Speechmap Response (dB SPL)

The graph is shown in figure number S14.

VIII. QUANTIFICATION OF FITS VIA STRICT AND LOOSE CRITERIA

We present a visual graph indicating how well LoCHAid fits the profiles in Figure S2. The blue squares indicate that it met, and red squares indicate that it did not meet. It is organised by profiles in the horizontal axis, and the target frequencies on the vertical axis. Each profile has two sections denoting strict and loose criteria. The profiles are ordered from most number of blue squares to least number of blue squares. This is when the device is at full on gain, so to meet individual profiles, the device volume has to be lowered by 5-10 dB SPL depending on the profile being considered. Overall, this shows how well the device fits the targets. We also showed how the profiles met in G.R.A.S KEMAR, where we show that without volume considerations, the profiles offer very good fit in Figure S3.

IX. SUPPLEMENTARY INDIVIDUAL GRAPHS OF AUDIOLOGICAL PROFILES AND TARGETS (DB SPL)

The following figures detail individual audiological profile, and their respective Targets (dB SPL) for each differing input (ISTS 55,65 dB SPL, and 80 dB SPL, and G.R.A.S KEMAR Left and Right Ears at 65 dB SPL). Meets Strict Criteria refers to the Response (dB SPL) being within 5 dB SPL of the target. Meets Loose Criteria Refers to the Response (dB SPL) being within 10 dB SPL of the target.

A. Male Age 60-69 Left Ear

1. *ISTS 55 dB SPL*

Figure S15. Table I.

2. ISTS 65 dB SPL

Figure S16. Table II.

3. ISTS 80 dB SPL

Figure S17. Table III

4. KEMAR Right Ear 65 dB SPL

. Figure S18. Table V.

5. KEMAR Left Ear 65 dB SPL

Figure S19. Table IV.

B. Male Age 60-69 Right Ear

1. ISTS 55 dB SPL

Figure S20. Table VI

2. ISTS 65 dB SPL

Figure S21. Table VII.

3. ISTS 80 dB SPL

Figure S22. Table VIII.

4. KEMAR Right Ear 65 dB SPL

Figure S23. Table X.

5. KEMAR Left Ear 65 dB SPL

Figure S24. Table IX.

C. Male Age 70-79 Left Ear

1. ISTS 55 dB SPL

Figure S35. Table XXI.

2. ISTS 65 dB SPL

Figure S36. Table XXII.

3. ISTS 80 dB SPL

Figure S37. Table XXIII.

4. KEMAR Right Ear 65 dB SPL

Figure S38. Table XXIV.

5. KEMAR Left Ear 65 dB SPL

Figure S39. Table XXV.

D. Male Age 70-79 Right Ear

1. ISTS 55 dB SPL

Figure S30. Table XVI.

2. ISTS 65 dB SPL

Figure S31. Table XVII.

3. ISTS 80 dB SPL

Figure S32. Table XVIII.

4. KEMAR Right Ear 65 dB SPL

Figure S33. Table XIX.

5. KEMAR Left Ear 65 dB SPL

Figure S34. Table XX.

E. Female Age 60-69 Left Ear

1. ISTS 55 dB SPL

Figure S40. Table XXVI.

2. ISTS 65 dB SPL

Figure S41. Table XXVII.

3. ISTS 80 dB SPL

Figure S42. Table XXVIII.

4. *KEMAR Right Ear 65 dB SPL*

Figure S43. Table XXIX.

5. *KEMAR Left Ear 65 dB SPL*

Figure S44. Table XXX.

F. Female Age 60-69 Right Ear

1. *ISTS 55 dB SPL*

Figure S45. Table XXXI.

2. *ISTS 65 dB SPL*

Figure S46. Table XXXII.

3. *ISTS 80 dB SPL*

Figure S47. Table XXXIII.

4. *KEMAR Right Ear 65 dB SPL*

Figure S48. Table XXXIV.

5. *KEMAR Left Ear 65 dB SPL*

Figure S49. Table XXXV.

G. Female Age 70-79 Left Ear

1. *ISTS 55 dB SPL*

Figure S60. Table XXXVI.

2. *ISTS 65 dB SPL*

Figure S61. Table XXXVII.

3. *ISTS 80 dB SPL*

Figure S62. Table XXXVIII.

4. *KEMAR Right Ear 65 dB SPL*

Figure S63. Table XXXIX.

5. *KEMAR Left Ear 65 dB SPL*

Figure S64. Table XL.

H. Female Age 70-79 Right Ear

1. *ISTS 55 dB SPL*

Figure S55. Table XLI.

2. *ISTS 65 dB SPL*

Figure S56. Table XLII.

3. *ISTS 80 dB SPL*

Figure S57. Table XLIII.

4. *KEMAR Right Ear 65 dB SPL*

Figure S58. Table XLIV.

5. *KEMAR Left Ear 65 dB SPL*

Figure S59. Table XLV.

I. Hearing Profile X

1. *ISTS 55 dB SPL*

Figure S65. Table XLVI.

2. *ISTS 65 dB SPL*

Figure S66. Table XLVII.

3. *ISTS 80 dB SPL*

Figure S67. Table XLVIII.

4. *KEMAR Right Ear 65 dB SPL*

Figure S68. Table XLIX.

5. *KEMAR Left Ear 65 dB SPL*

Figure S69. Table L.

J. Hearing Profile Y

1. ISTS 55 dB SPL

Figure S70. Table LI.

2. ISTS 65 dB SPL

Figure S71. Table LII.

3. ISTS 80 dB SPL

Figure S72. Table LIII.

4. KEMAR Right Ear 65 dB SPL

Figure S73. Table LIV.

5. KEMAR Left Ear 65 dB SPL

Figure S74. Table LV.

K. Hearing Profile Y

1. ISTS 55 dB SPL

Figure S70. Table LI.

2. ISTS 65 dB SPL

Figure S71. Table LII.

3. ISTS 80 dB SPL

Figure S72. Table LIII.

4. KEMAR Right Ear 65 dB SPL

Figure S73. Table LIV.

5. KEMAR Left Ear 65 dB SPL

Figure S74. Table LV.

L. Hearing Profile Z

1. ISTS 55 dB SPL

Figure S75. Table LVI.

2. ISTS 65 dB SPL

Figure S76. Table LVII.

3. ISTS 80 dB SPL

Figure S77. Table LVIII.

4. KEMAR Right Ear 65 dB SPL

Figure S78. Table LIX.

5. KEMAR Left Ear 65 dB SPL

Figure S79. Table LX

[1] Karen J. Cruickshanks, Terry L. Wiley, Theodore S. Tweed, Barbara E.K. Klein, Ronald Klein, Julie A. Mares-Perlman, and David M. Nondahl, "Prevalence of hearing loss in older adults in Beaver dam, Wisconsin. The epidemiology of hearing loss study," American Journal of Epidemiology **148**, 879–886 (1998).

[2] Lindsay Ciletti and Gregory A. Flamme, "Prevalence of hearing impairment by gender and audiometric configuration: Results from the National Health and Nutrition Examination Survey (1999-2004) and the Keokuk County Rural Health Study (1994-1998)," Journal of the American Academy of Audiology **19**, 672–685 (2008).

[3] Kelly Demeester, Astrid Van Wieringen, Jan Jaap Hendrickx, Vedat Topsakal, Erik Fransen, Lut Van Laer, Guy Van Camp, and Paul Van De Heyning, "Audiometric shape and presbycusis," International Journal of Audiology **48**, 222–232 (2009).

[4] Olusola A. Sogebi, O. O. Olusoga-Peters, and O. Oluwapelumi, "Clinical and audiometric features of Presbycusis in Nigerians," African Health Sciences **13**, 886–892 (2013).

[5] John H. Macrae and Harvey Dillon, "Gain, frequency response, and maximum output requirements for hearing aids," Journal of Rehabilitation Research and Development **33**, 363–376 (1996).

[6] Maxim Integrated Products, "Microphone Amplifier with AGC and Low-Noise Microphone Bias MAX9814 Microphone Amplifier with AGC and Low-Noise Microphone Bias: Data Sheet," Maxim Integrated Products (2016).

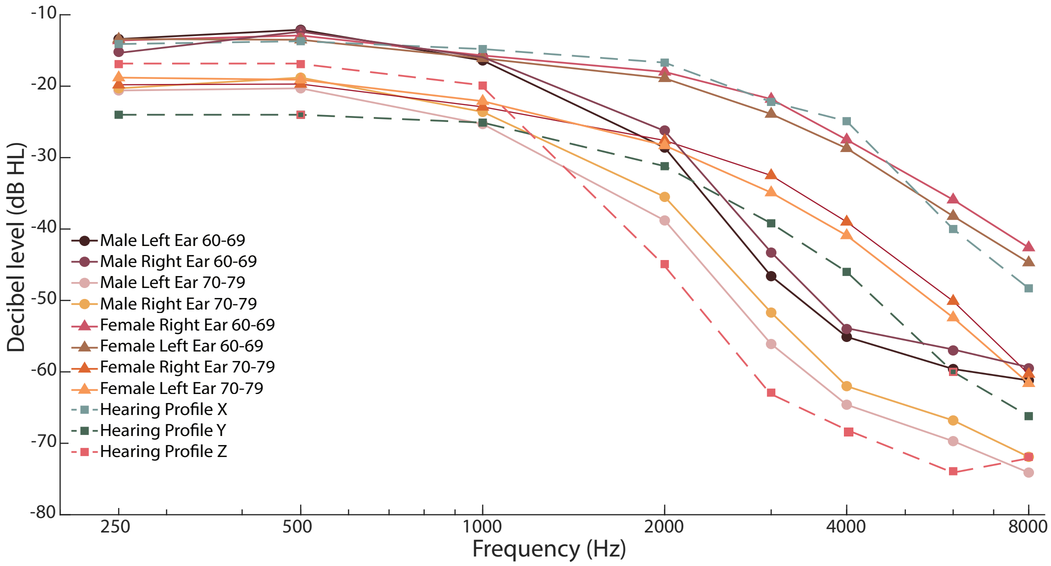

FIG. S1. Shows the 12 profiles used in the study detailing Males and Females both left and right ears from ages 60-79. The figure uses data extracted from two studies [2], [1]. Cruikshanks, et al from 1993-1995 [1] conducted hearing tests of both ears of people living in Beaver Dam, Wisconsion, and Ciletti and Flamme [2] who conducted hearing loss analysis cross country in USA. Cruikshanks, et al from 1993-1995 conducted hearing tests of both ears of people living in Beaver Dam, Wisconsion, and pulls data from 566 females from the age of 60-69, 534 females from ages 70-79, 489 males from ages 60-69, and 355 males from the ages of 70-79 and detail left and right ear hearing losses. Hearing Profiles X,Y, and Z are gender neutral audiograms of increasing severity displaying ARHL characteristics extracted from a study done by Ciletti and Flamme, which was conducted over 1999-2005, based on the National Health and Nutrition Examination Survey (NHANES) which was a cross-country sample study of common audiogram trends, based on 2819 women, and 2525 men ages 20-69. Hearing Profile X (mild) is exhibited by 204 men, 282 women; Hearing Profile Y (moderate) is exhibited by 123 women, and 145 men; Hearing Profile Z (Severe) is exhibited by 174 men, 19 women.

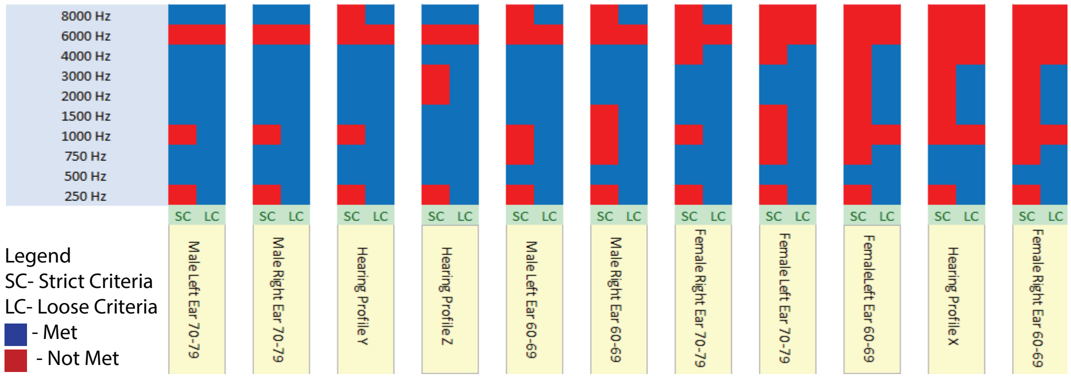

FIG. S2. Shows the quantification of fit in a visual matter for Speechmap. It is organised by profiles in the horizontal axis, and the target frequencies on the vertical axis. Each profile has two sections denoting strict and loose criteria. The profiles are ordered from most number of blue squares to least number of blue squares. This is when the device is at full on gain, so to meet individual profiles, the device volume has to be lowered by 5-10 dB SPL depending on the profile being considered.

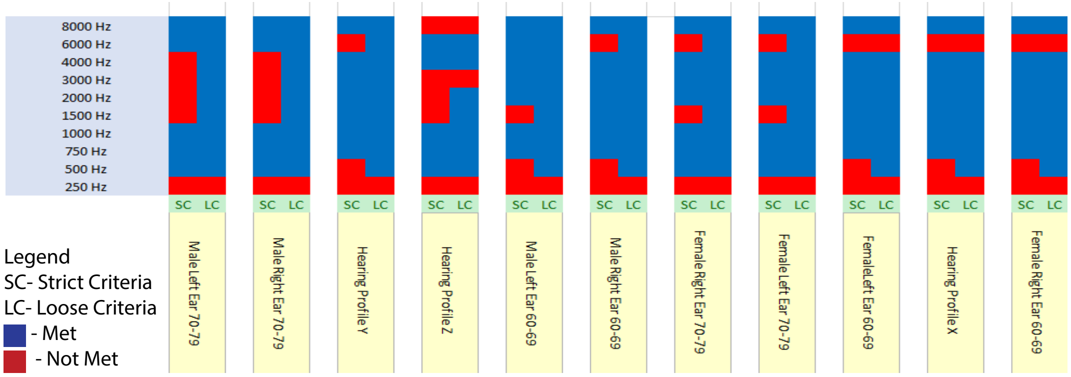

FIG. S3. Shows the quantification of fit in a visual matter for G.R.A.S KEMAR Response. It is organised by profiles in the horizontal axis, and the target frequencies on the vertical axis. Each profile has two sections denoting strict and loose criteria. The profiles are ordered from most number of blue squares to least number of blue squares. This is when the device is at full on gain, and shows that we do not necessarily need to lower the volume as the G.R.A.S KEMAR shows a better fit.

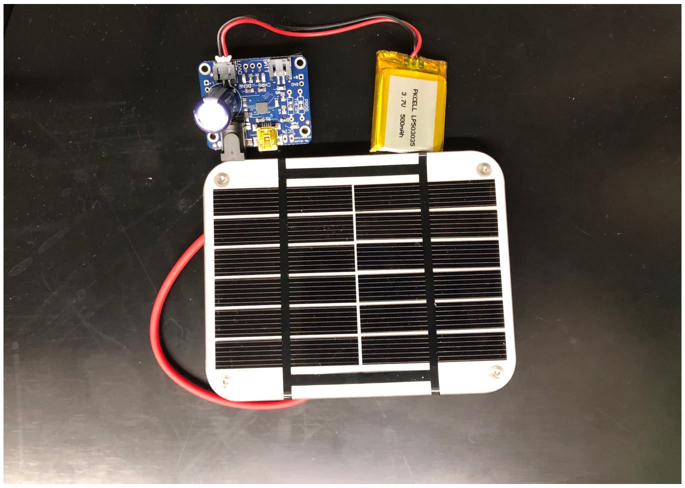

FIG. S4. Shows a solar panel, adapter, and lithium-ion packet battery for a rechargeable station. The costs are obtained: solar panel $1.85 (A Grade Small Solar Panel 1w 3w 5w 6v 9v 12v Solar Panel Low Price Mini Solar Panel from www.alibaba.com, from Shangdong, China, manufactured by Hinergy Energy, M/N HNP1W-5W6V); adapter $17.50 (USB / DC / Solar Lithium Ion/Polymer charger - v2 from www.adafruit.com, P/N 390), Lithium-ion packet battery $5.95 (Lithium Ion Polymer Battery with Short Cable - 3.7V 350mAh, from www.adafruit.com, P/N 4237).

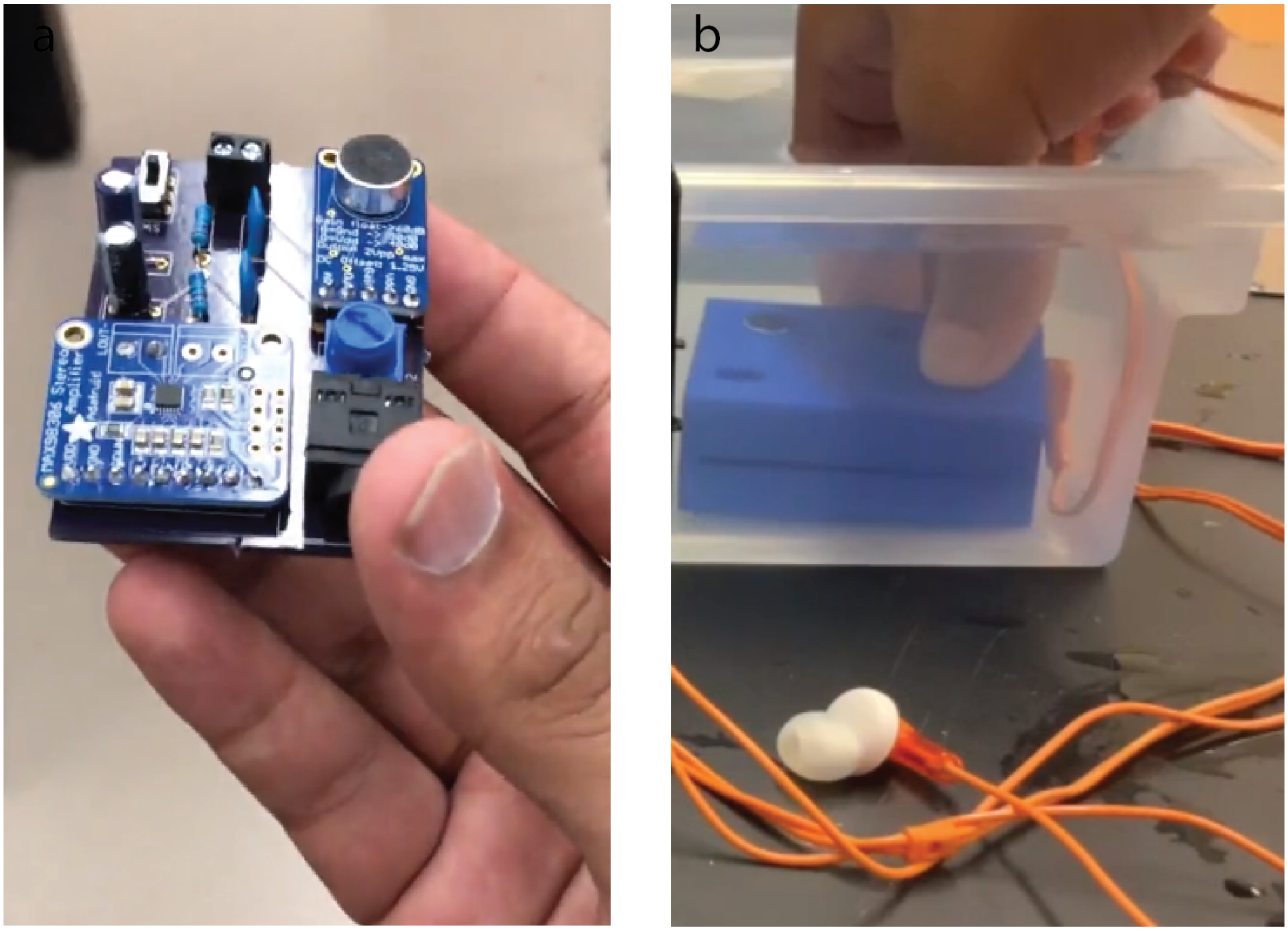

FIG. S5. **a** Still from SI Movie 4 detailing drop test of the device. **b** Still from SI Movie 3 detailing water test of the device.

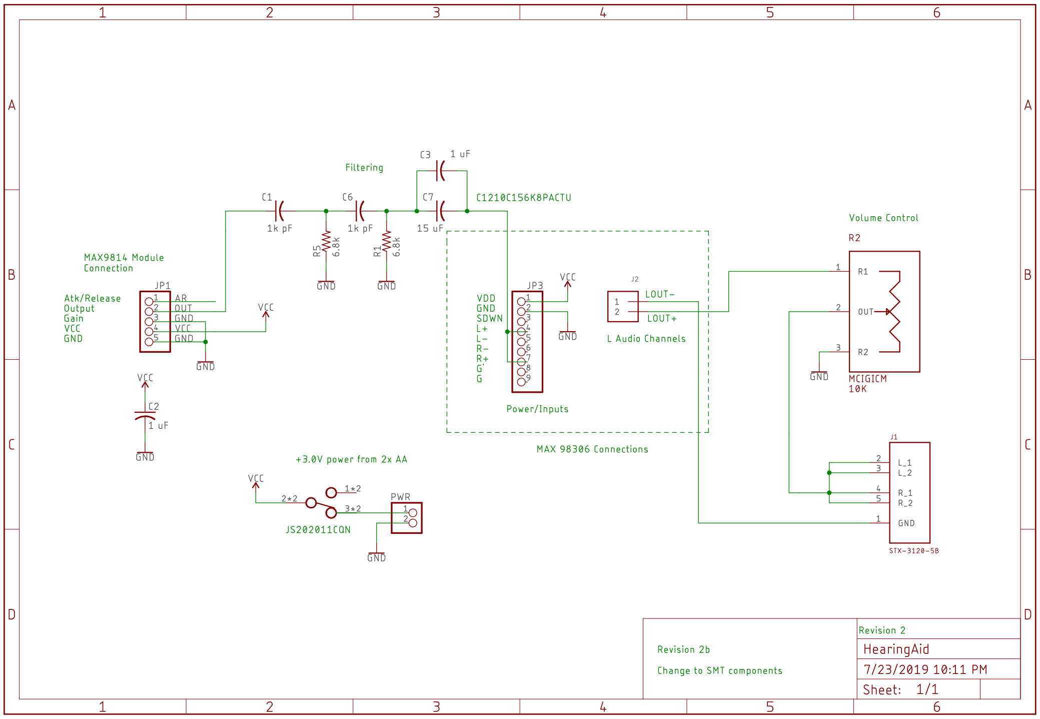

FIG. S6. Shows the schematic of the LoCHAid.

Small.PNG

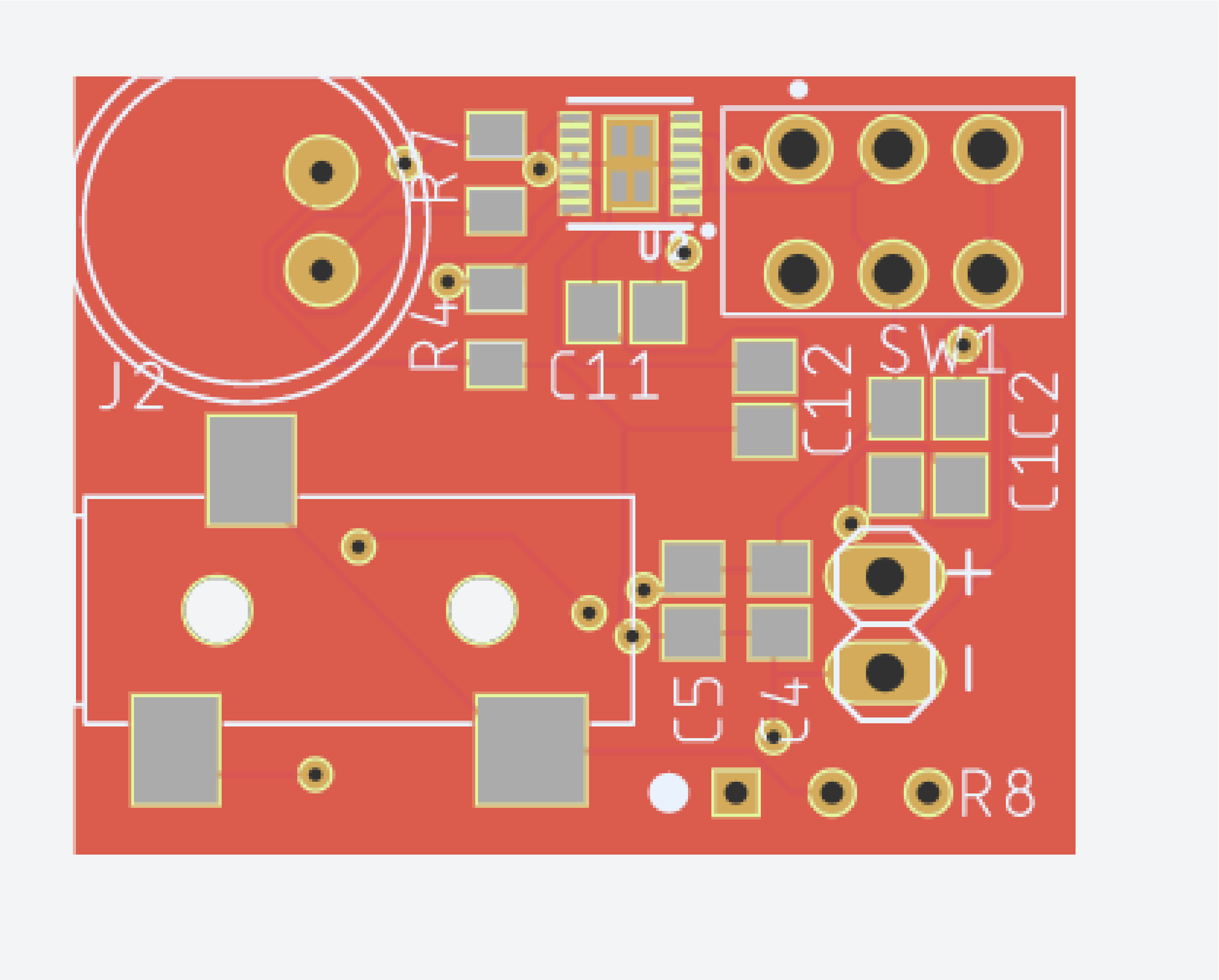

FIG. S7. Shows the PCB layout of a smaller version (1.05 x 0.81 in) of the LoCHAid. It cannot be hand assembled will need to be assembled by a third party fabrication house such as Macrofab.

PCB Actual.PNG

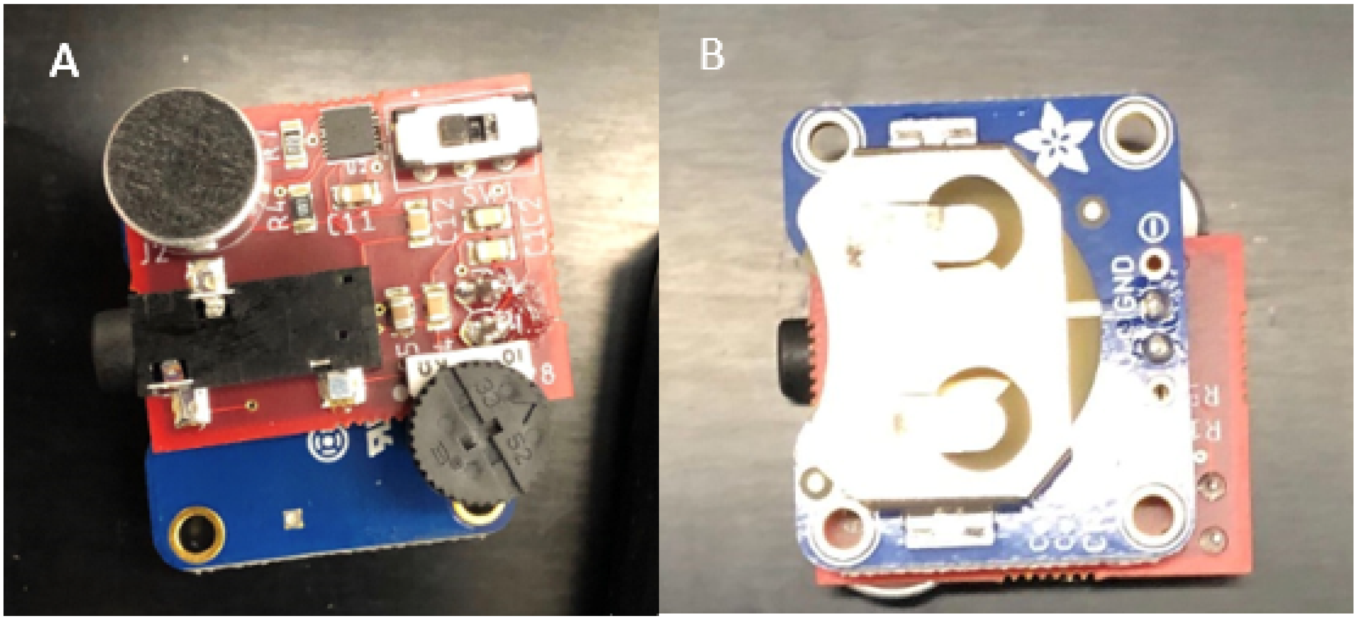

FIG. S8. A. Shows the front side view of the smaller version (1.05 x 0.81 in) of the LoCHAid. B. Shows the back side of the smaller version of the LoCHAid. It was assembled by third party fabrication house Macrofab (www.macrofab.com)

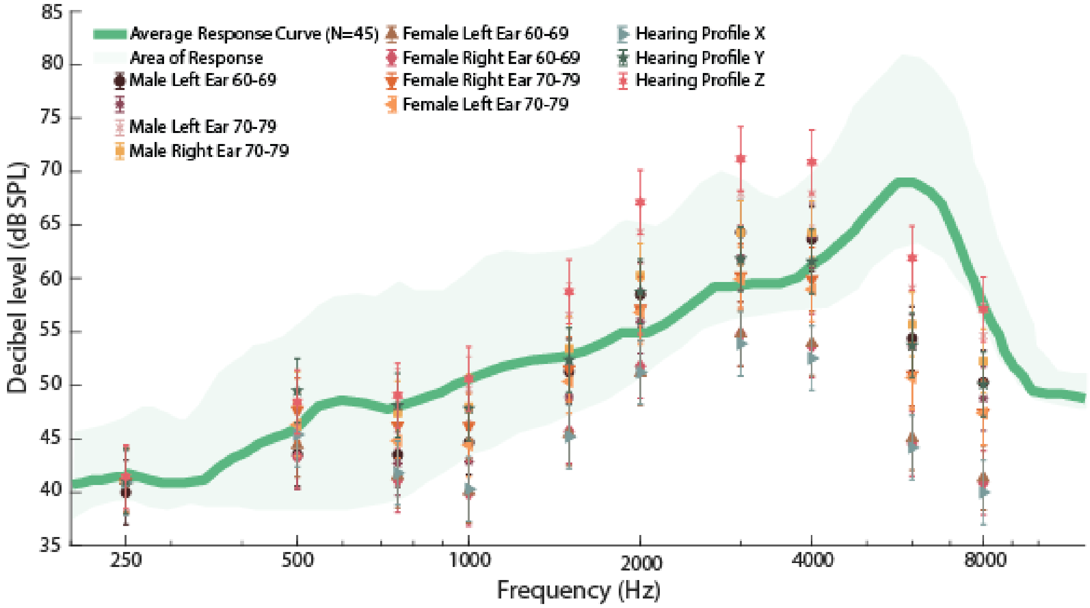

FIG. S9. Shows the ISTS Response (dB SPL) Curve for 55 dB SPL ISTS Input with Targets (dB SPL) for all profiles tested.

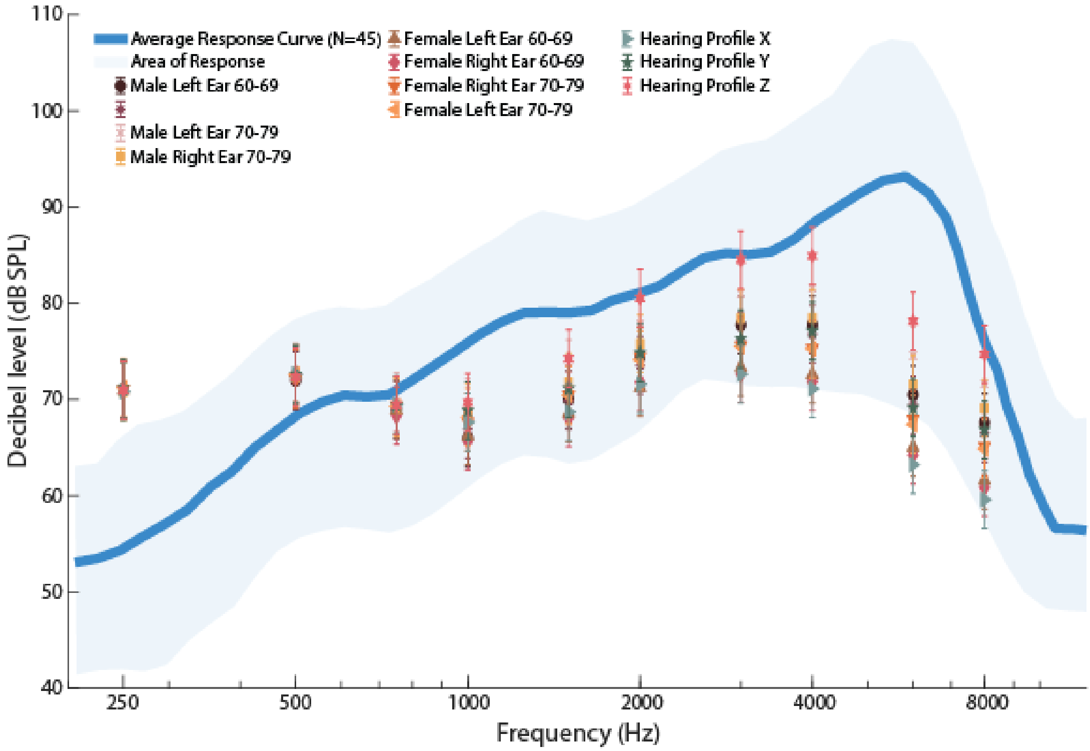

FIG. S10. Shows the ISTS Response (dB SPL) Curve for 80 dB SPL ISTS Input with Targets (dB SPL) for all profiles tested.

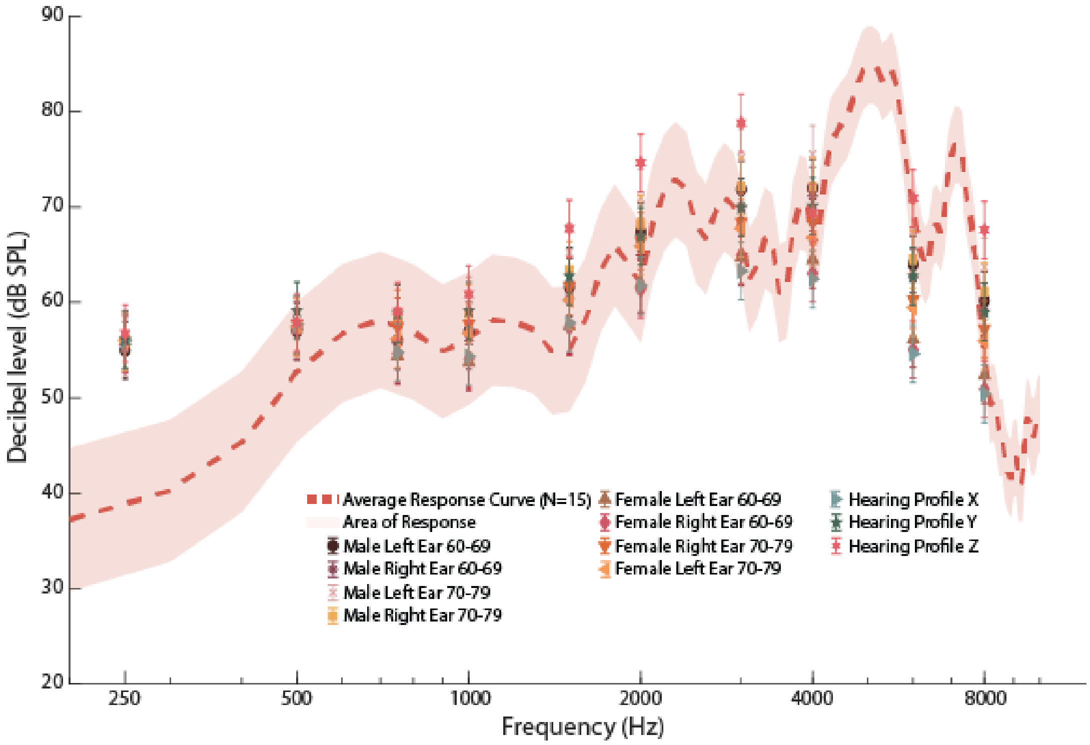

FIG. S11. Shows the KEMAR Response (dB SPL) Curve (Right Ear) for 65 dB SPL ISTS Input with Targets (dB SPL) for all profiles tested.

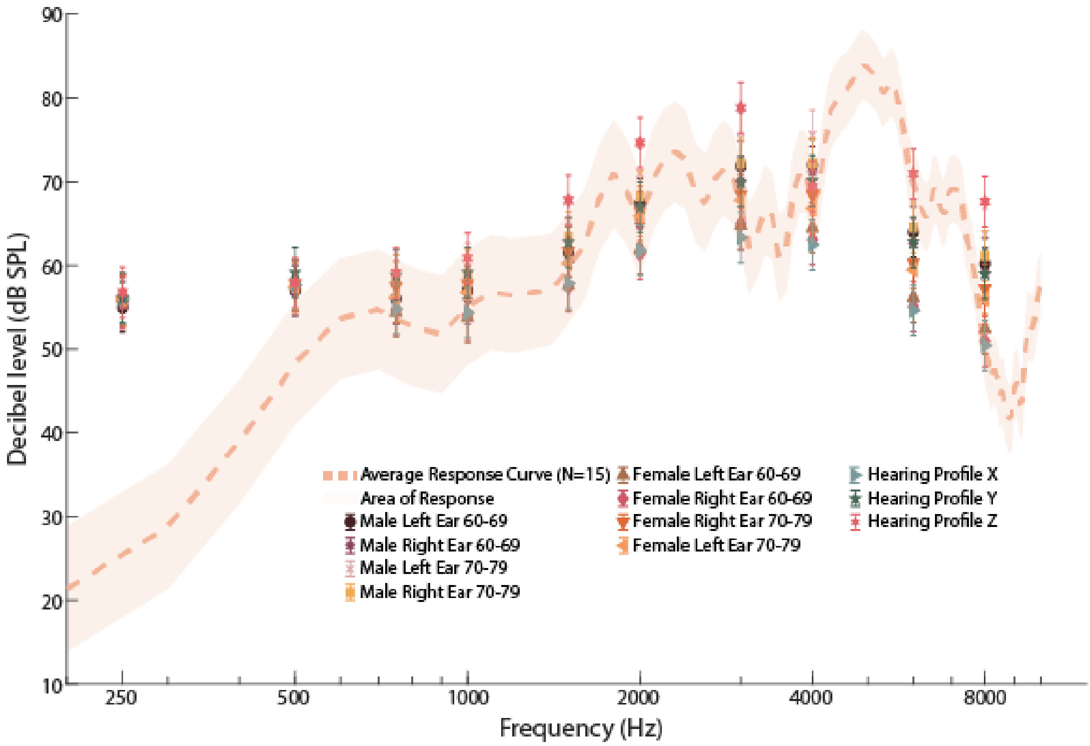

FIG. S12. Shows the KEMAR Response (dB SPL) Curve (Left Ear) for 65 dB SPL ISTS Input with Targets (dB SPL) for all profiles tested.

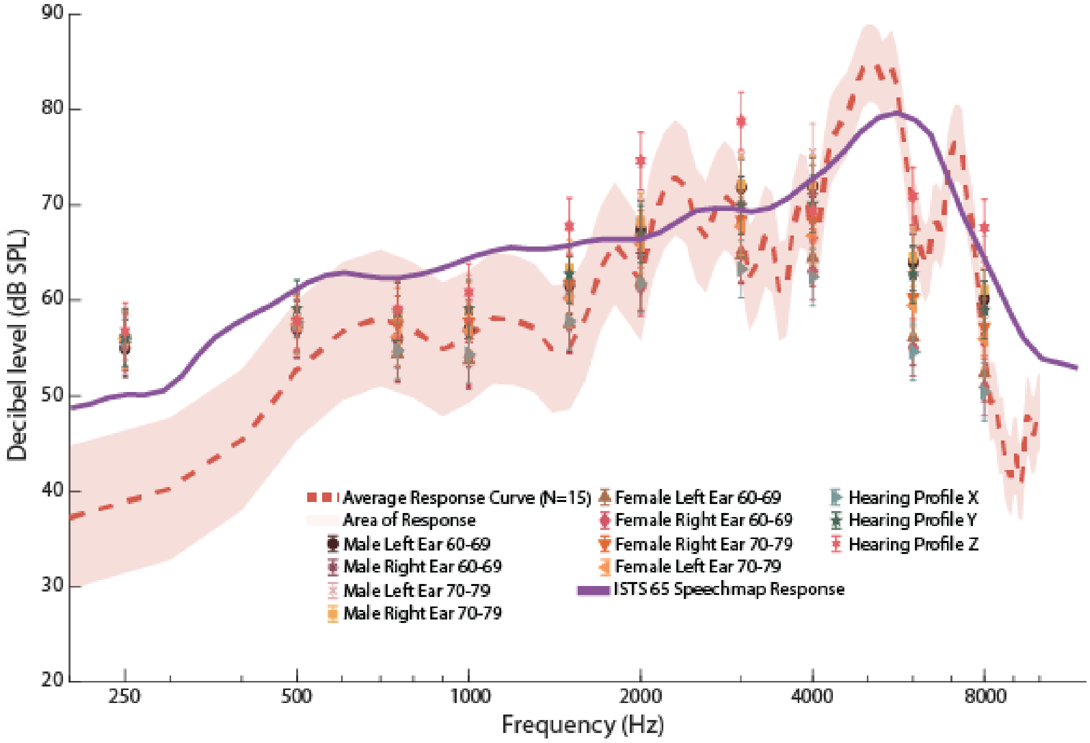

FIG. S13. Shows the KEMAR Response (dB SPL) Curve (Right Ear) for 65 dB SPL ISTS Input with Targets (dB SPL) for all profiles tested with ISTS 65 dB SPL Speechmap Response (dB SPL).

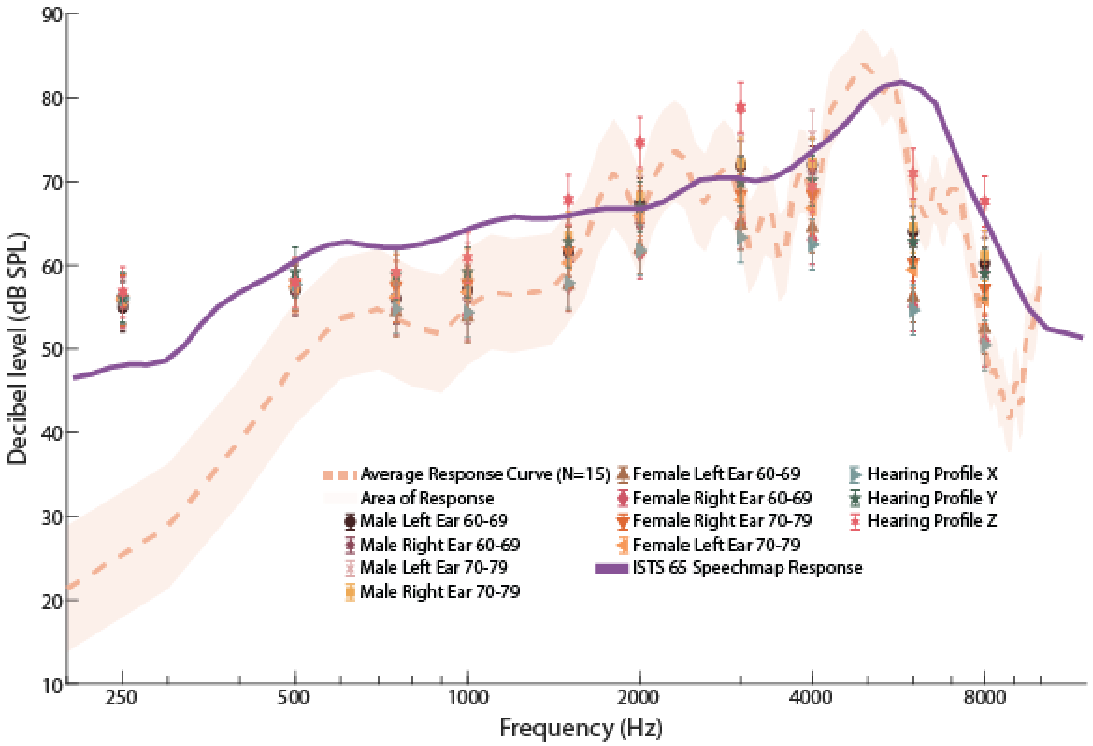

FIG. S14. Shows the KEMAR Response (dB SPL) Curve (Left Ear) for 65 dB SPL ISTS Input with Targets (dB SPL) for all profiles tested with ISTS 65 dB SPL Speechmap Response (dB SPL).

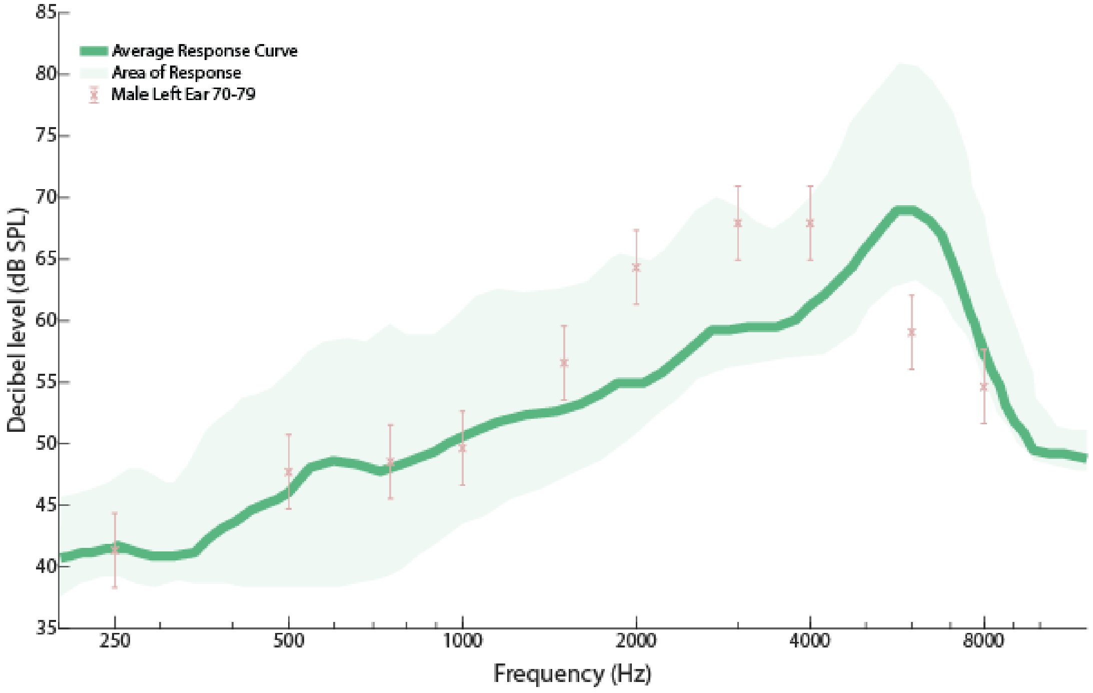

FIG. S15. Shows the ISTS 55 dB SPL Response (dB SPL) Curve with Male 60-69 Left Ear Targets (dB SPL).

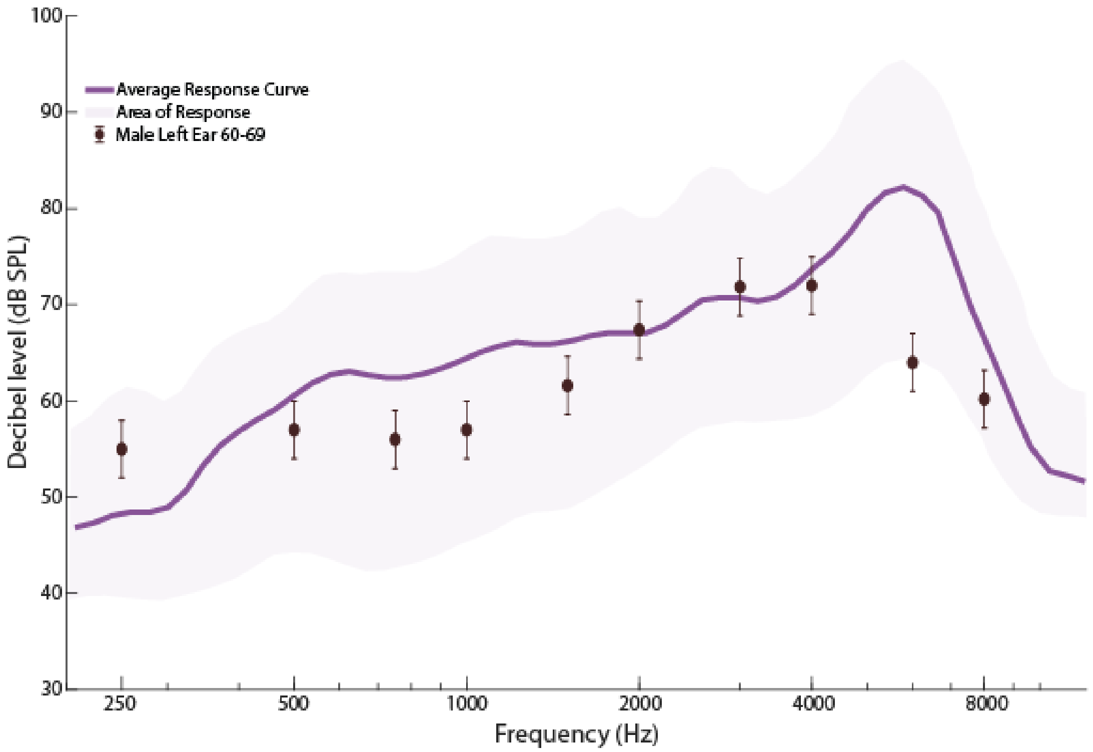

FIG. S16. Shows the ISTS 65 dB SPL Response (dB SPL) Curve with Male 60-69 Left Ear Targets (dB SPL).

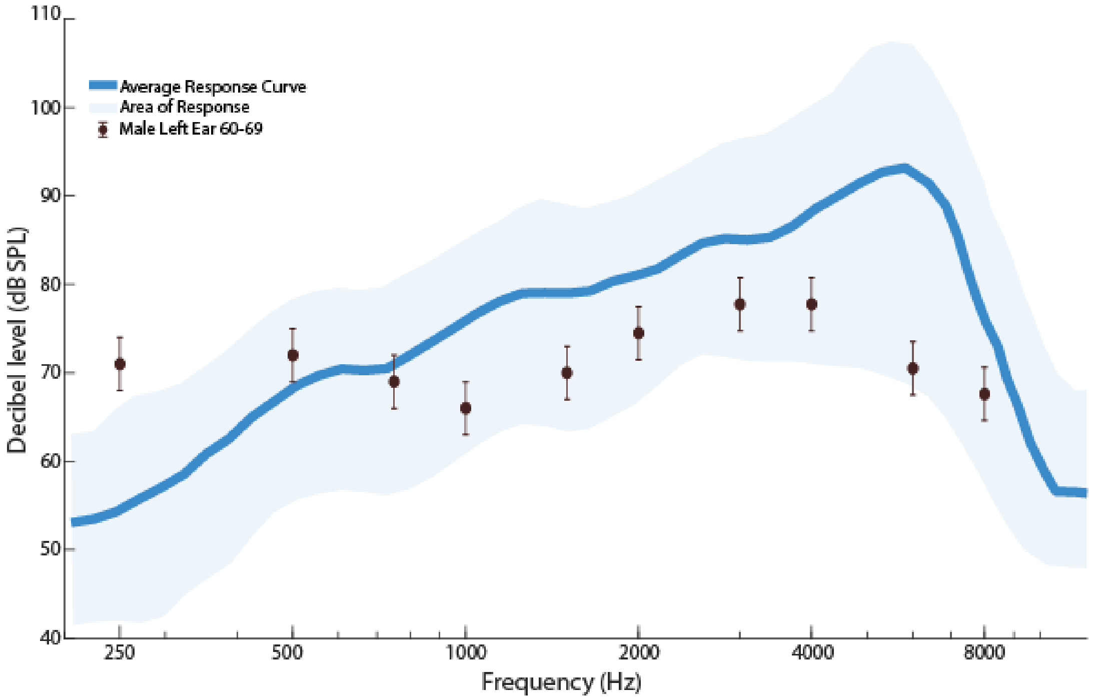

FIG. S17. Shows the ISTS 80 dB SPL Response (dB SPL) Curve with Male 60-69 Left Ear Targets (dB SPL).

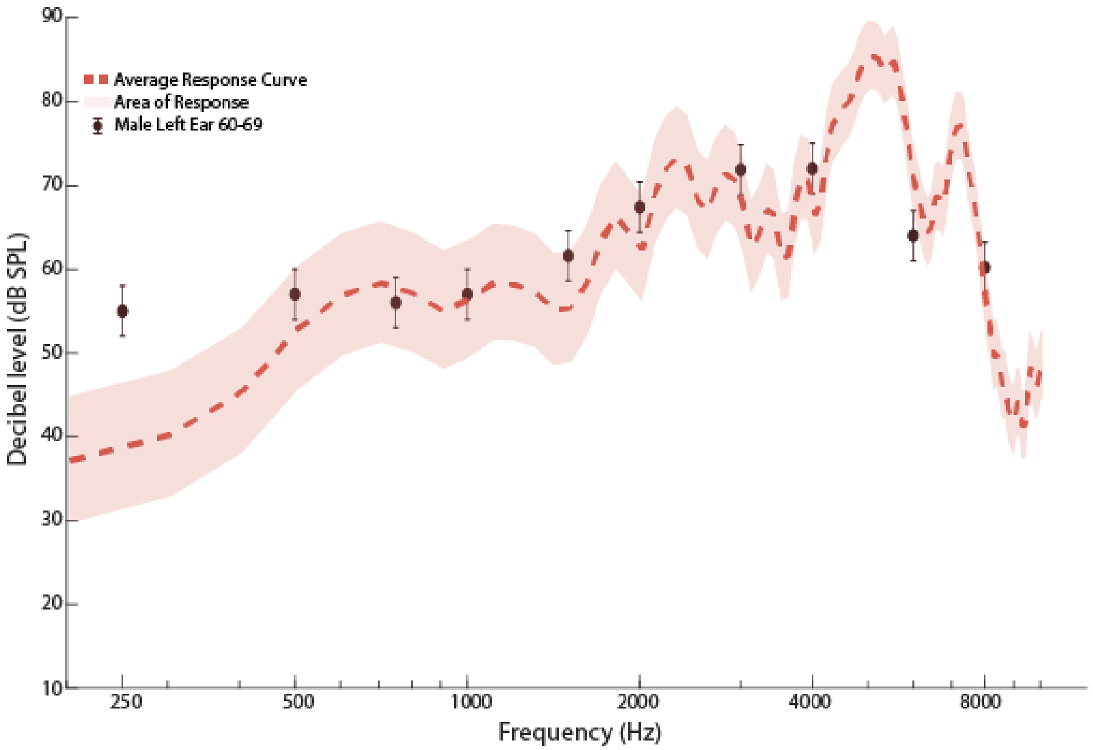

FIG. S18. Shows the G.R.A.S KEMAR 65 db SPL ISTS input Response (dB SPL) Curve (Right Ear) with Male 60-69 Left Ear Targets (dB SPL).

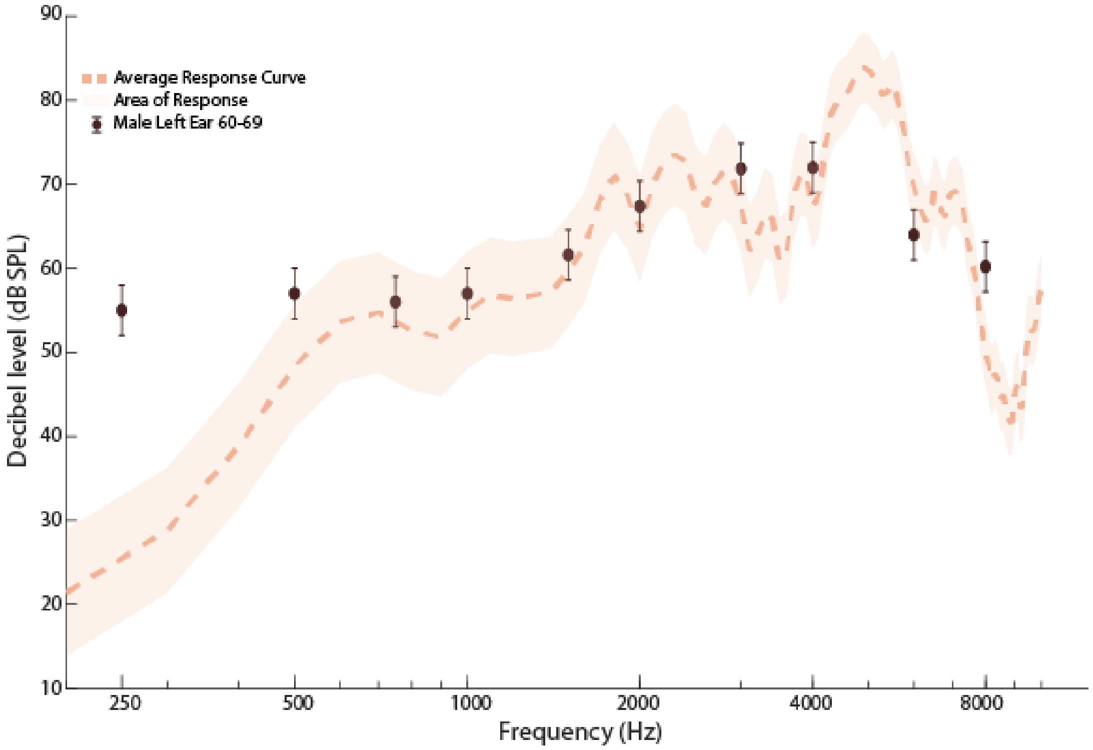

FIG. S19. Shows the G.R.A.S KEMAR 65 db SPL ISTS input Response (dB SPL) Curve (Left Ear) with Male 60-69 Left Ear Targets (dB SPL).

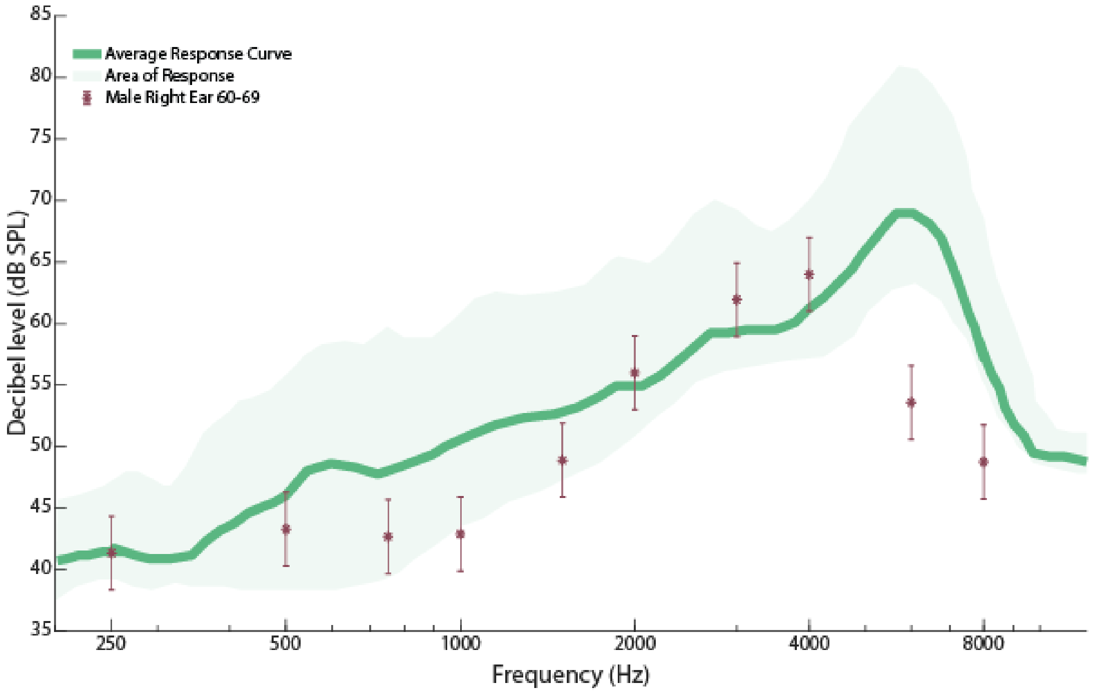

FIG. S20. Shows the ISTS 55 dB SPL Response (dB SPL) Curve with Male 60-69 Right Ear Targets (dB SPL).

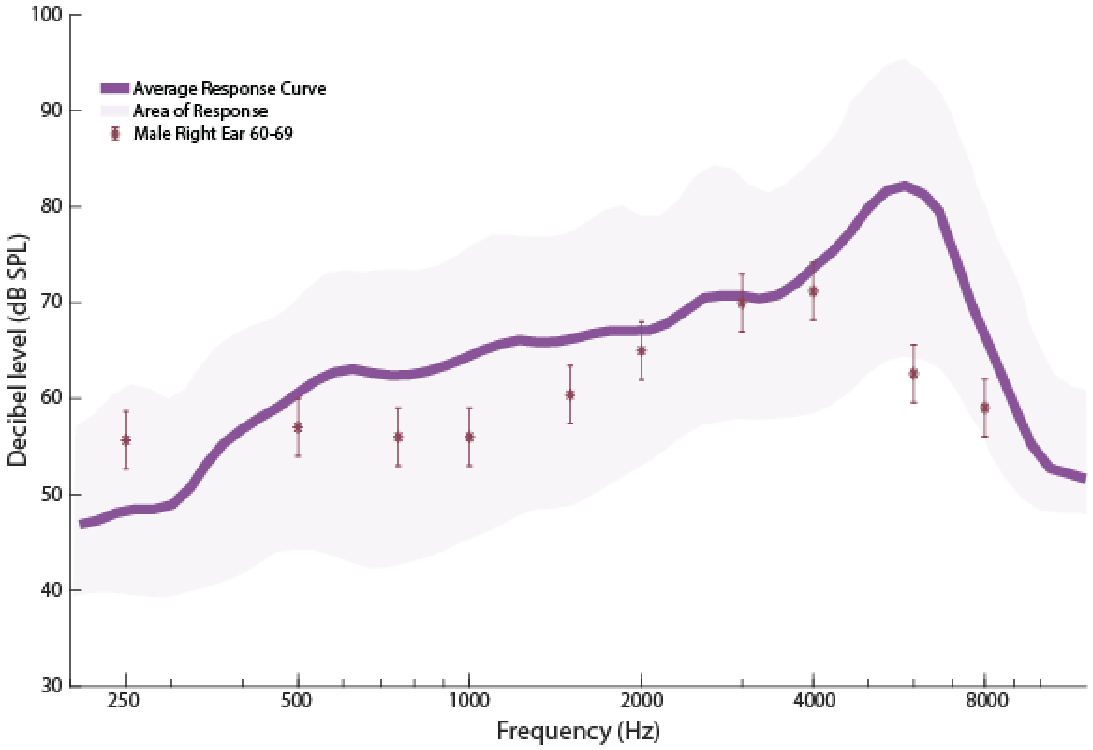

FIG. S21. Shows the ISTS 65 dB SPL Response (dB SPL) Curve with Male 60-69 Right Ear Targets (dB SPL).

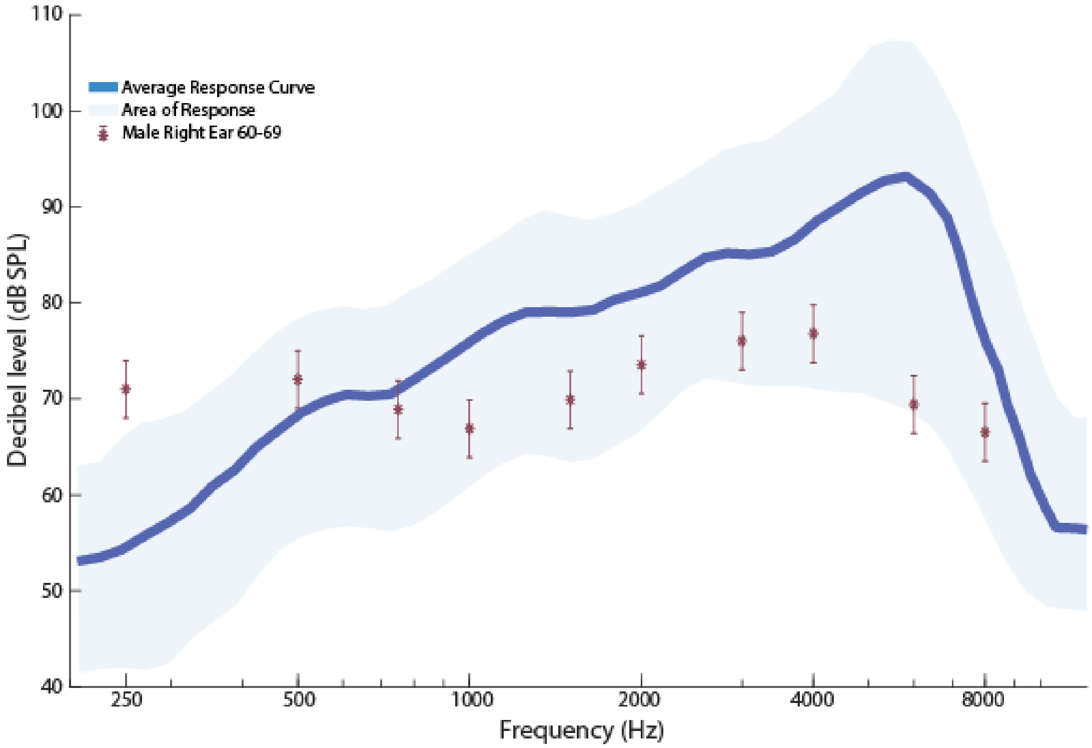

FIG. S22. Shows the ISTS 80 dB SPL Response (dB SPL) Curve with Male 60-69 Right Ear Targets (dB SPL).

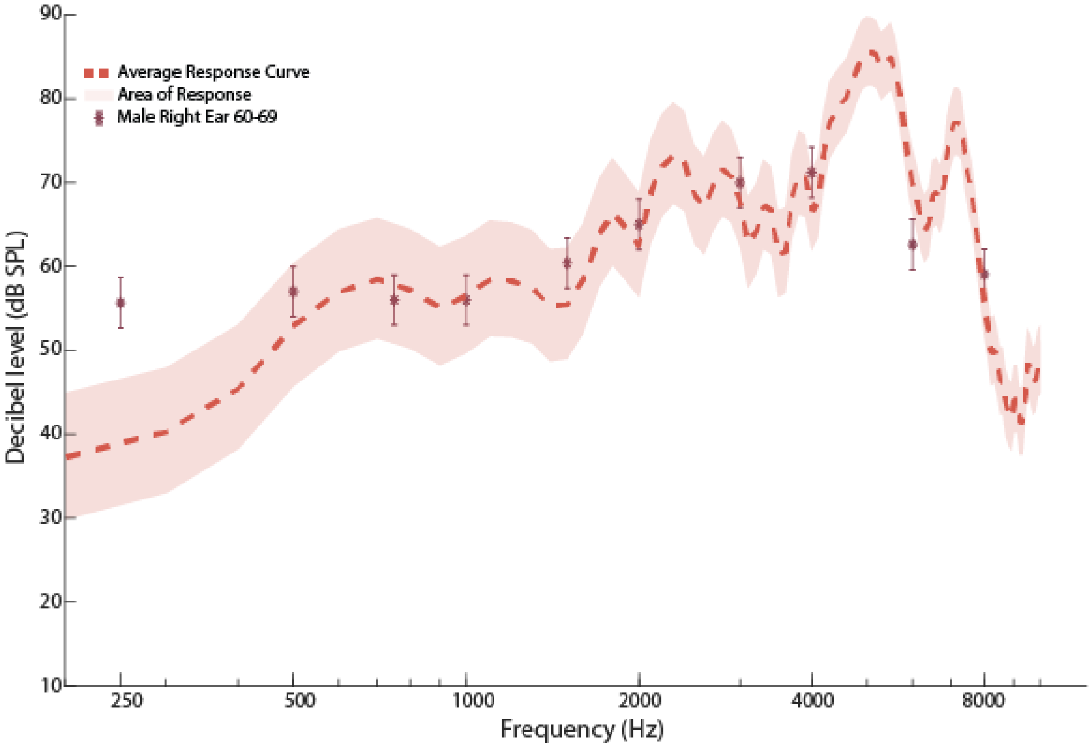

FIG. S23. Shows the G.R.A.S KEMAR 65 db SPL ISTS input Response (dB SPL) Curve (Right Ear) with Male 60-69 Right Ear Targets (dB SPL).

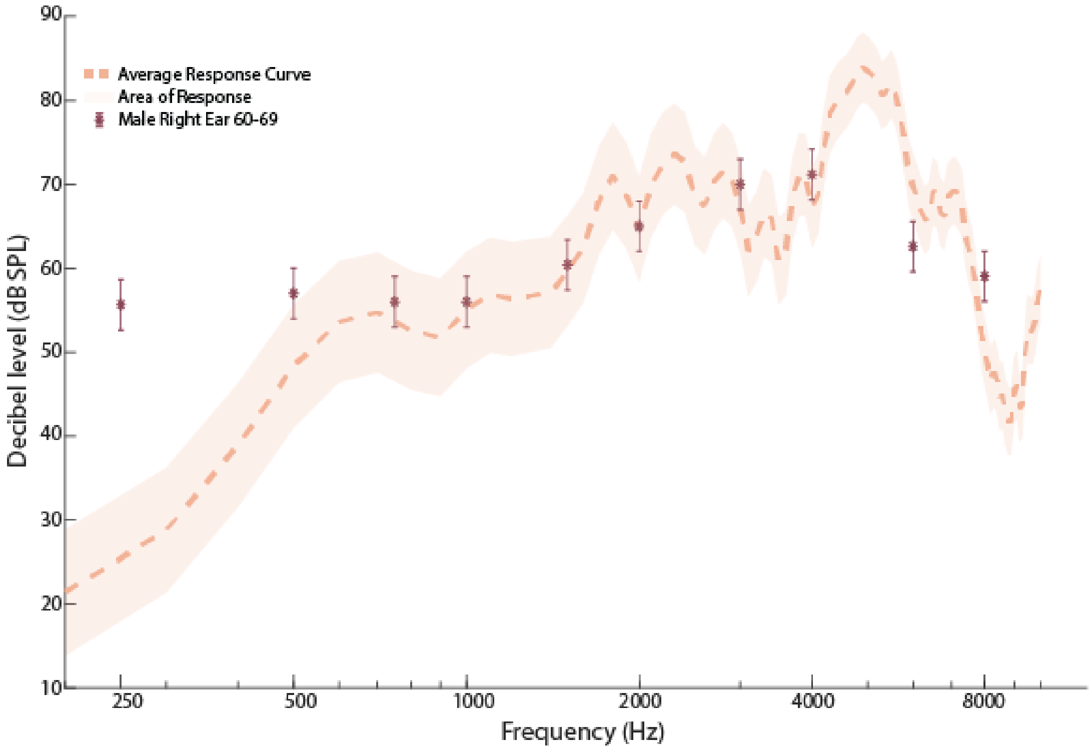

FIG. S24. Shows the G.R.A.S KEMAR 65 db SPL ISTS input Response (dB SPL) Curve (Left Ear) with Male 60-69 Right Ear Targets (dB SPL).

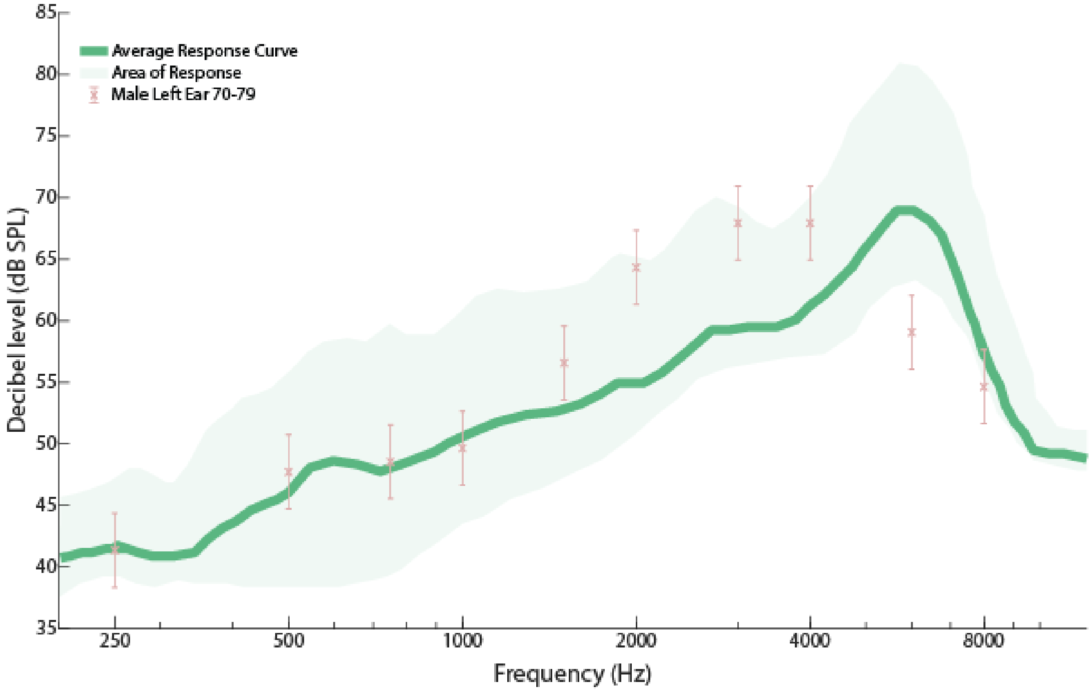

FIG. S25. Shows the ISTS 55 dB SPL Response (dB SPL) Curve with Male 70-79 Left Ear Targets (dB SPL).

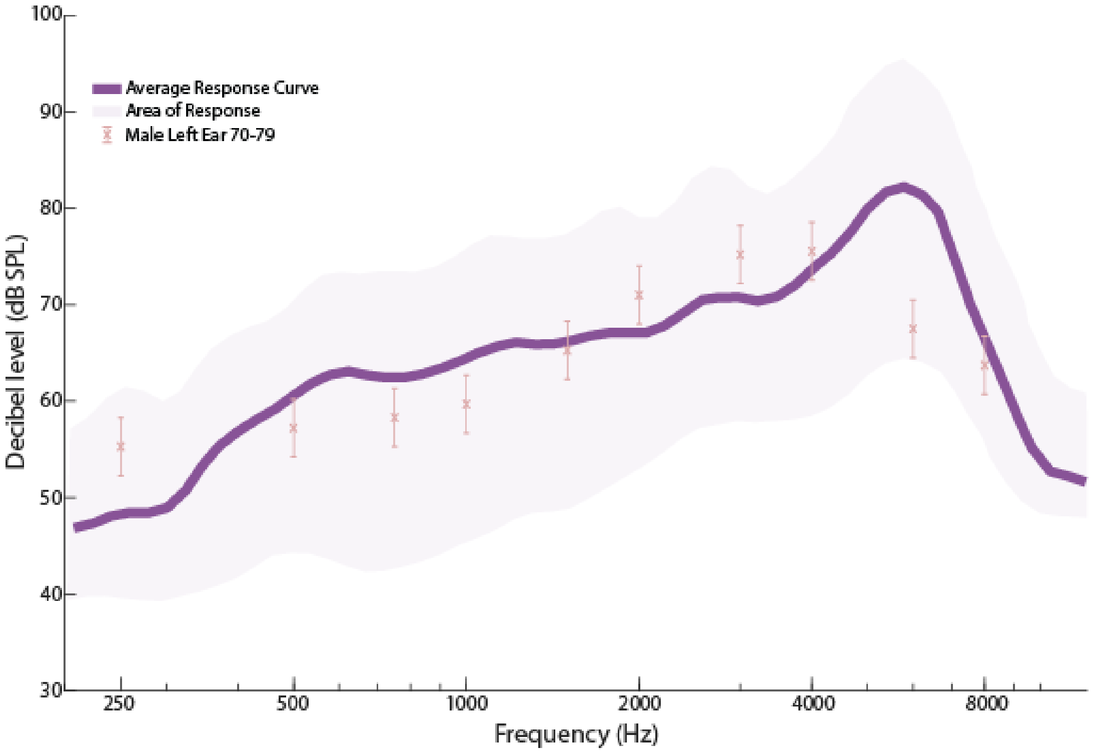

FIG. S26. Shows the ISTS 65 dB SPL Response (dB SPL) Curve with Male 70-79 Left Ear Targets (dB SPL).

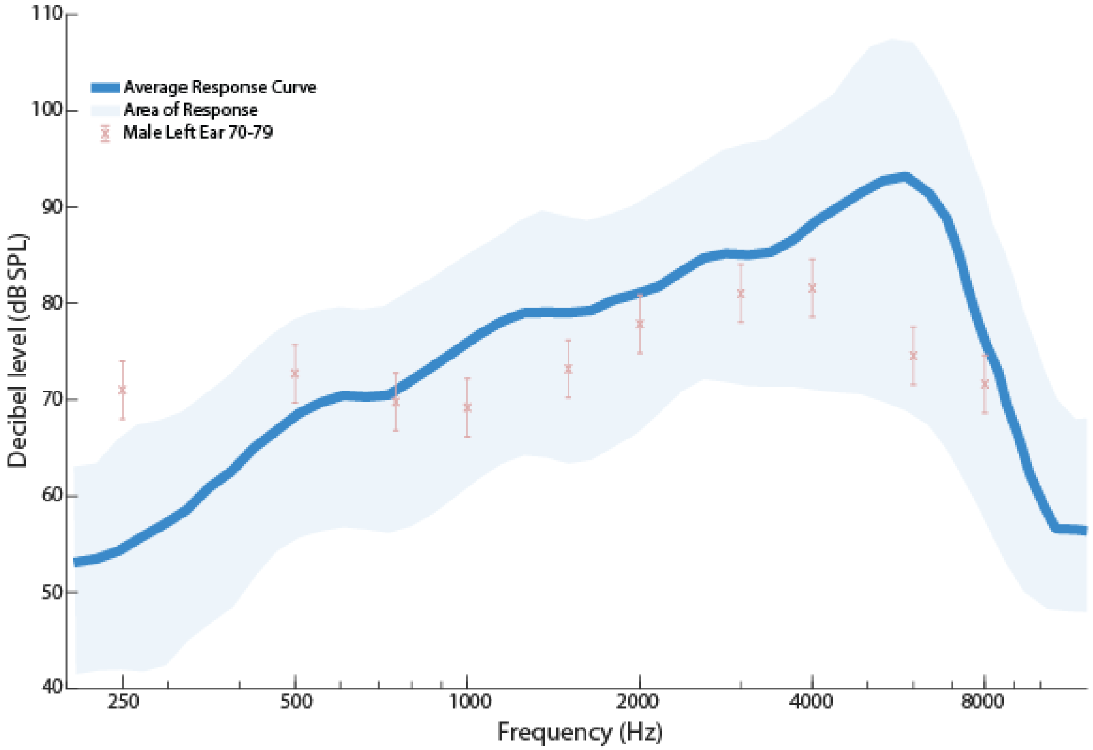

FIG. S27. Shows the ISTS 80 dB SPL Response (dB SPL) Curve with Male 70-79 Left Ear Targets (dB SPL).

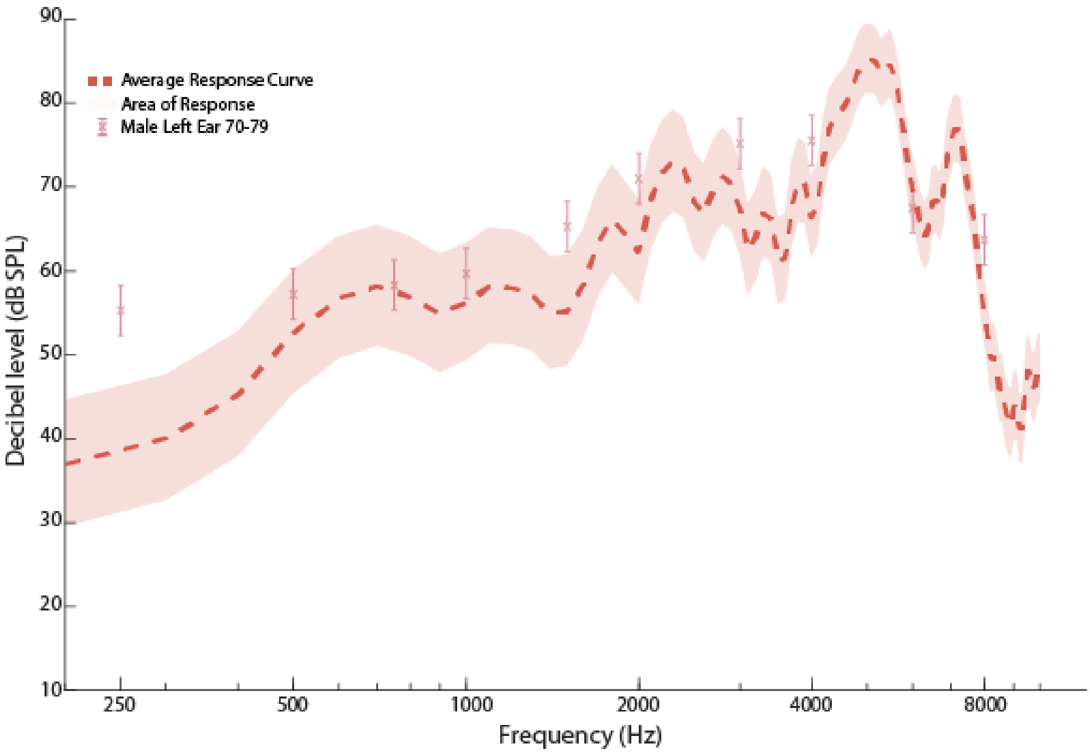

FIG. S28. Shows the G.R.A.S KEMAR 65 db SPL ISTS input Response (dB SPL) Curve (Right Ear) with Male 70-79 Left Ear Targets (dB SPL).

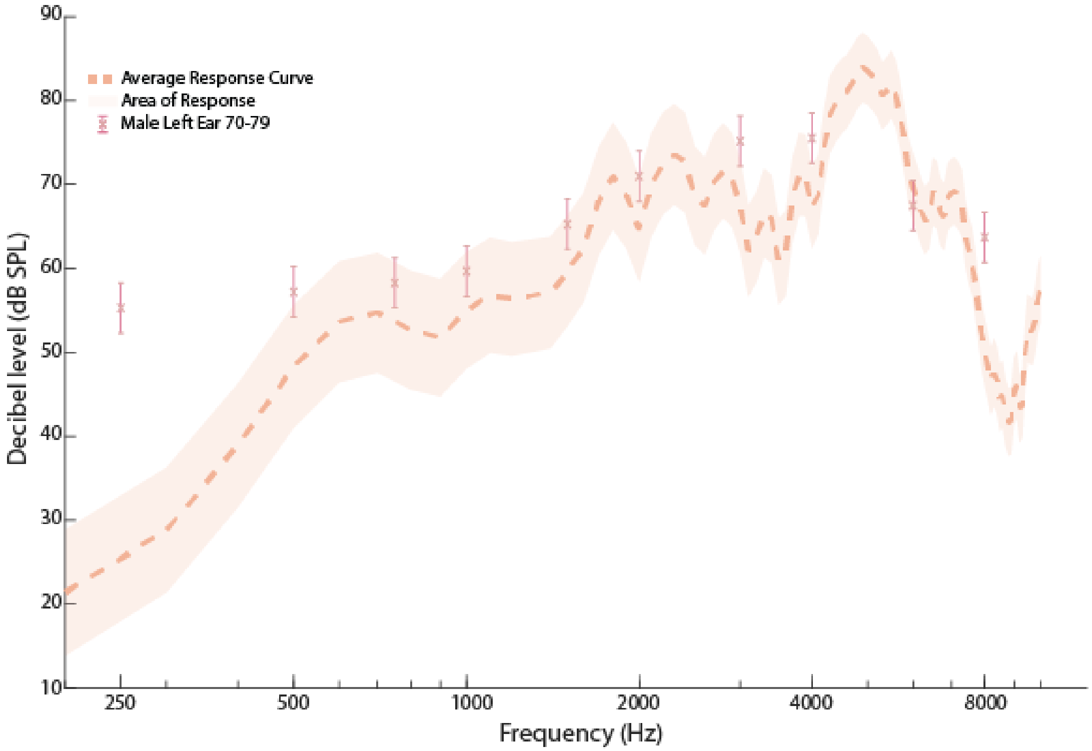

FIG. S29. Shows the G.R.A.S KEMAR 65 db SPL ISTS input Response (dB SPL) Curve (Left Ear) with Male 70-79 Left Ear Targets (dB SPL).

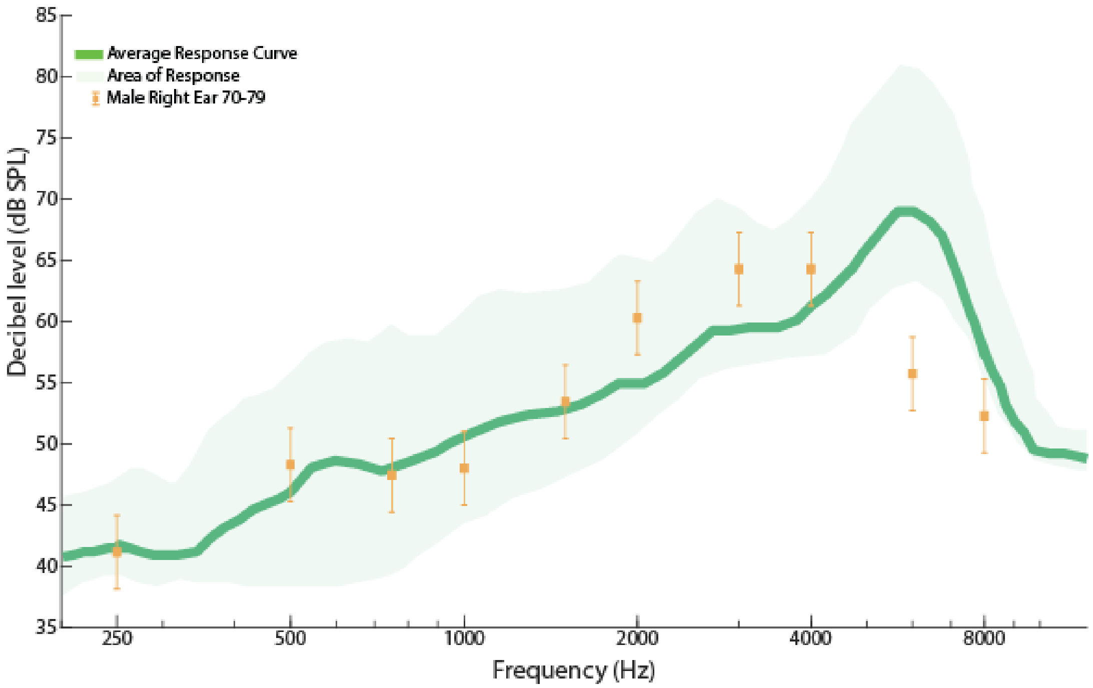

FIG. S30. Shows the ISTS 55 dB SPL Response (dB SPL) Curve with Male 70-79 Right Ear Targets (dB SPL).

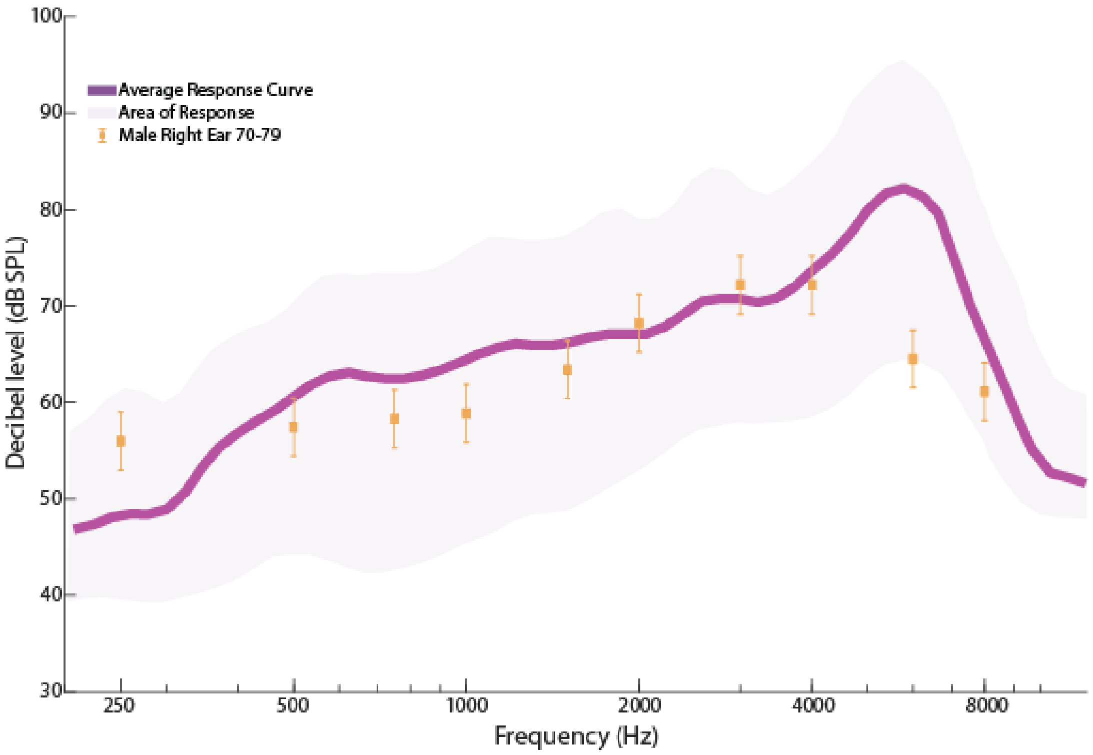

FIG. S31. Shows the ISTS 65 dB SPL Response (dB SPL) Curve with Male 70-79 Right Ear Targets (dB SPL).

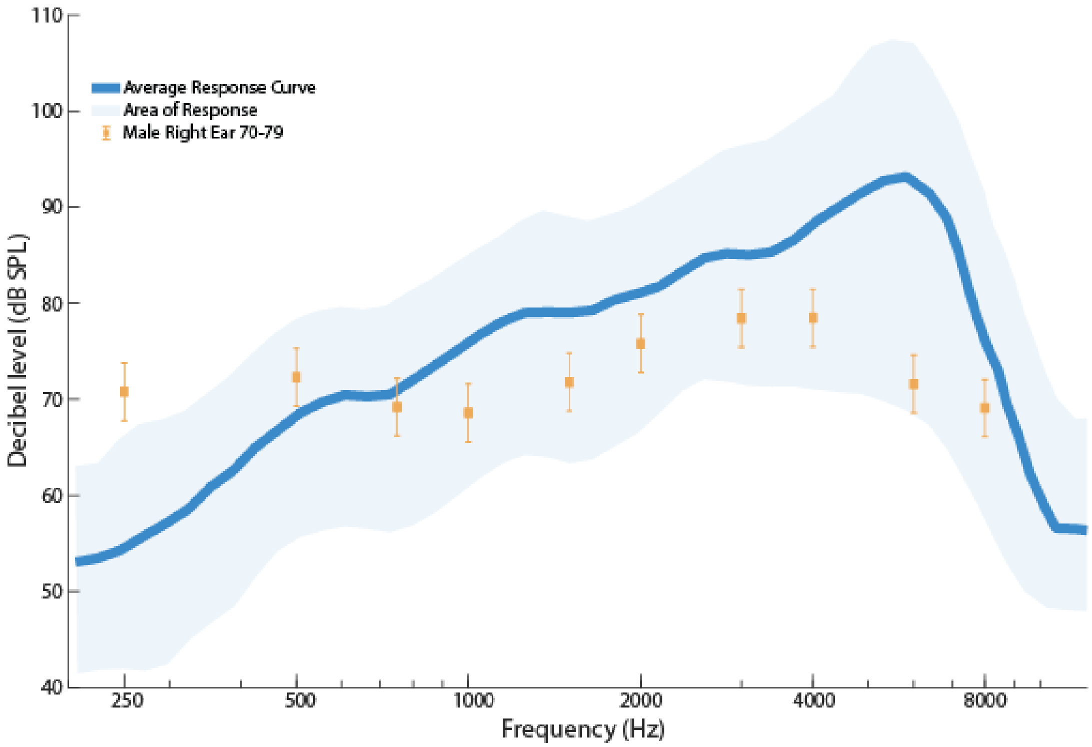

FIG. S32. Shows the ISTS 80 dB SPL Response (dB SPL) Curve with Male 70-79 Right Ear Targets (dB SPL).

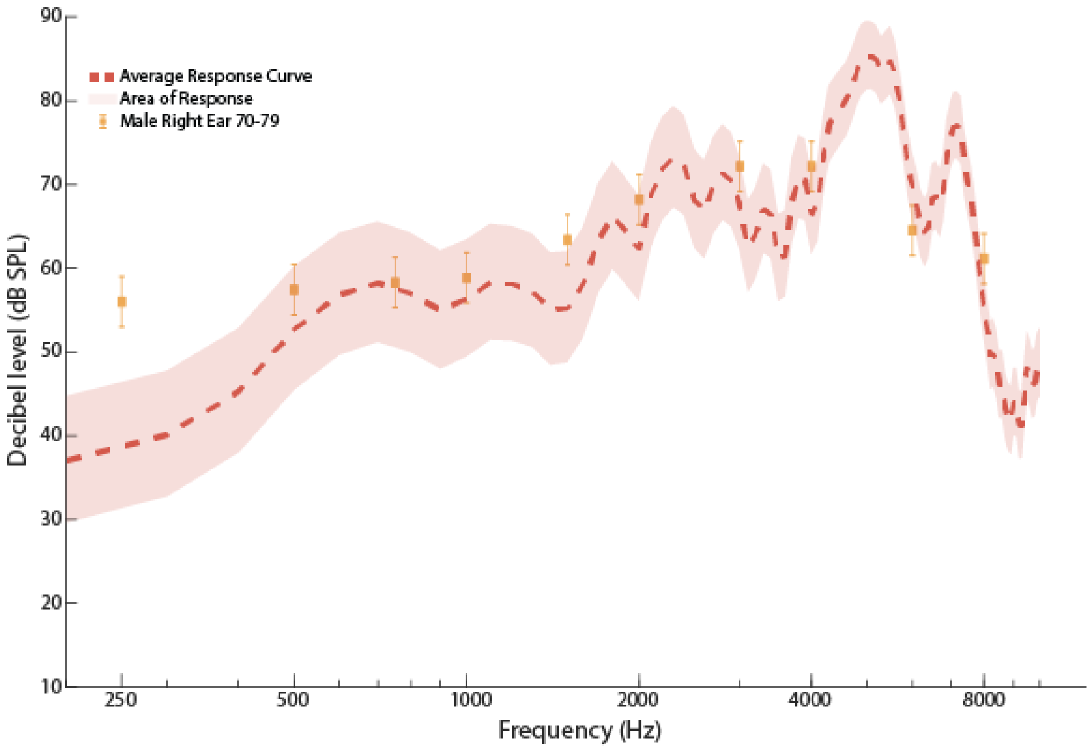

FIG. S33. Shows the G.R.A.S KEMAR 65 db SPL ISTS input Response (dB SPL) Curve (Right Ear) with Male 70-79 Right Ear Targets (dB SPL).

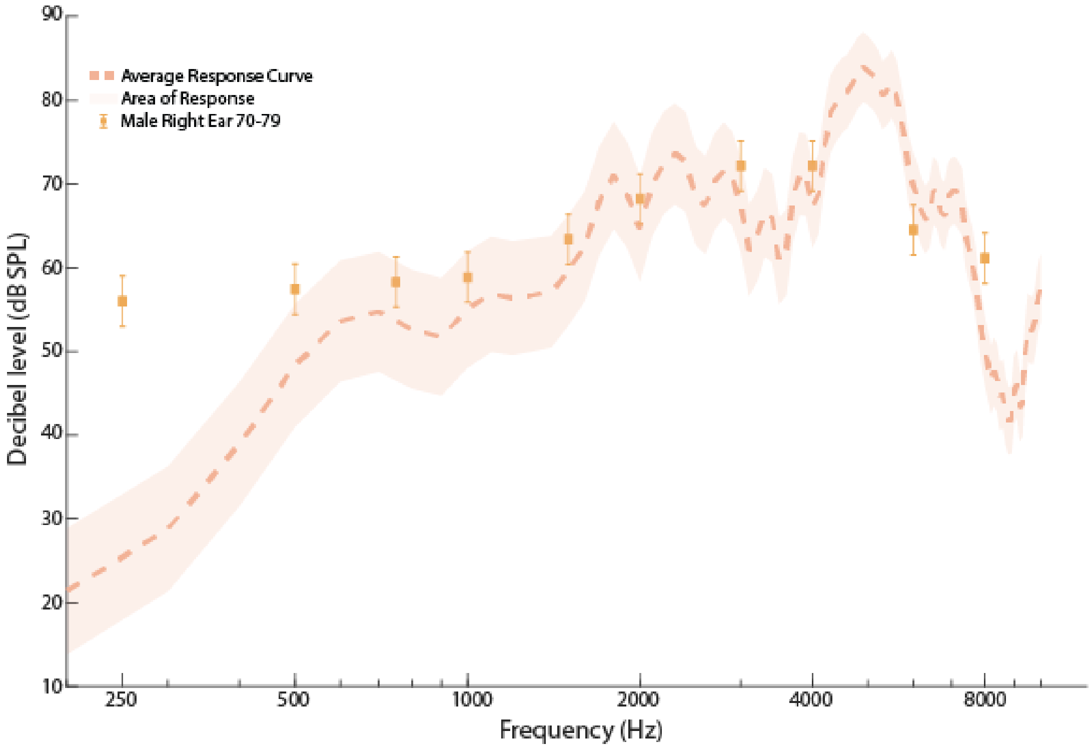

FIG. S34. Shows the G.R.A.S KEMAR 65 db SPL ISTS input Response (dB SPL) Curve (Left Ear) with Male 70-79 Right Ear Targets (dB SPL).

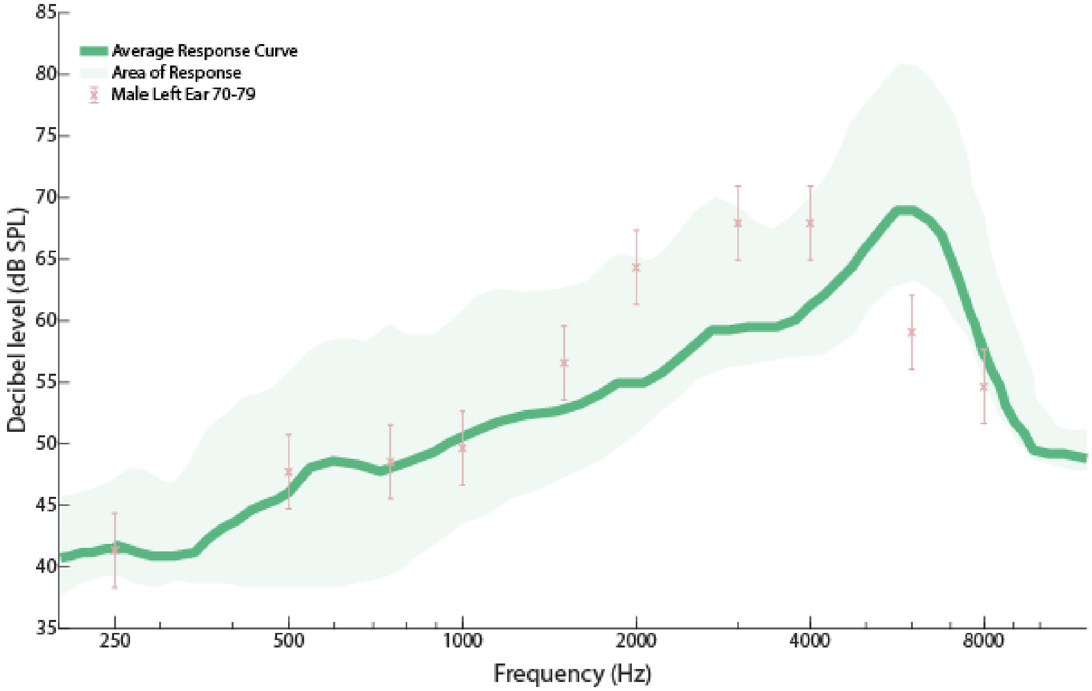

FIG. S35. Shows the ISTS 55 dB SPL Response (dB SPL) Curve with Male 70-79 Left Ear Targets (dB SPL).

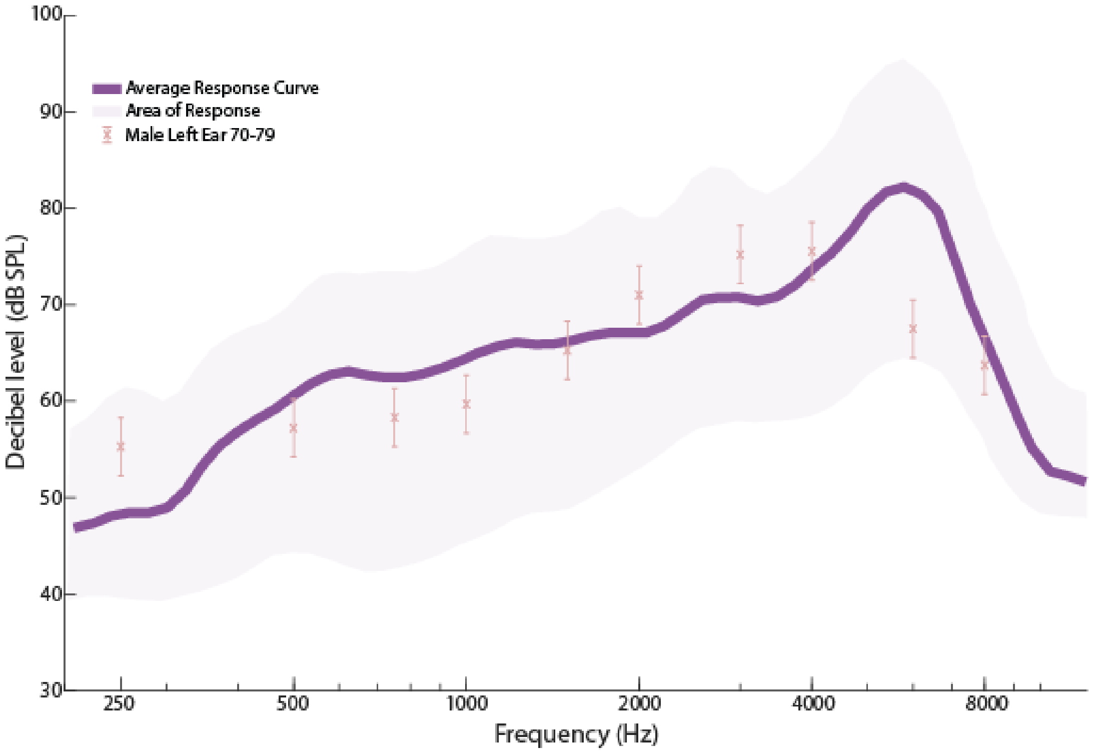

FIG. S36. Shows the ISTS 65 dB SPL Response (dB SPL) Curve with Male 70-79 Left Ear Targets (dB SPL).

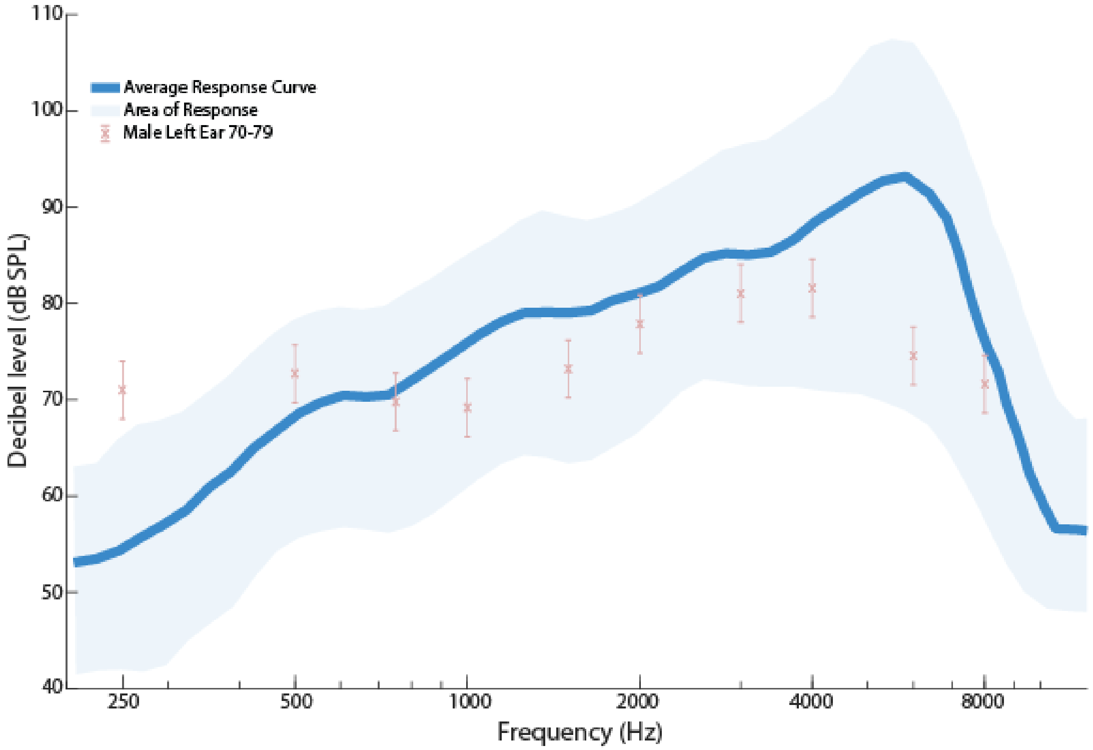

FIG. S37. Shows the ISTS 80 dB SPL Response (dB SPL) Curve with Male 70-79 Left Ear Targets (dB SPL).

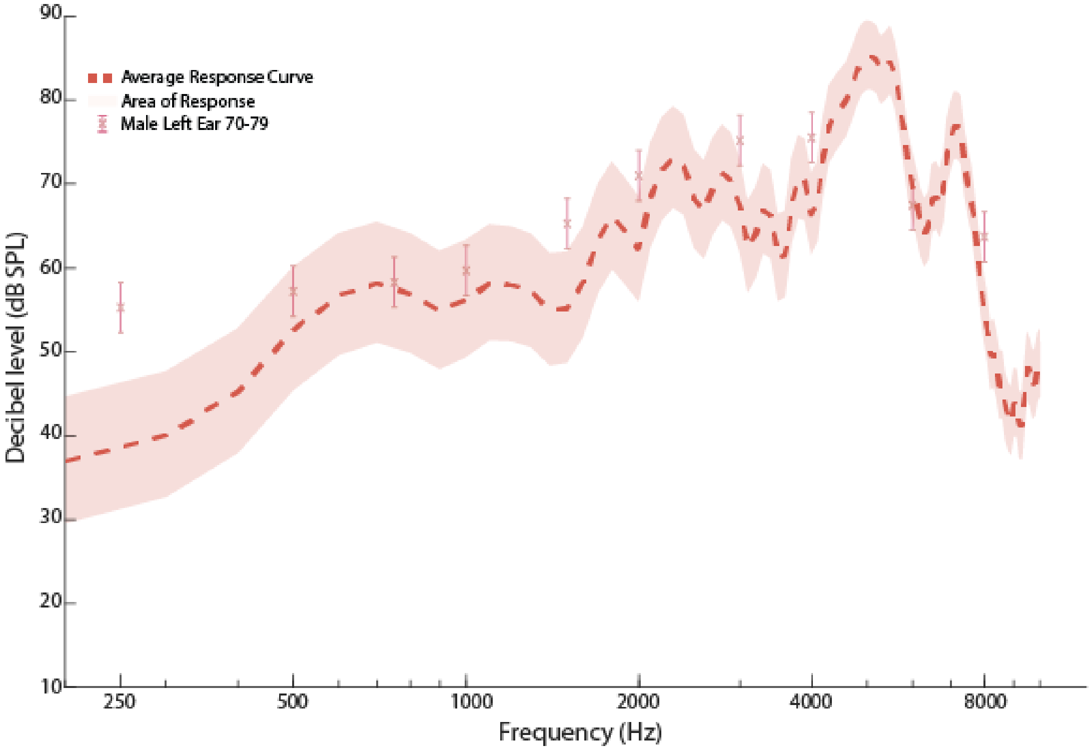

FIG. S38. Shows the G.R.A.S KEMAR 65 db SPL ISTS input Response (dB SPL) Curve (Right Ear) with Male 70-79 Left Ear Targets (dB SPL).

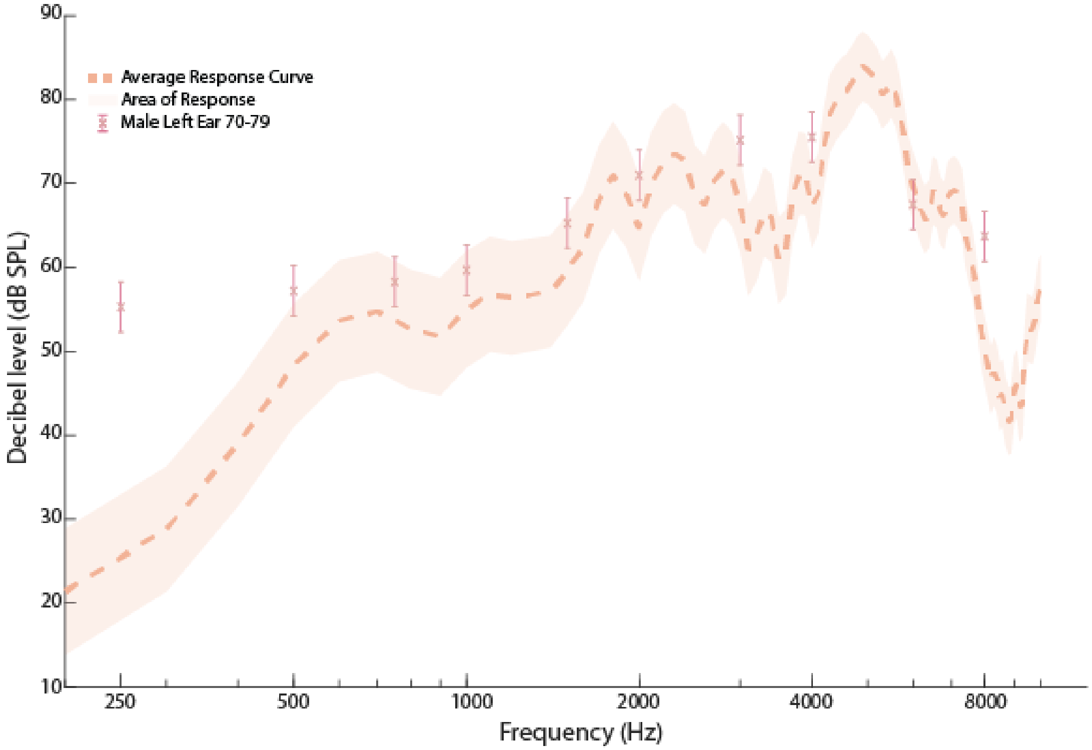

FIG. S39. Shows the G.R.A.S KEMAR 65 db SPL ISTS input Response (dB SPL) Curve (Left Ear) with Male 70-79 Left Ear Targets (dB SPL).

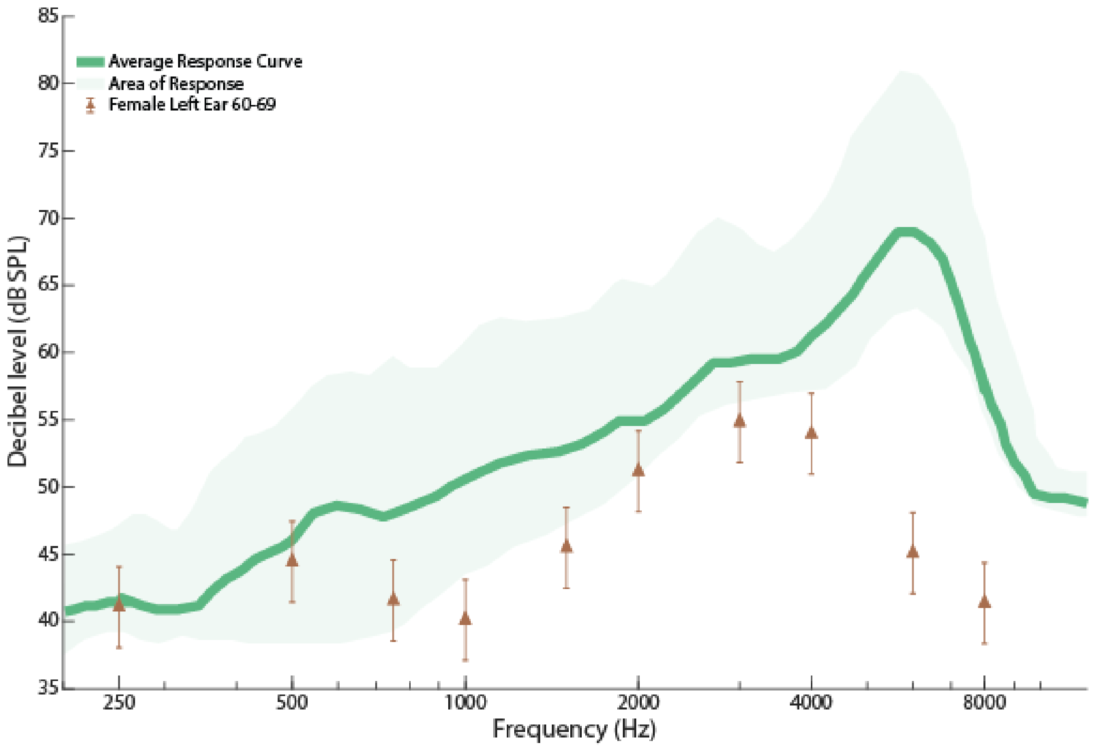

FIG. S40. Shows the ISTS 55 dB SPL Response (dB SPL) Curve with Female 60-69 Left Ear Targets (dB SPL).

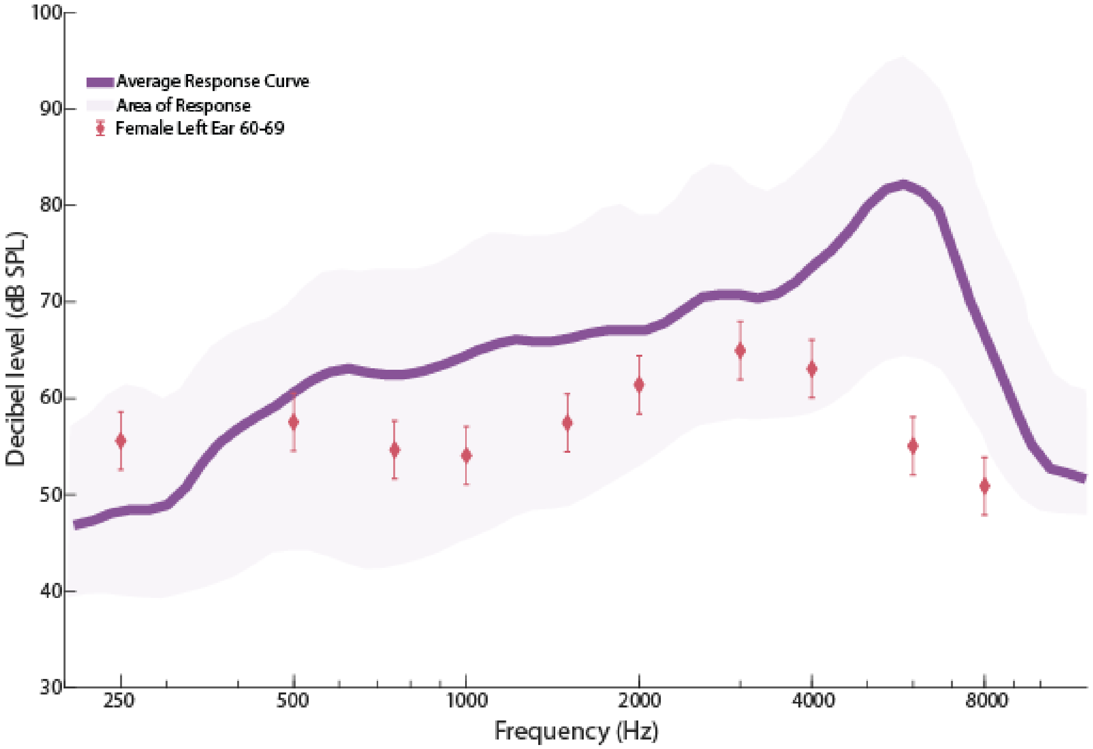

FIG. S41. Shows the ISTS 65 dB SPL Response (dB SPL) Curve with Female 60-69 Left Ear Targets (dB SPL).

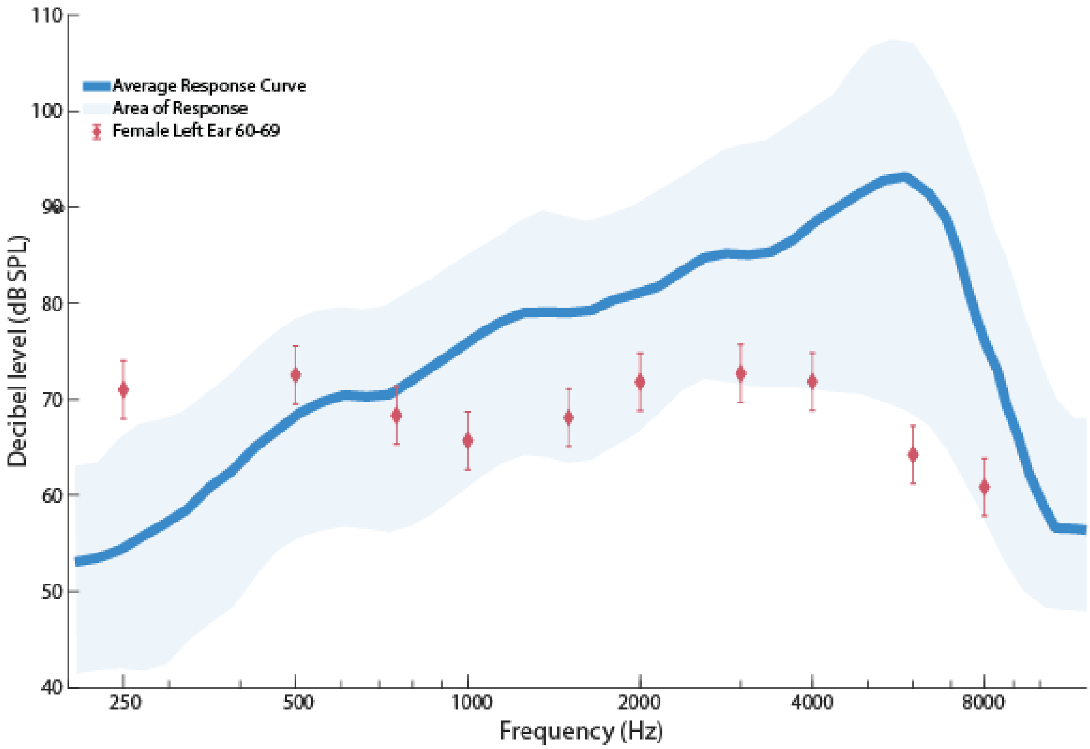

FIG. S42. Shows the ISTS 80 dB SPL Response (dB SPL) Curve with Female 60-69 Left Ear Targets (dB SPL).

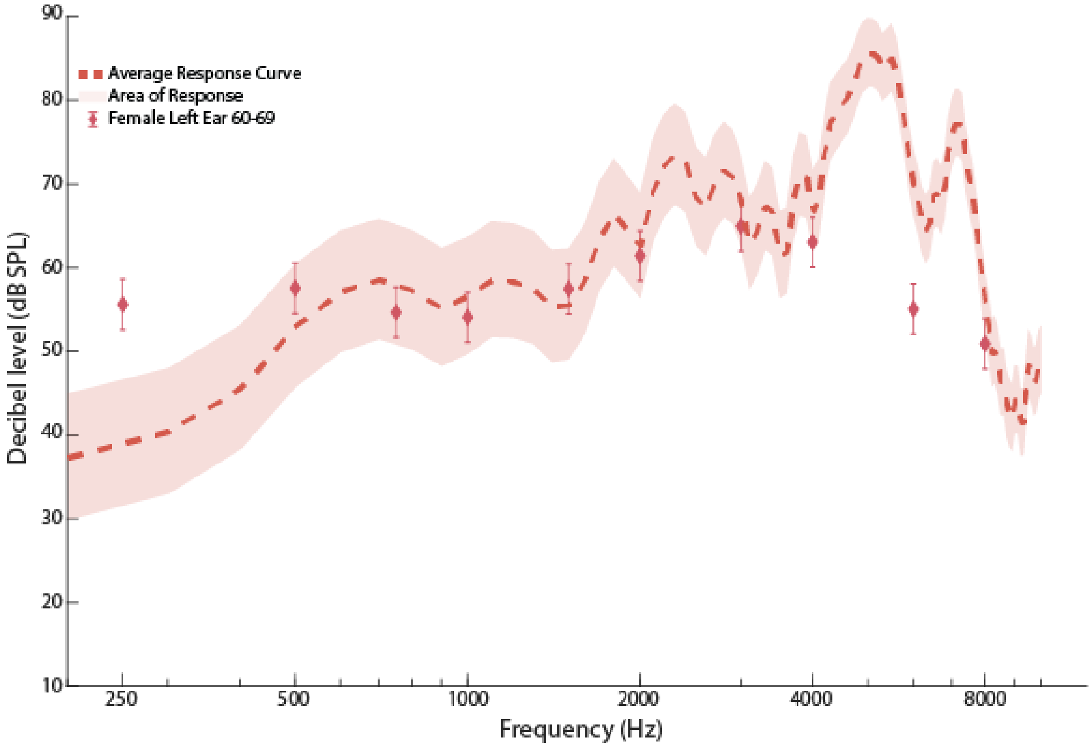

FIG. S43. Shows the G.R.A.S KEMAR 65 db SPL ISTS input Response (dB SPL) Curve (Right Ear) with Female 60-69 Left Ear Targets (dB SPL).

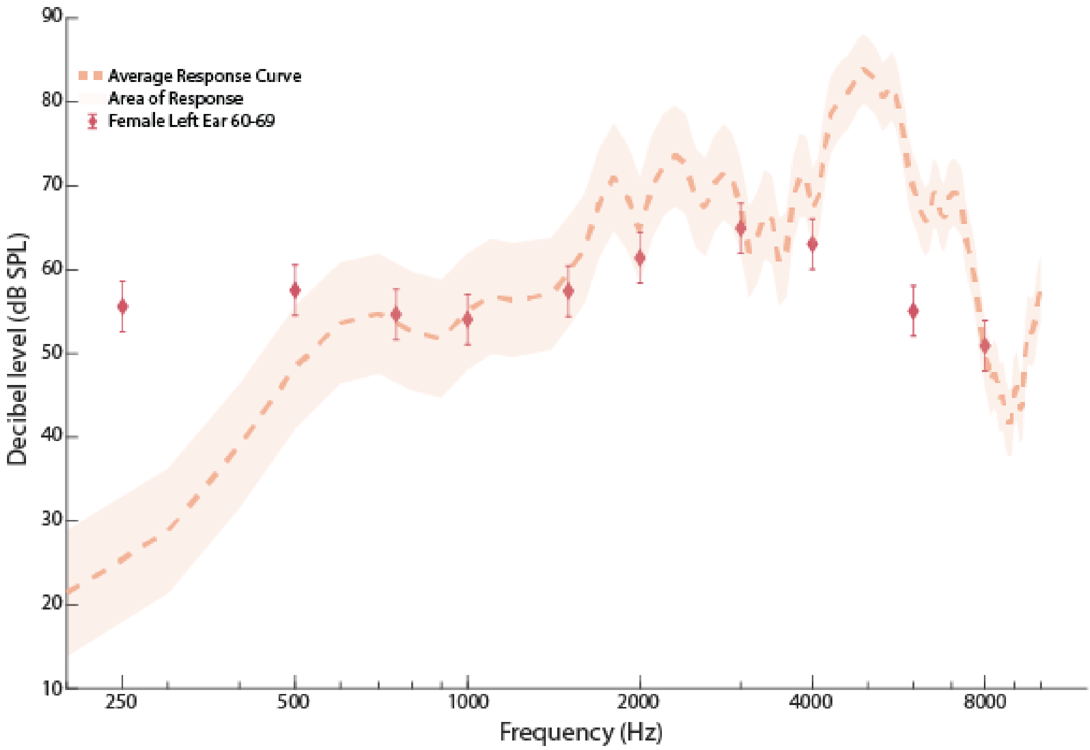

FIG. S44. Shows the G.R.A.S KEMAR 65 db SPL ISTS input Response (dB SPL) Curve (Left Ear) with Female 60-69 Left Ear Targets (dB SPL).

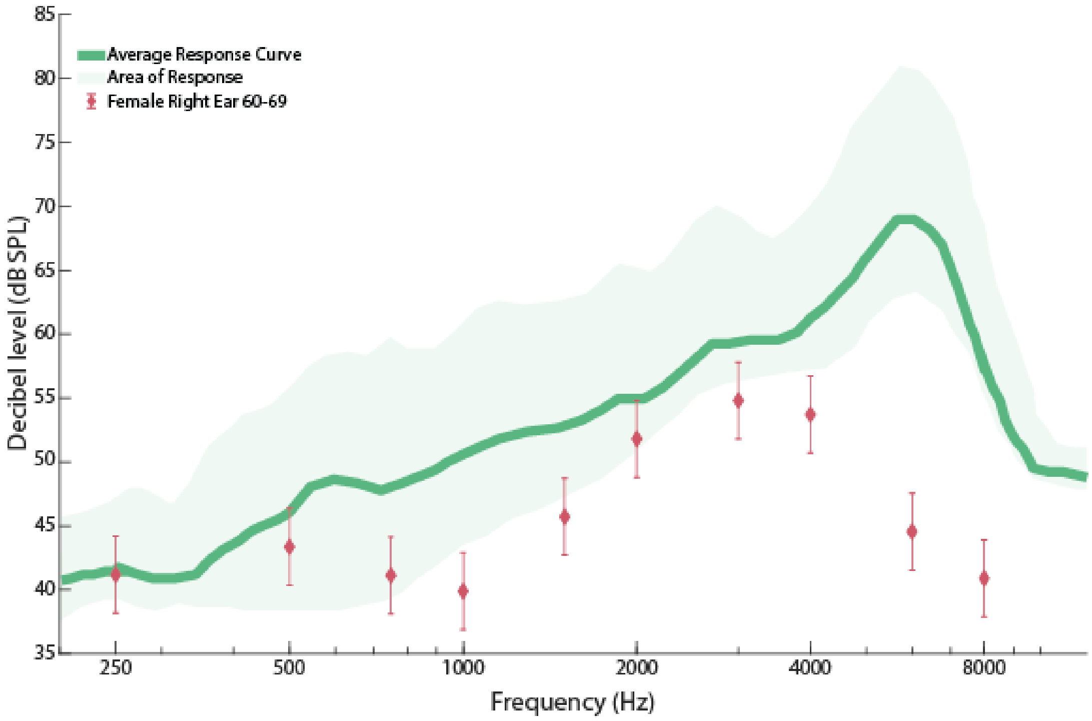

FIG. S45. Shows the ISTS 55 dB SPL Response (dB SPL) Curve with Female 60-69 Right Ear Targets (dB SPL).

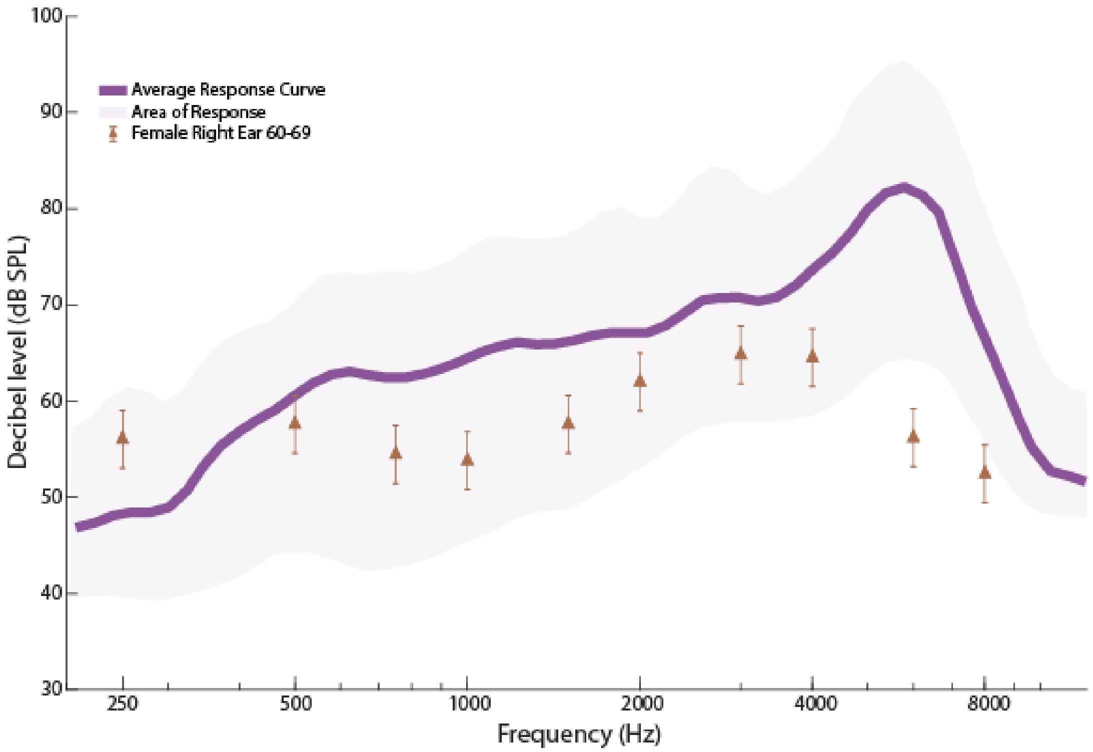

FIG. S46. Shows the ISTS 65 dB SPL Response (dB SPL) Curve with Female 60-69 Right Ear Targets (dB SPL).

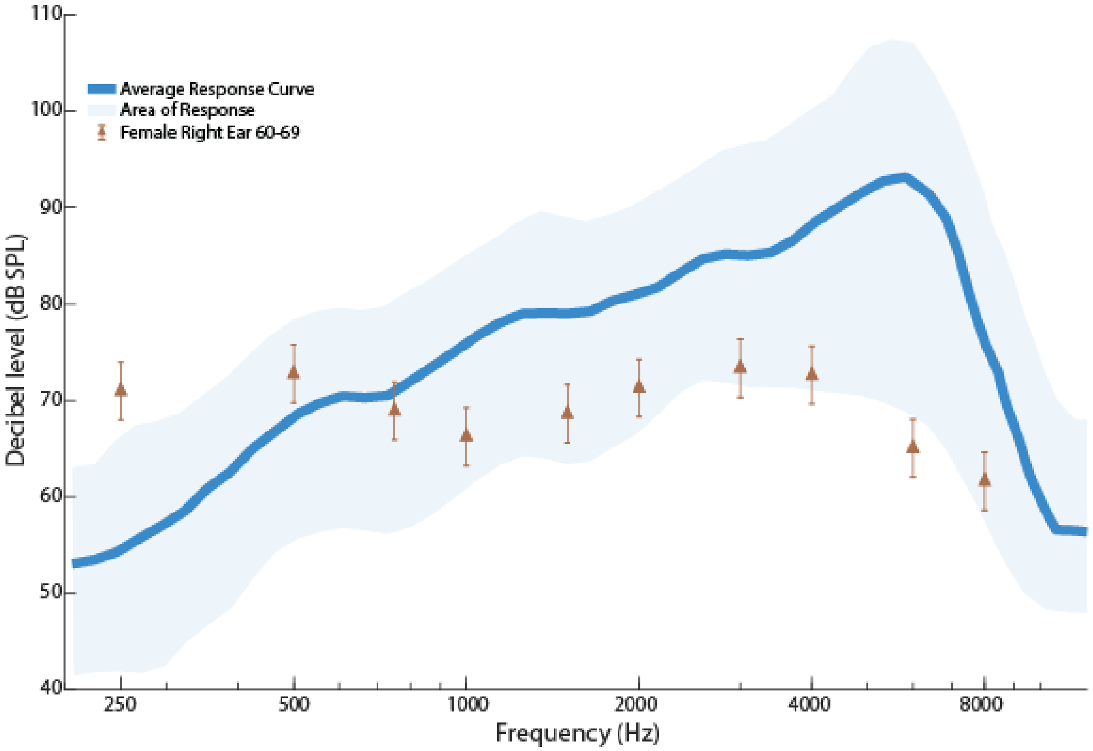

FIG. S47. Shows the ISTS 80 dB SPL Response (dB SPL) Curve with Female 60-69 Right Ear Targets (dB SPL).

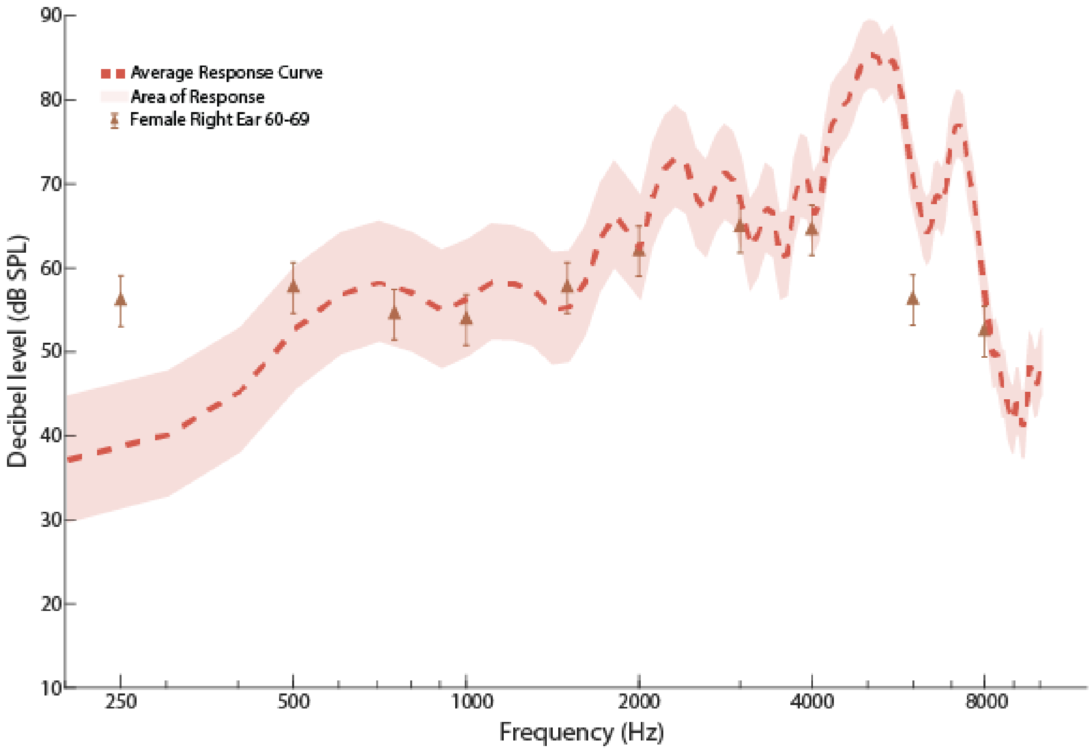

FIG. S48. Shows the G.R.A.S KEMAR 65 db SPL ISTS input Response (dB SPL) Curve (Right Ear) with Female 60-69 Right Ear Targets (dB SPL).

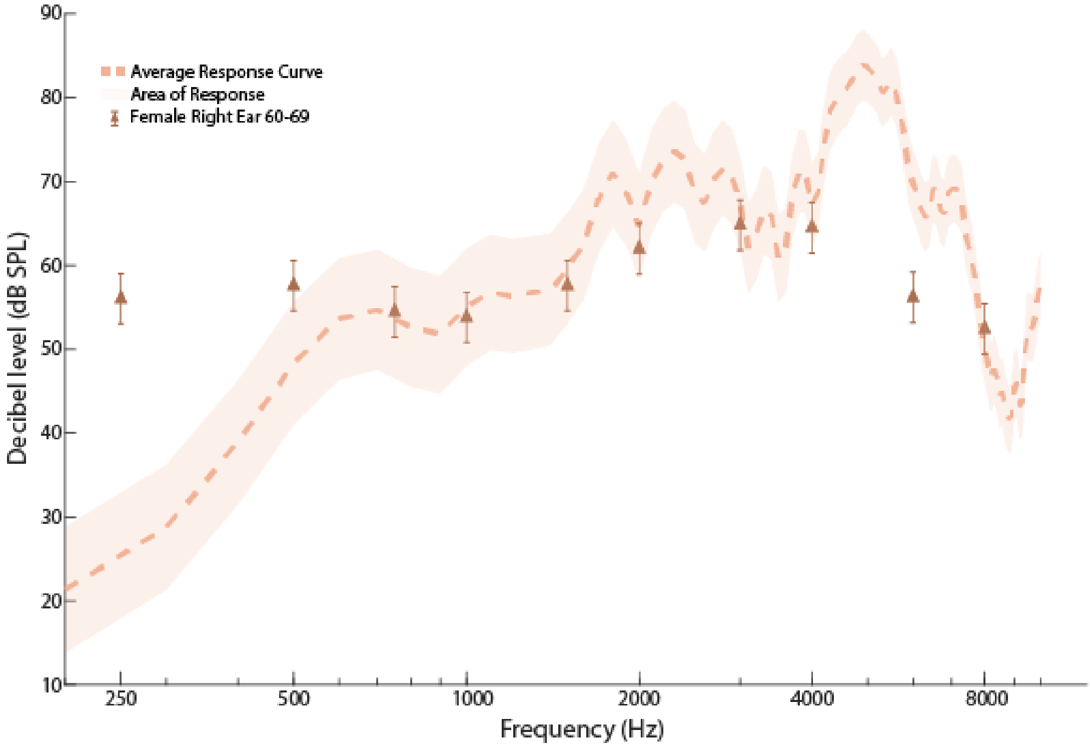

FIG. S49. Shows the G.R.A.S KEMAR 65 db SPL ISTS input Response (dB SPL) Curve (Left Ear) with Female 60-69 Right Ear Targets (dB SPL).

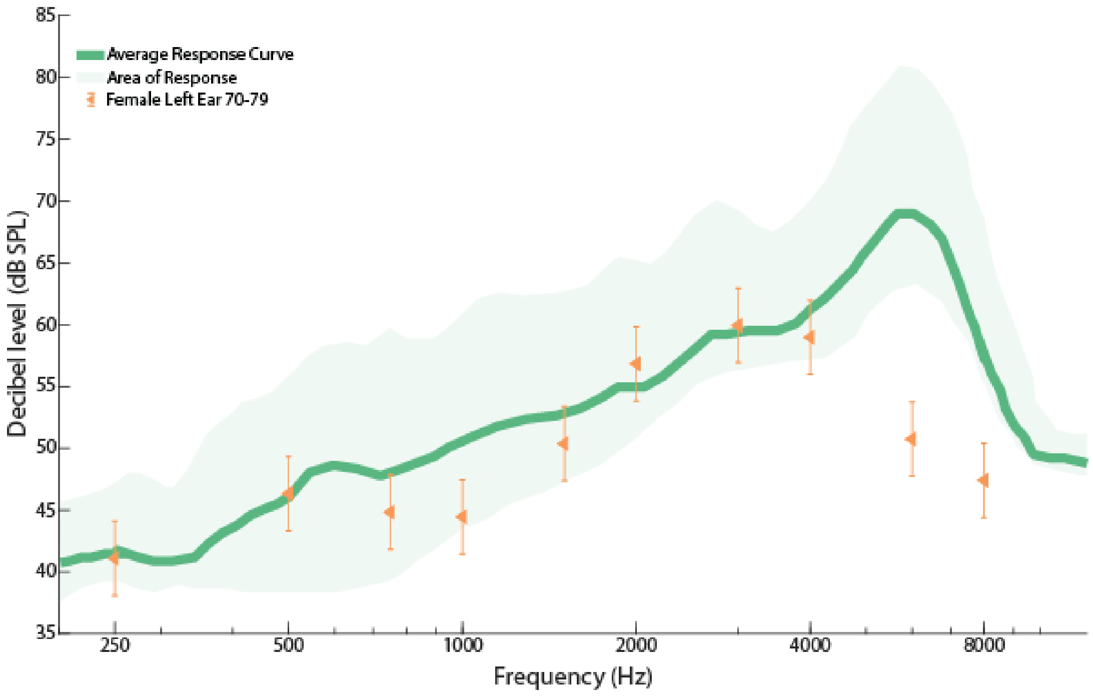

FIG. S50. Shows the ISTS 55 dB SPL Response (dB SPL) Curve with Female 70-79 Left Ear Targets (dB SPL).

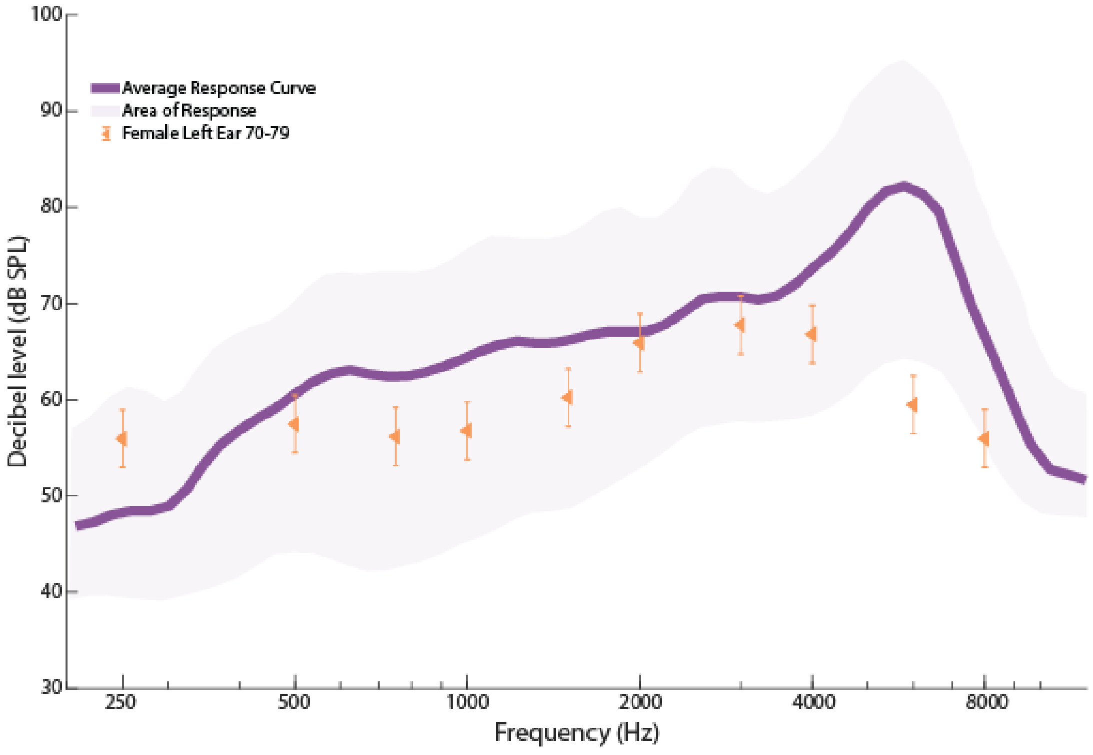

FIG. S51. Shows the ISTS 65 dB SPL Response (dB SPL) Curve with Female 70-79 Left Ear Targets (dB SPL).

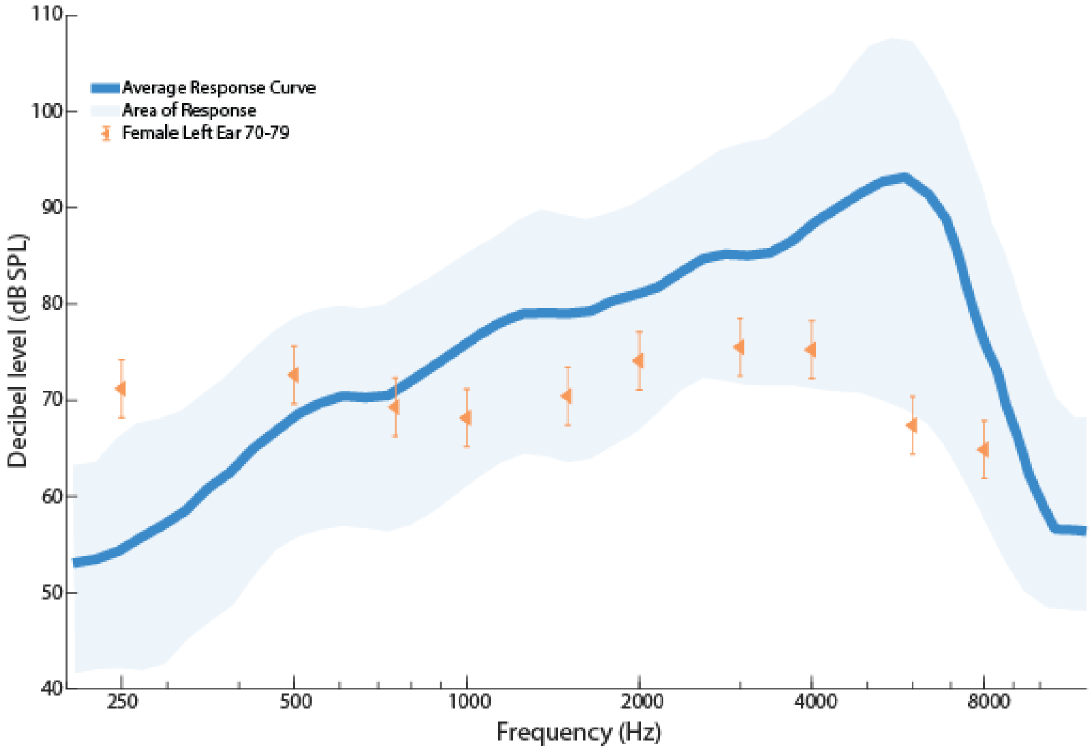

FIG. S52. Shows the ISTS 80 dB SPL Response (dB SPL) Curve with Female 70-79 Left Ear Targets (dB SPL).

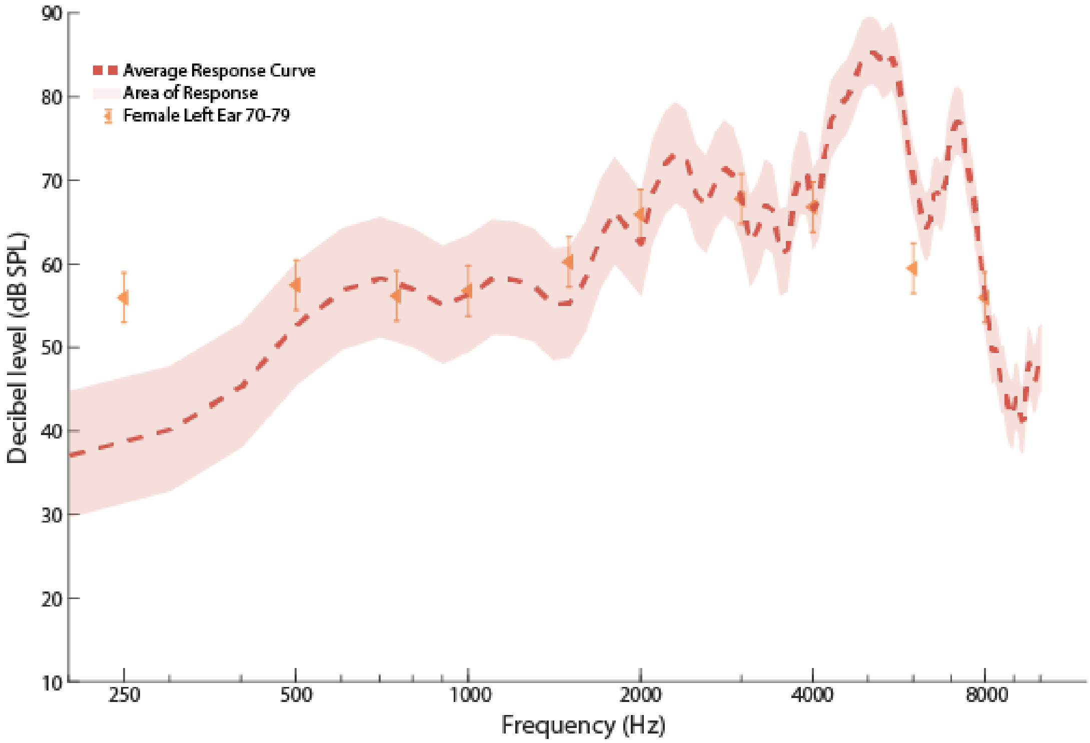

FIG. S53. Shows the G.R.A.S KEMAR 65 db SPL ISTS input Response (dB SPL) Curve (Right Ear) with Female 70-79 Left Ear Targets (dB SPL).

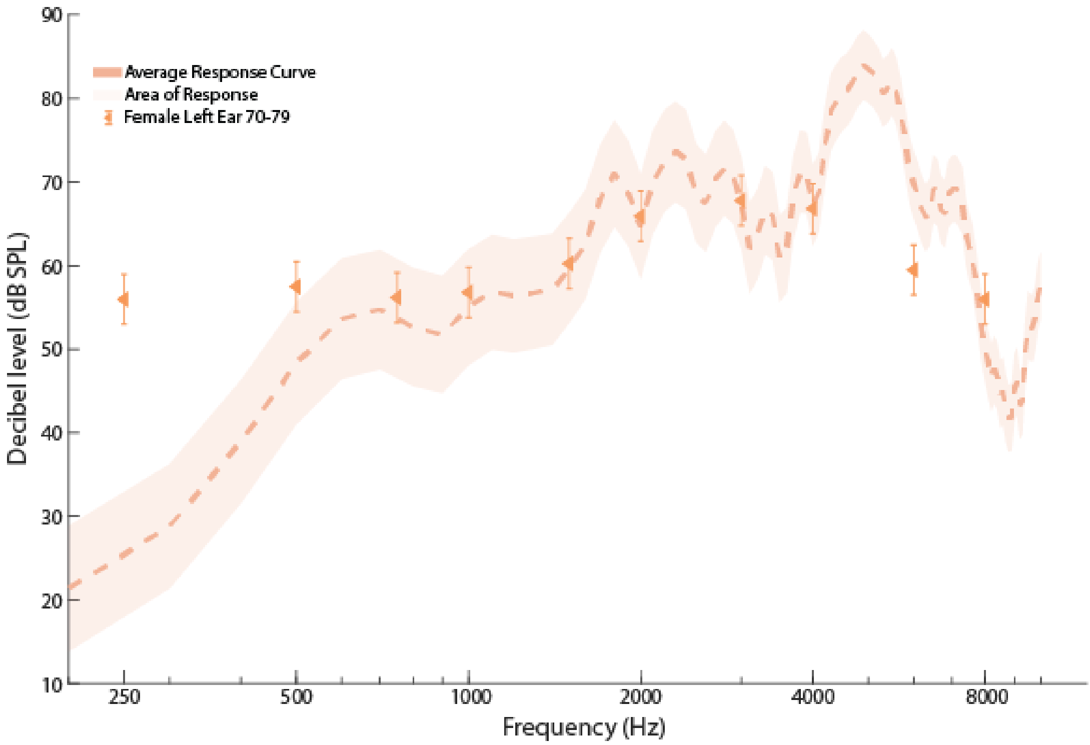

FIG. S54. Shows the G.R.A.S KEMAR 65 db SPL ISTS input Response (dB SPL) Curve (Left Ear) with Female 70-79 Left Ear Targets (dB SPL).

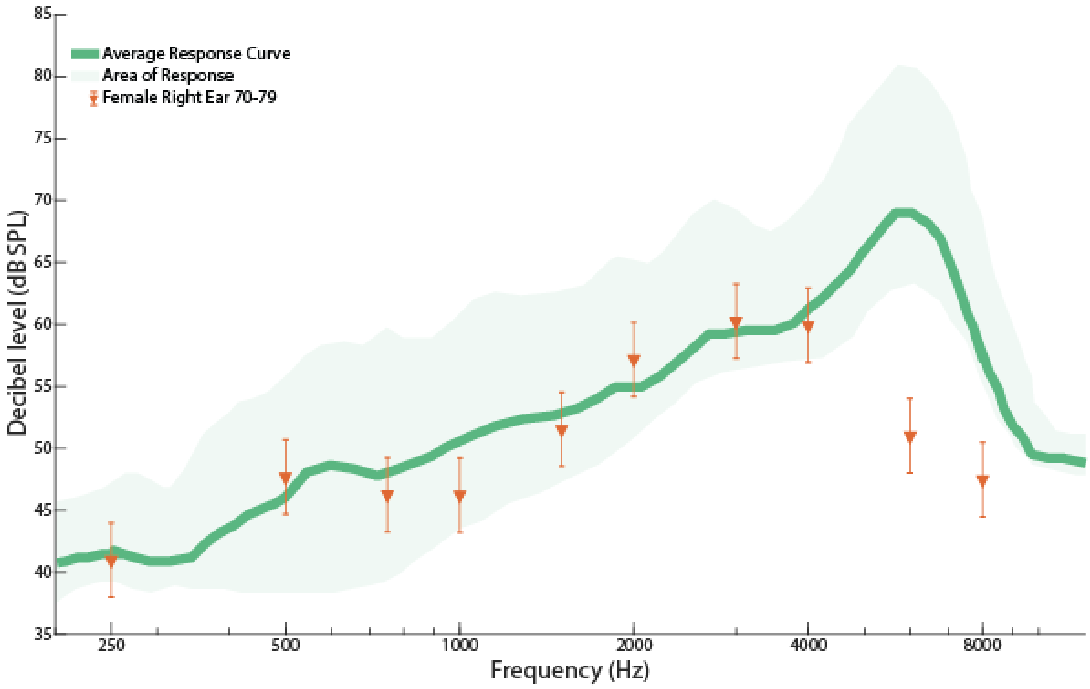

FIG. S55. Shows the ISTS 55 dB SPL Response (dB SPL) Curve with Male 70-79 Right Ear Targets (dB SPL).

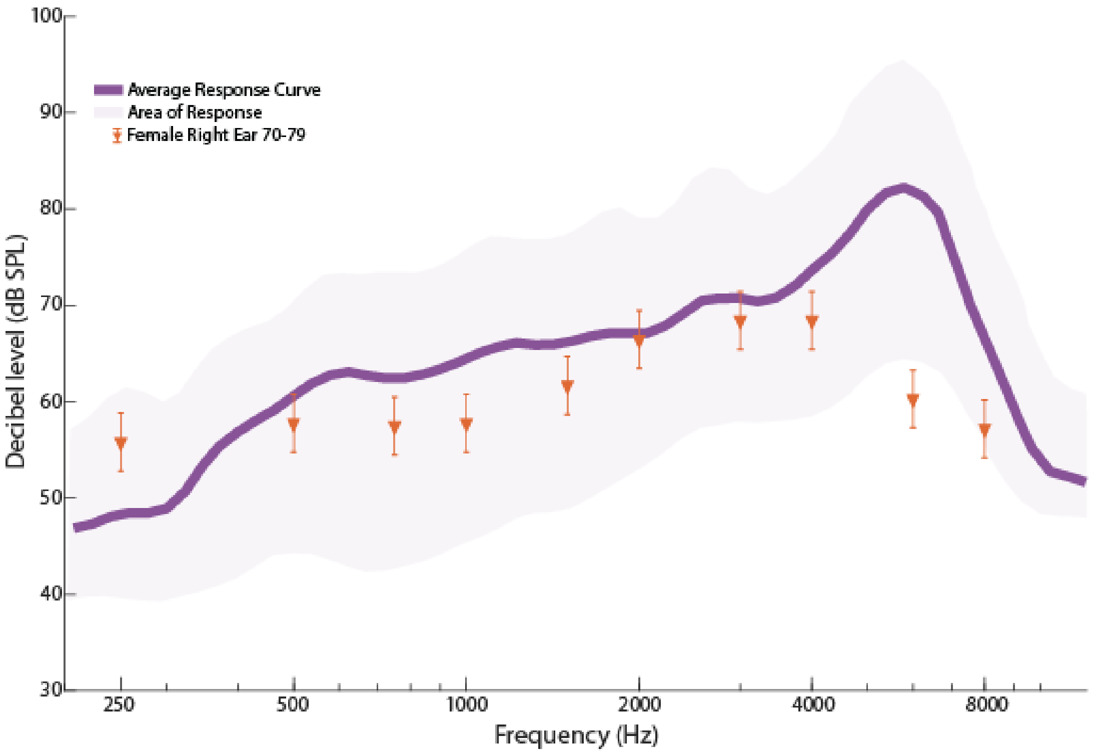

FIG. S56. Shows the ISTS 65 dB SPL Response (dB SPL) Curve with Female 70-79 Right Ear Targets (dB SPL).

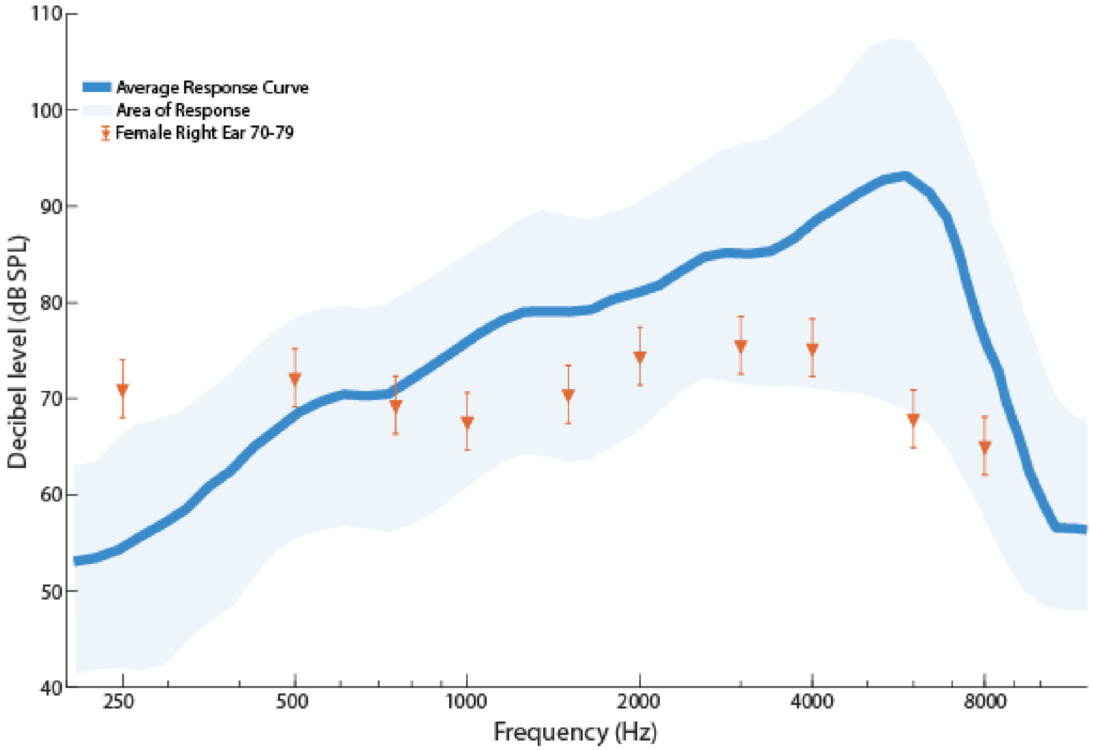

FIG. S57. Shows the ISTS 80 dB SPL Response (dB SPL) Curve with Female 70-79 Right Ear Targets (dB SPL).

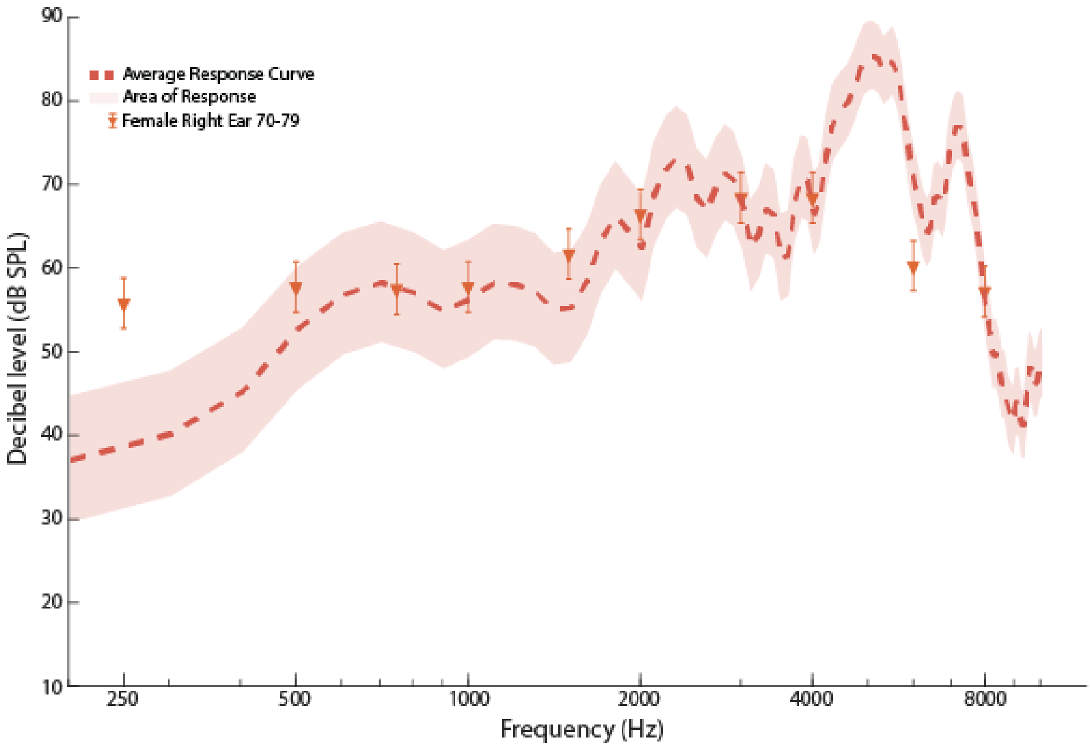

FIG. S58. Shows the G.R.A.S KEMAR 65 db SPL ISTS input Response (dB SPL) Curve (Right Ear) with Female 70-79 Right Ear Targets (dB SPL).

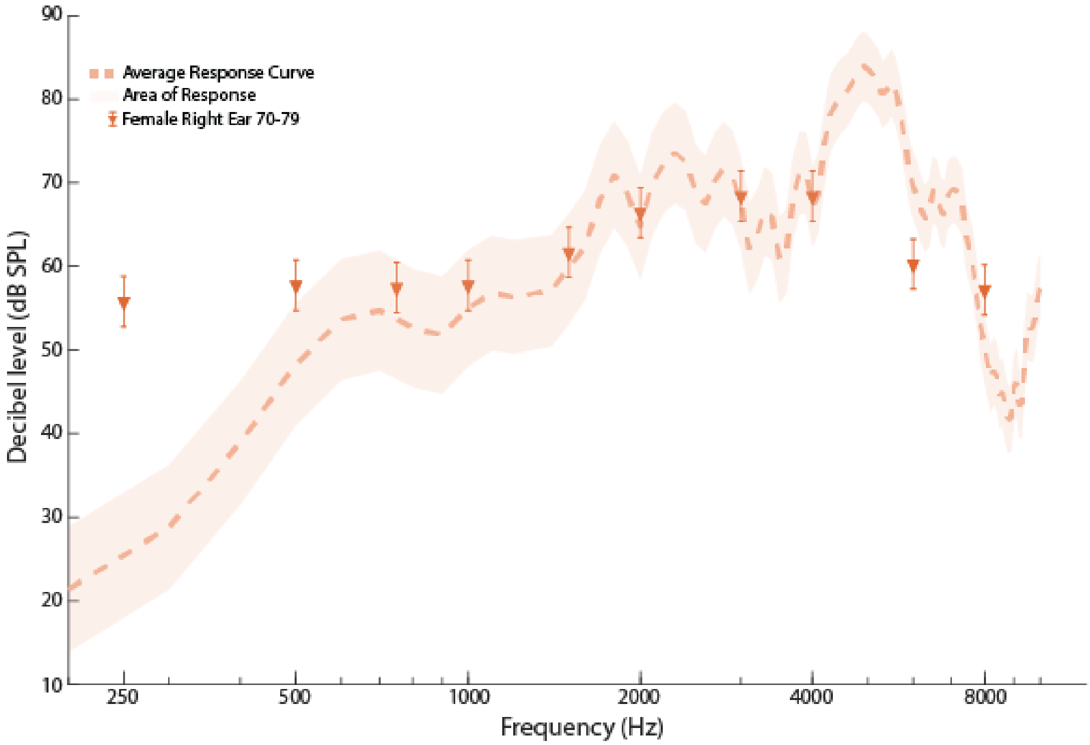

FIG. S59. Shows the G.R.A.S KEMAR 65 db SPL ISTS input Response (dB SPL) Curve (Left Ear) with Female 70-79 Right Ear Targets (dB SPL).

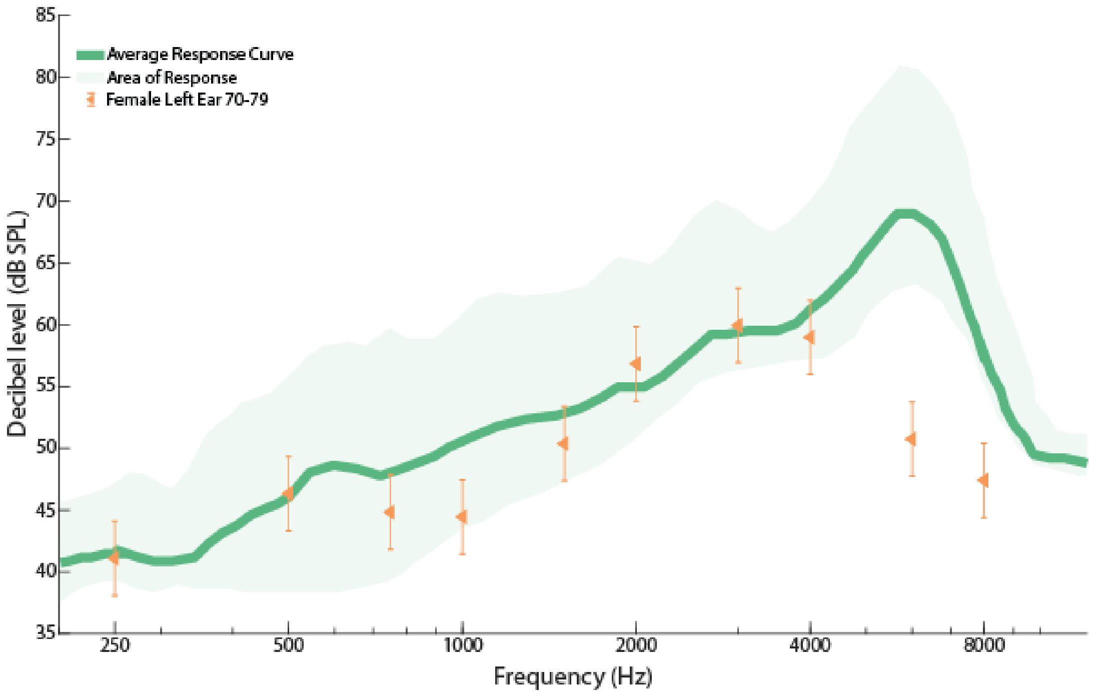

FIG. S60. Shows the ISTS 55 dB SPL Response (dB SPL) Curve with Female 70-79 Left Ear Targets (dB SPL).

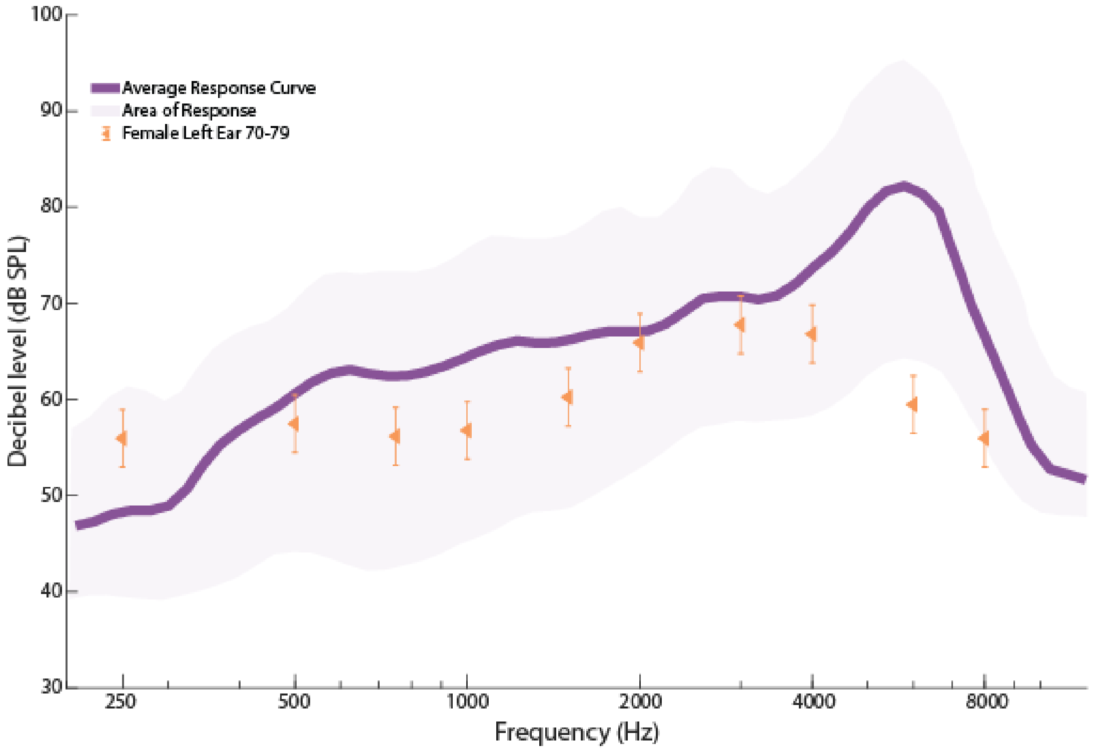

FIG. S61. Shows the ISTS 65 dB SPL Response (dB SPL) Curve with Female 70-79 Left Ear Targets (dB SPL).

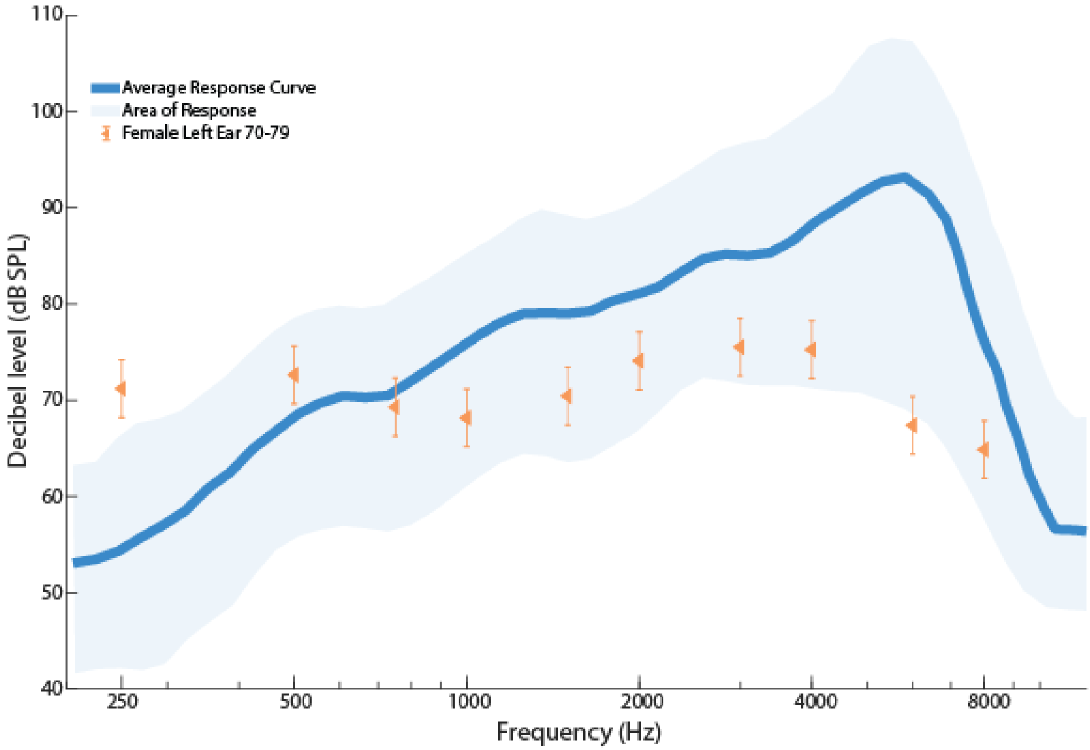

FIG. S62. Shows the ISTS 80 dB SPL Response (dB SPL) Curve with Female 70-79 Left Ear Targets (dB SPL).

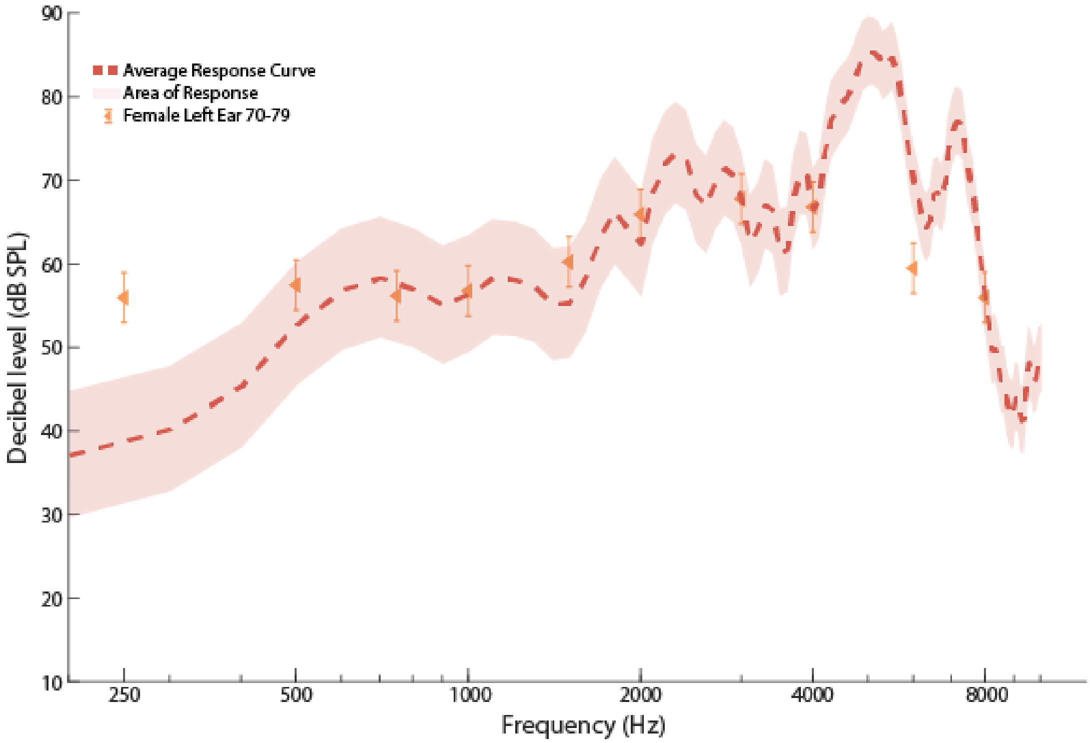

FIG. S63. Shows the G.R.A.S KEMAR 65 db SPL ISTS input Response (dB SPL) Curve (Right Ear) with Female 70-79 Left Ear Targets (dB SPL).

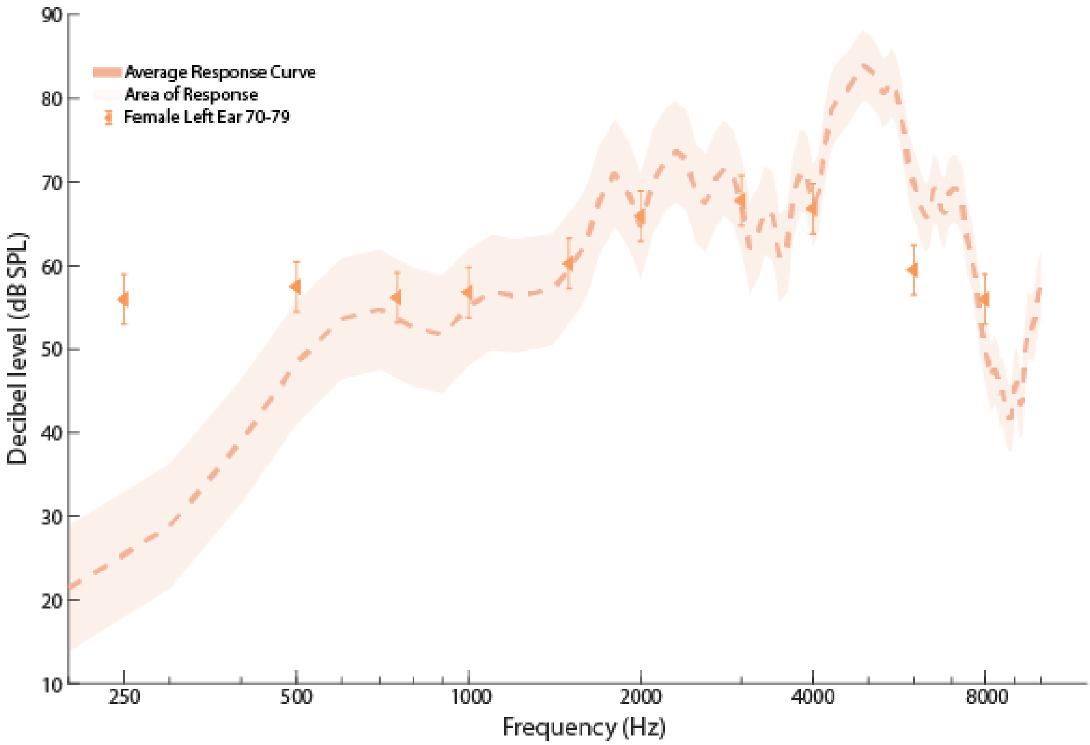

FIG. S64. Shows the G.R.A.S KEMAR 65 db SPL ISTS input Response (dB SPL) Curve (Left Ear) with Female 70-79 Left Ear Targets (dB SPL).

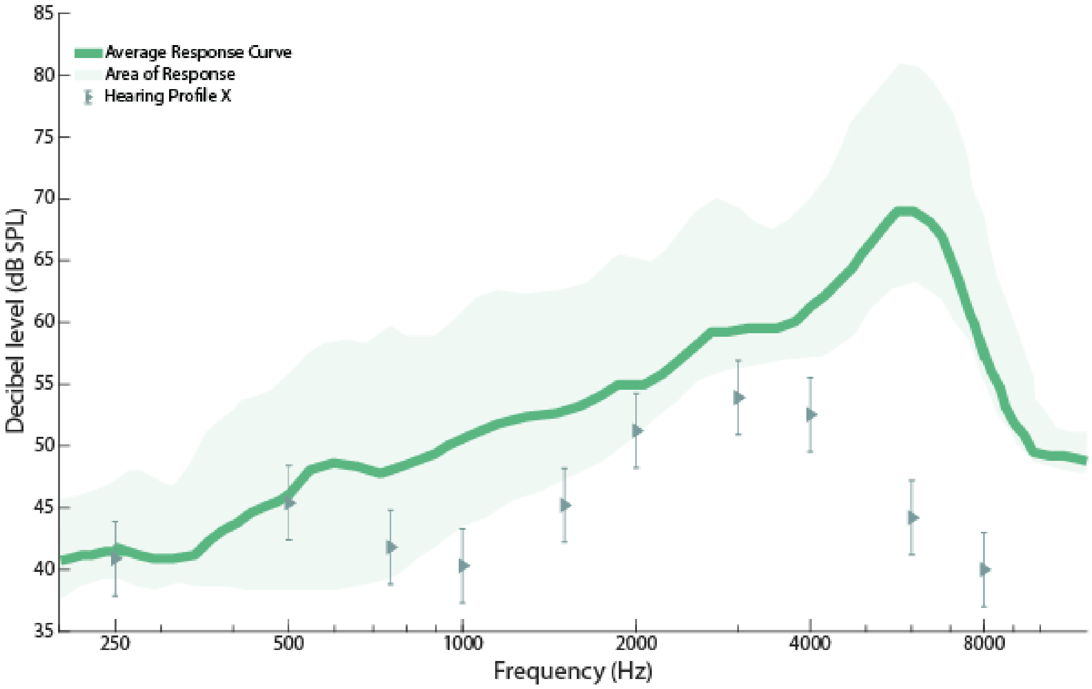

FIG. S65. Shows the ISTS 55 dB SPL Response (dB SPL) Curve with Hearing Profile X Targets (dB SPL).

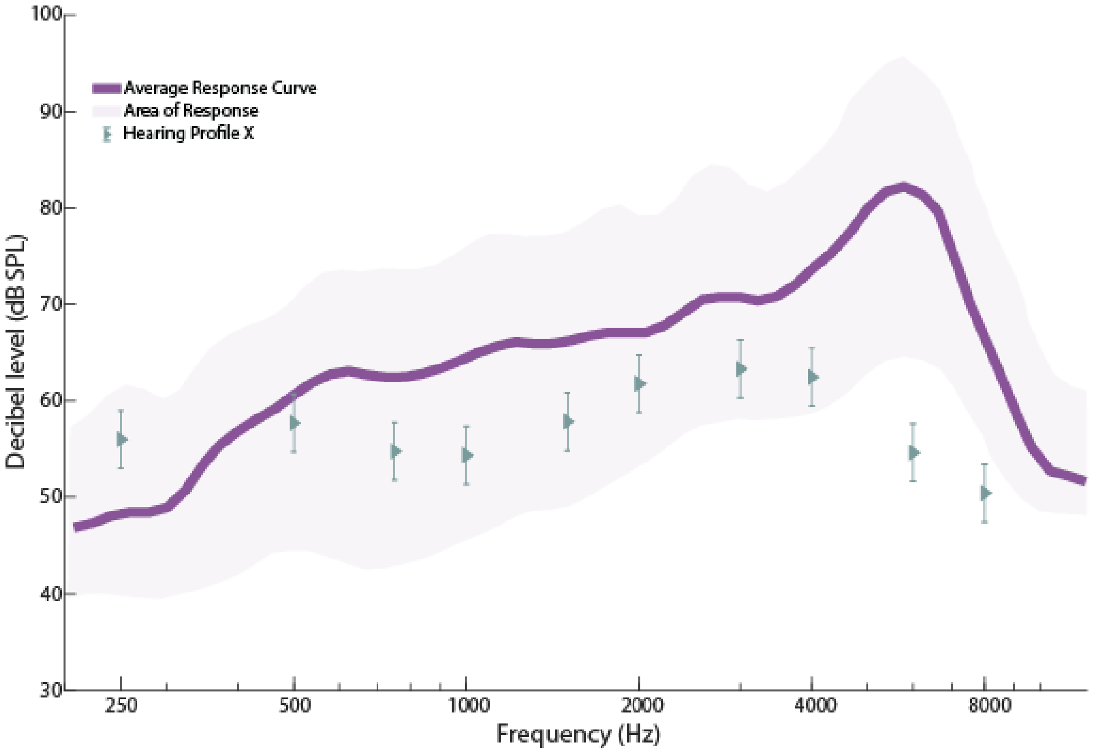

FIG. S66. Shows the ISTS 65 dB SPL Response (dB SPL) Curve with Hearing Profile X Targets (dB SPL).

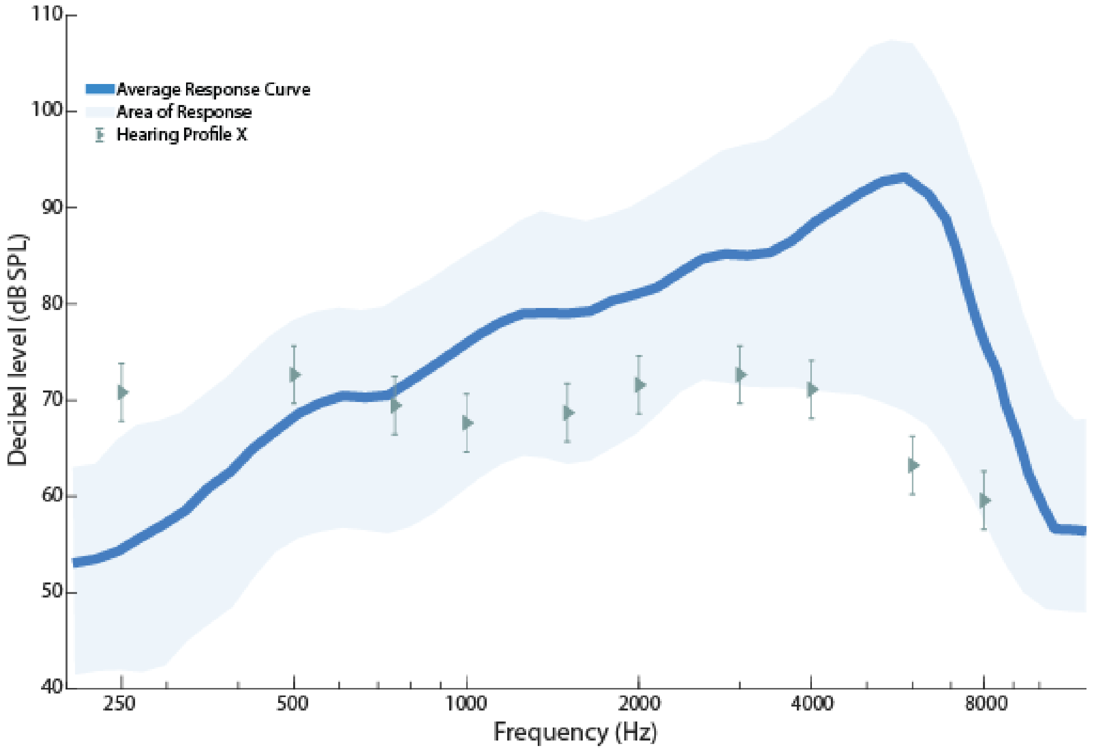

FIG. S67. Shows the ISTS 80 dB SPL Response (dB SPL) Curve with Hearing Profile X Targets (dB SPL).

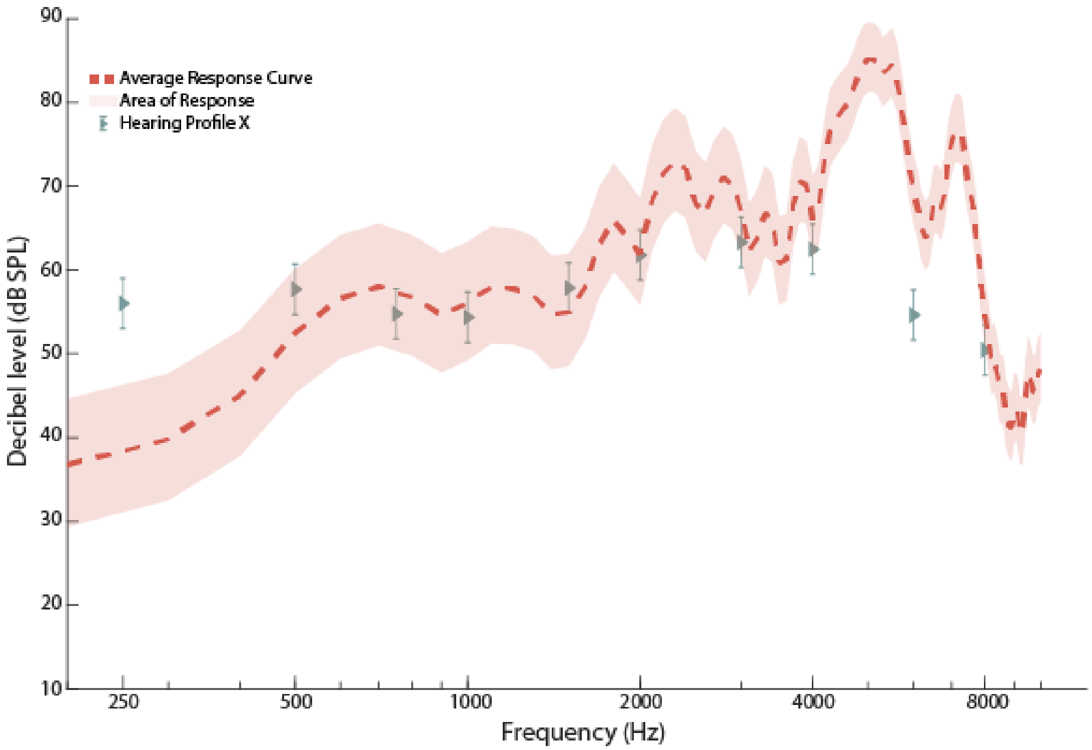

FIG. S68. Shows the G.R.A.S KEMAR 65 db SPL ISTS input Response (dB SPL) Curve (Right Ear) with Hearing Profile X Targets (dB SPL).

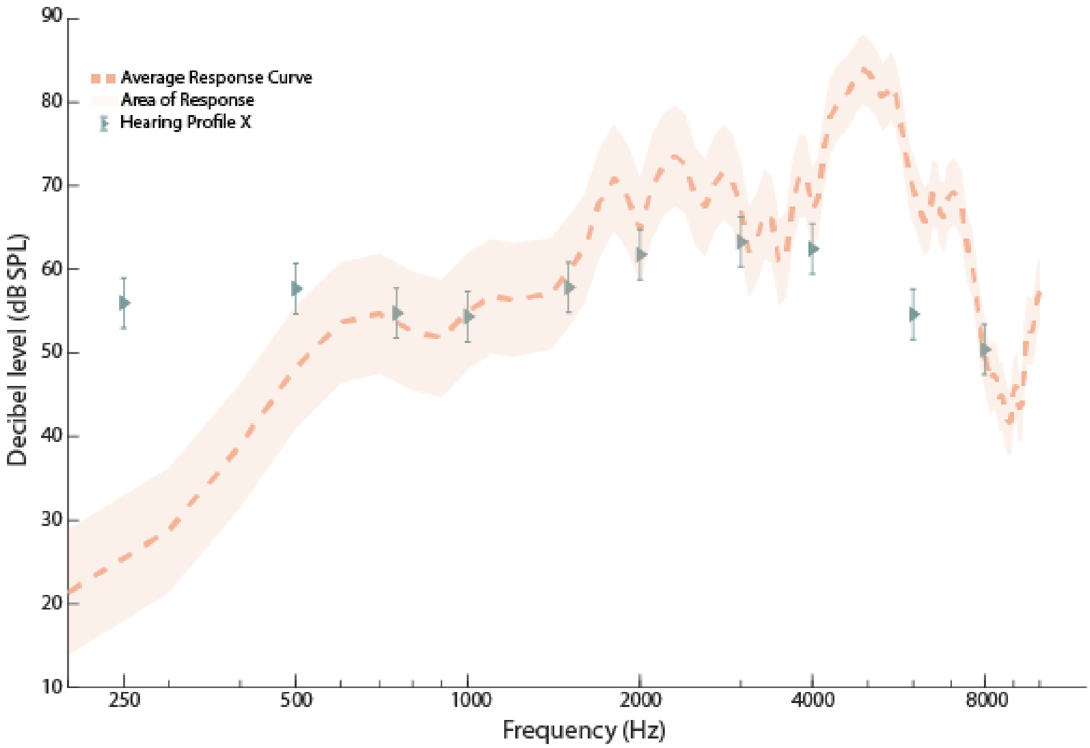

FIG. S69. Shows the G.R.A.S KEMAR 65 db SPL ISTS input Response (dB SPL) Curve (Left Ear) with Hearing Profile X Targets (dB SPL).

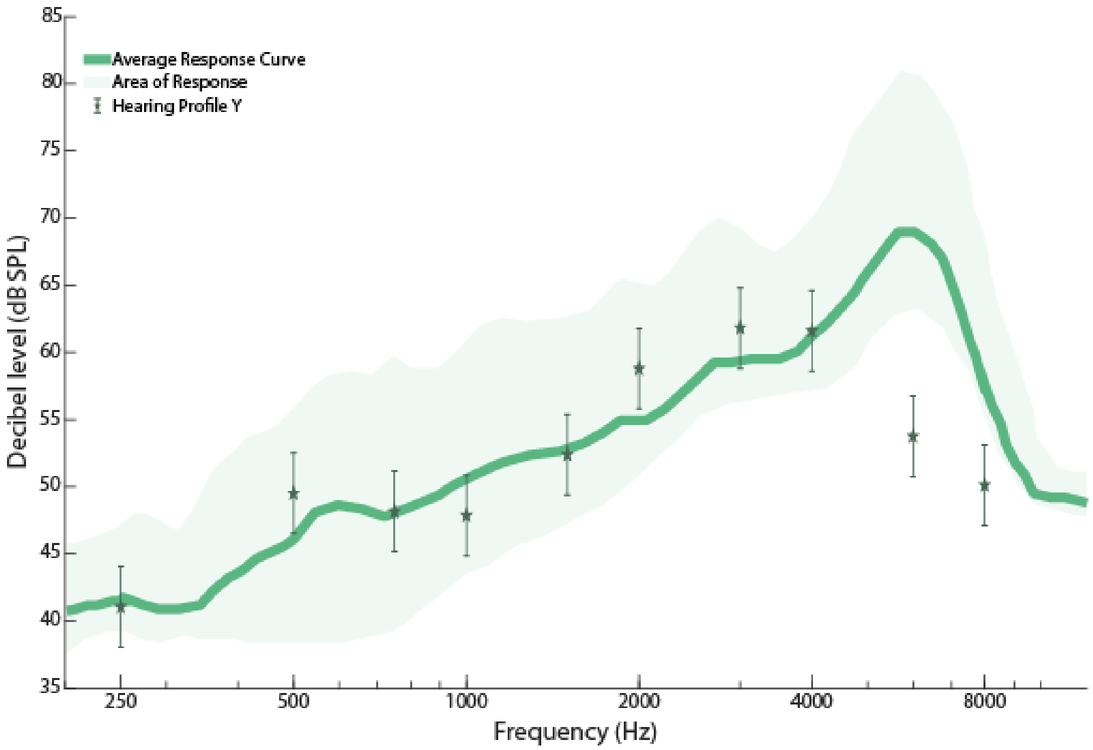

FIG. S70. Shows the ISTS 55 dB SPL Response (dB SPL) Curve with Hearing Profile Y Targets (dB SPL).

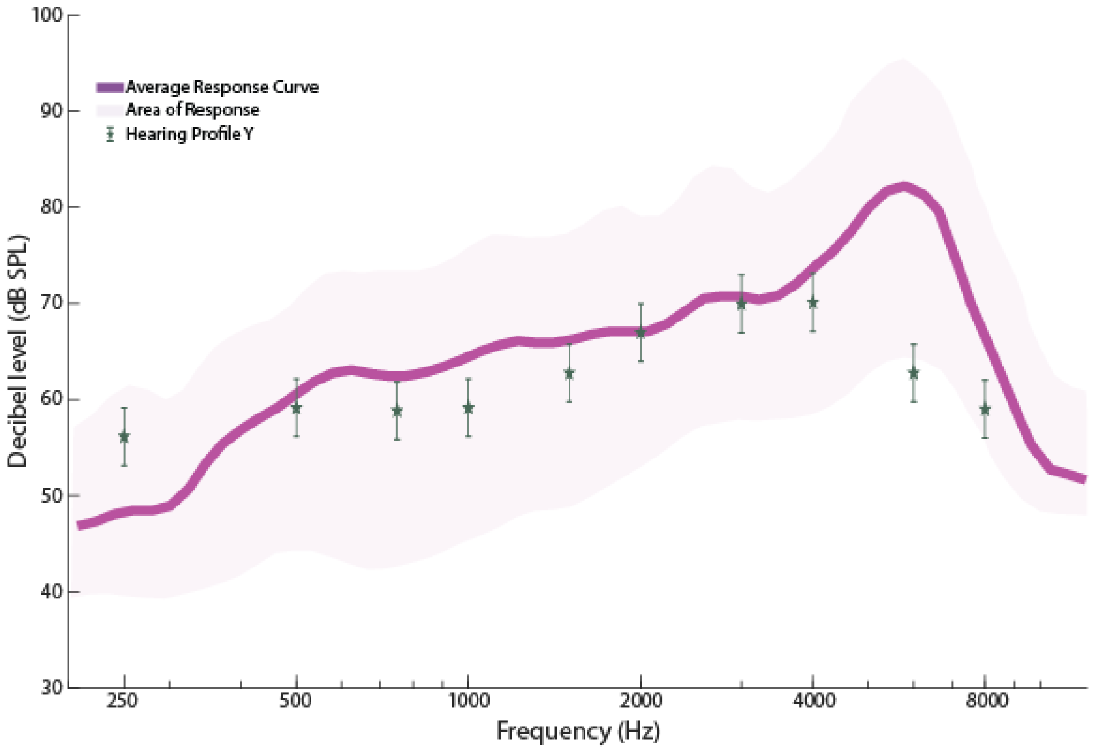

FIG. S71. Shows the ISTS 65 dB SPL Response (dB SPL) Curve with Hearing Profile Y Targets (dB SPL).

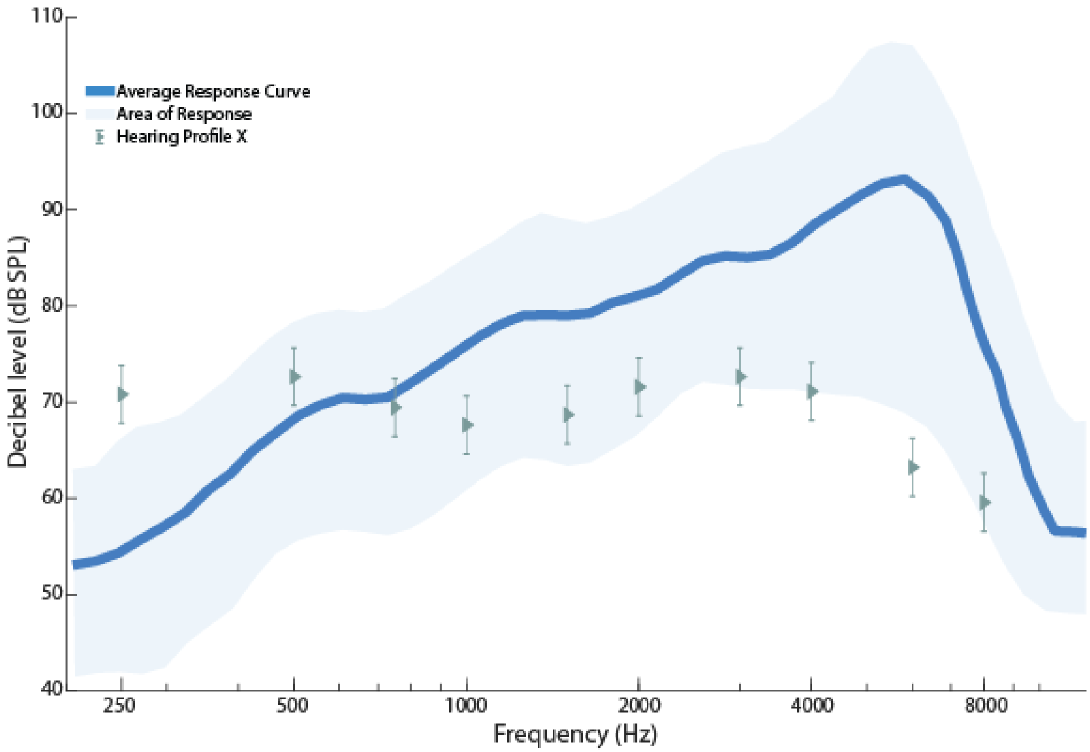

FIG. S72. Shows the ISTS 80 dB SPL Response (dB SPL) Curve with Hearing Profile Y Targets (dB SPL).

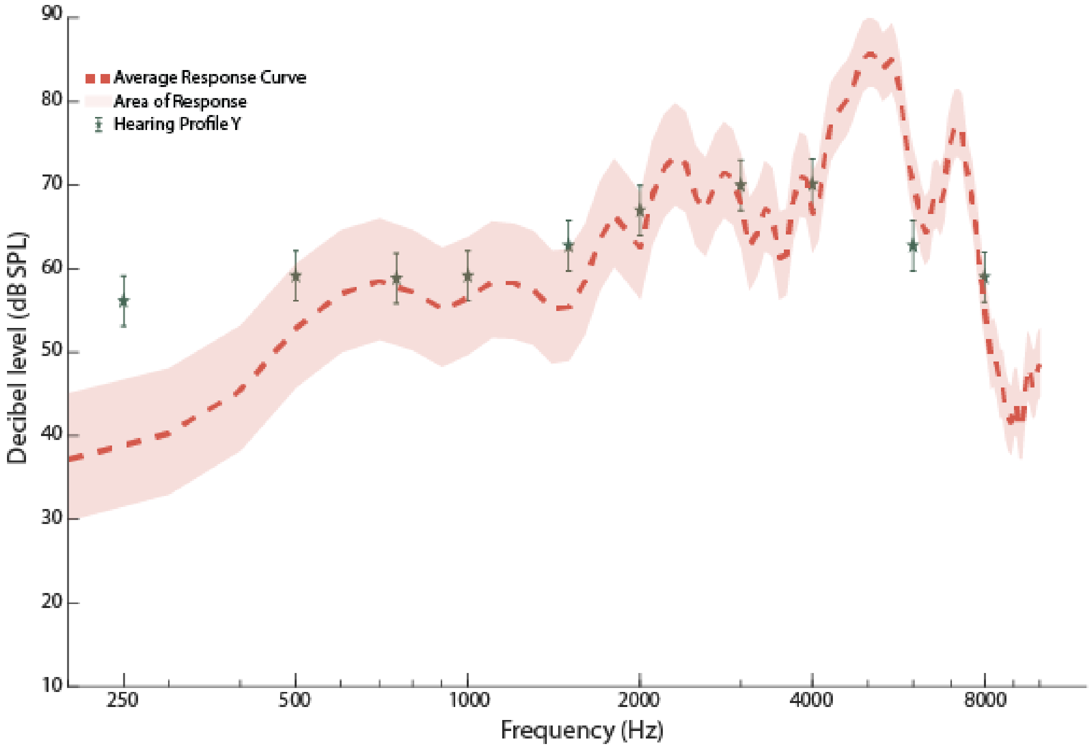

FIG. S73. Shows the G.R.A.S KEMAR 65 db SPL ISTS input Response (dB SPL) Curve (Right Ear) with Hearing Profile Y Targets (dB SPL).

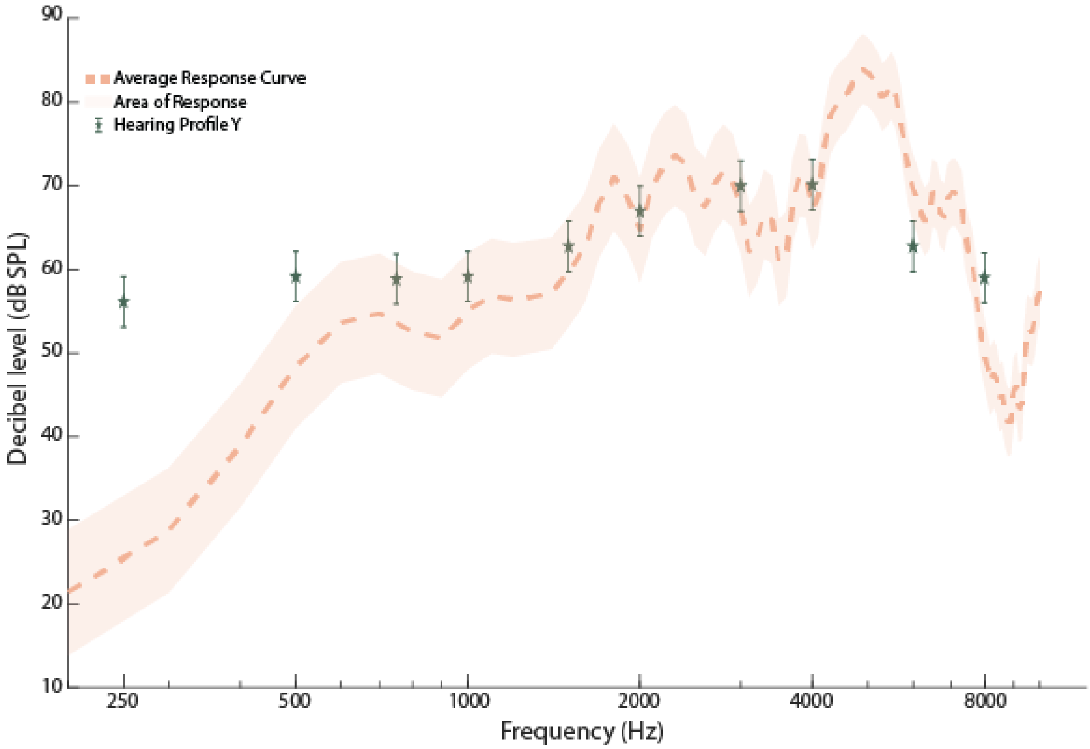

FIG. S74. Shows the G.R.A.S KEMAR 65 db SPL ISTS input Response (dB SPL) Curve (Left Ear) with Hearing Profile Y Targets (dB SPL).

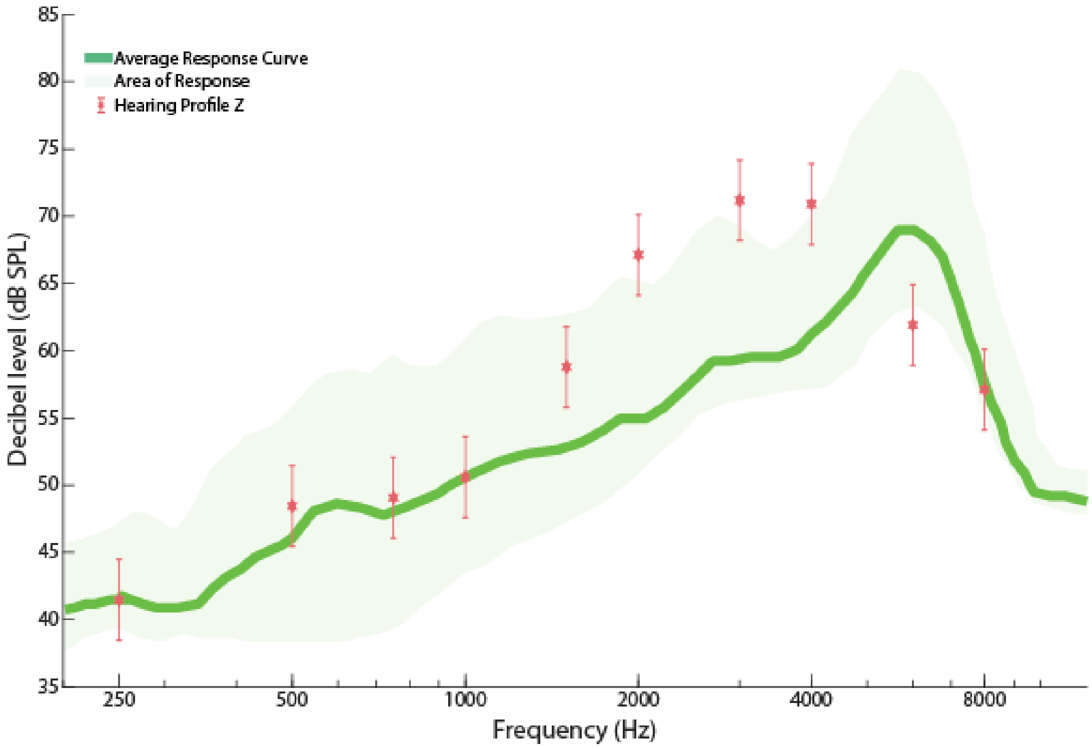

FIG. S75. Shows the ISTS 55 dB SPL Response (dB SPL) Curve with Hearing Profile Z Targets (dB SPL).

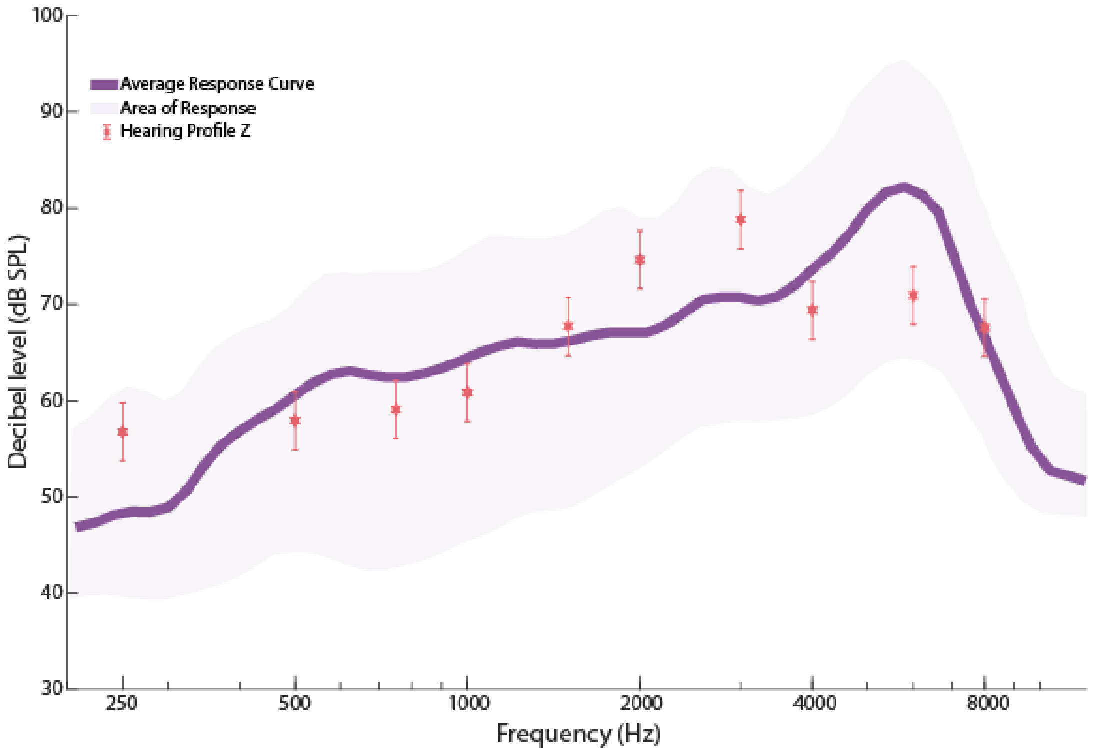

FIG. S76. Shows the ISTS 65 dB SPL Response (dB SPL) Curve with Hearing Profile Z Targets (dB SPL).

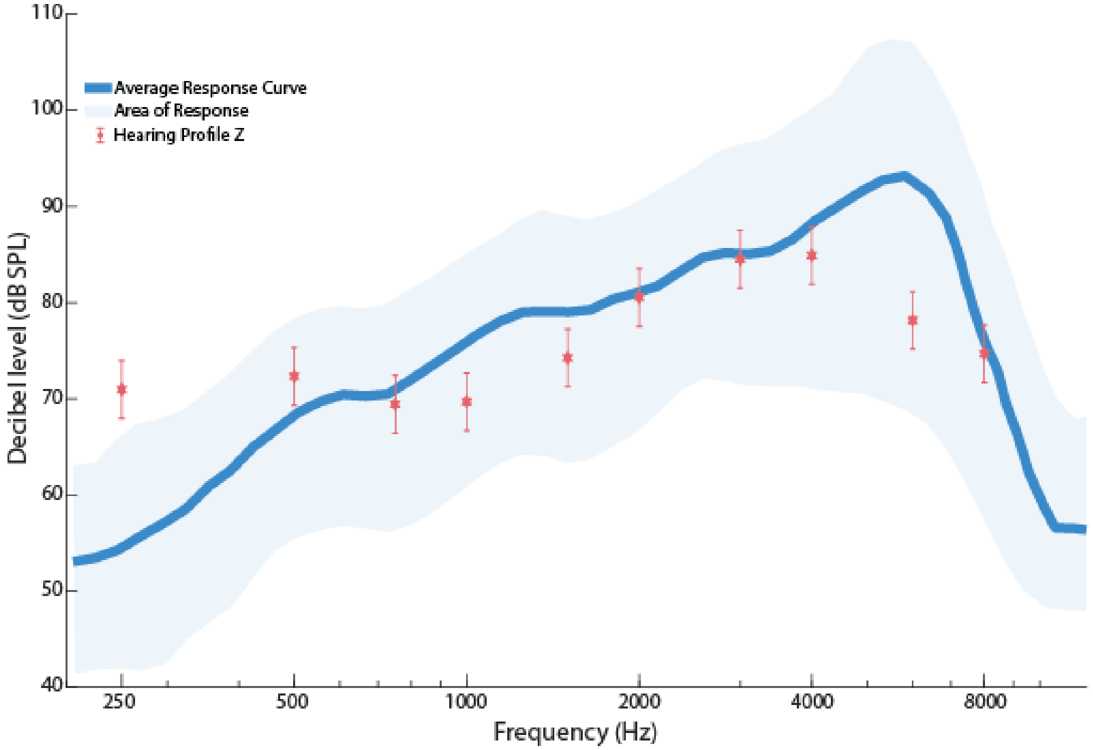

FIG. S77. Shows the ISTS 80 dB SPL Response (dB SPL) Curve with Hearing Profile Z Targets (dB SPL).

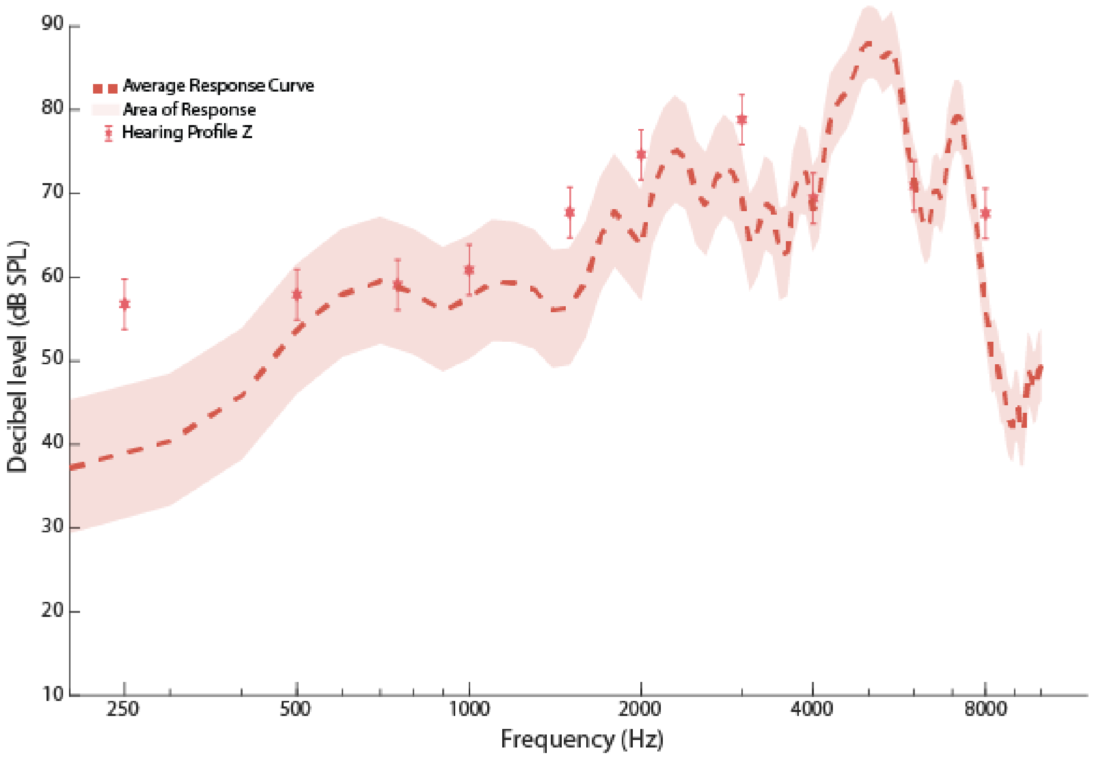

FIG. S78. Shows the G.R.A.S KEMAR 65 db SPL ISTS input Response (dB SPL) Curve (Right Ear) with Hearing Profile Z Targets (dB SPL).

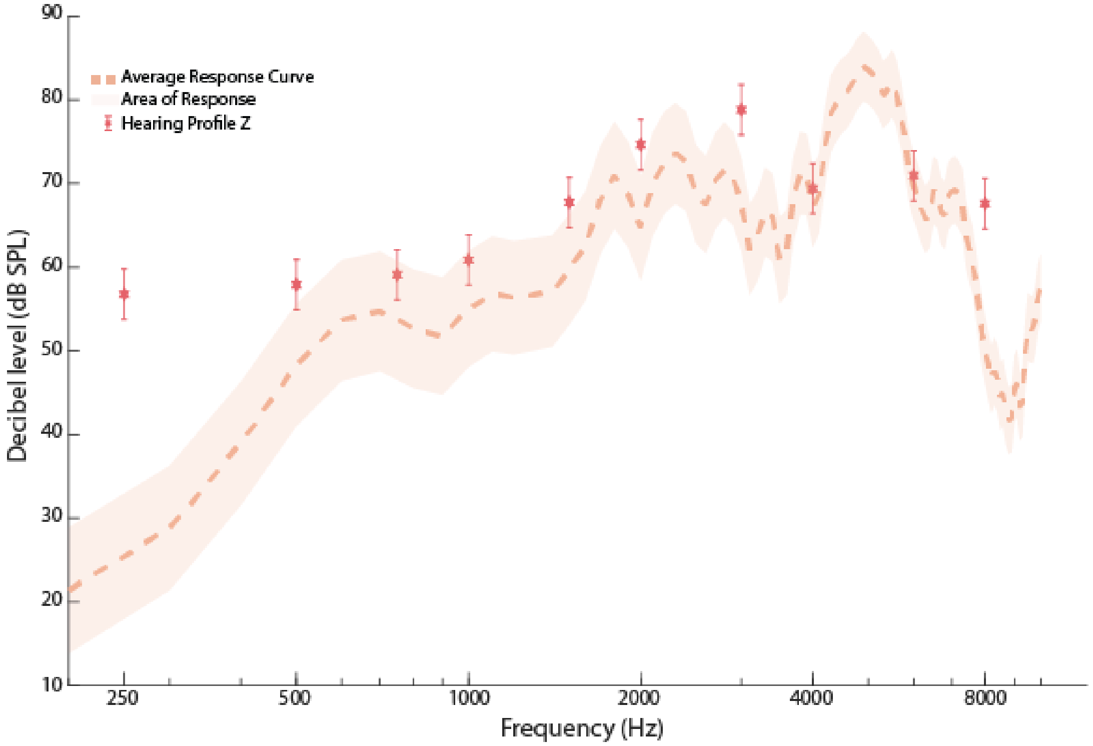

FIG. S79. Shows the G.R.A.S KEMAR 65 db SPL ISTS input Response (dB SPL) Curve (Left Ear) with Hearing Profile Z Targets (dB SPL).

Frequencies (Hz)	Response (dB SPL)	Targets (dB SPL)	Meets Strict Criteria	Meets Loose Criteria
250	41	40	Yes	Yes
500	46	43.56	Yes	Yes
750	48	43.52	Yes	Yes
1000	51	44.67	No	Yes
1500	53	51.3	Yes	Yes
2000	55	58.5	Yes	Yes
3000	59	64.3	No	Yes
4000	61	63.7	Yes	Yes
6000	69	54.4	No	No
8000	57	50.29	No	Yes

TABLE I. Shows the individual Targets (dB SPL) for Male Left Ear 60-69 ISTS 55 dB SPL for each Frequencies (Hz), and whether the device meets them under two criteria

.

Frequencies (Hz)	Response (dB SPL)	Targets (dB SPL)	Meets Strict Criteria	Meets Loose Criteria
250	48	55	No	Yes
500	61	57	Yes	Yes
750	62	56	No	Yes
1000	65	57	No	Yes
1500	66	61.6	Yes	Yes
2000	67	67.4	Yes	Yes
3000	71	71.86	Yes	Yes
4000	74	72	Yes	Yes
6000	81	64	No	No
8000	67	60.2	No	Yes

TABLE II. Shows the individual Targets (dB SPL) for Male Left Ear 60-69 ISTS 65 dB SPL for each Frequencies (Hz), and whether the device meets them under two criteria

.

Frequencies (Hz)	Response (dB SPL)	Targets (dB SPL)	Meets Strict Criteria	Meets Loose Criteria
250	54	71	No	No
500	69	72	Yes	Yes
750	71	69	Yes	Yes
1000	76	66	No	No
1500	79	70	No	Yes
2000	81	74.5	No	Yes
3000	85	77.76	No	Yes
4000	89	77.76	No	No
6000	92	70.5	No	No
8000	76	67.6	No	Yes

TABLE III. Shows the individual Targets (dB SPL) for Male Left Ear 60-69 ISTS 80 dB SPL for each Frequencies (Hz), and whether the device meets them under two criteria

.

Frequencies (Hz)	Response (dB SPL)	Targets (dB SPL)	Meets Strict Criteria	Meets Loose Criteria
250	39	55	No	No
500	53	57	Yes	Yes
750	58	56	Yes	Yes
1000	56	57	Yes	Yes
1500	55	61.6	No	Yes
2000	62	67	Yes	Yes
3000	67	71.86	Yes	Yes
4000	66	71	Yes	Yes
6000	69	64	Yes	Yes
8000	55	60	Yes	Yes

TABLE IV. Shows the individual Targets (dB SPL) for Male Left Ear 60-69 G.R.A.S KEMAR Left Ear ISTS 65 dB SPL for each Frequencies (Hz), and whether the device meets them under two criteria

Frequencies (Hz)	Response (dB SPL)	Targets (dB SPL)	Meets Strict Criteria	Meets Loose Criteria
250	25	55	No	No
500	48	57	No	Yes
750	54	56	Yes	Yes
1000	55	57	Yes	Yes
1500	60	61.6	Yes	Yes
2000	65	67.4	Yes	Yes
3000	67	71.86	Yes	Yes
4000	67	72	No	Yes
6000	70	64	No	Yes
8000	50	60.2	No	No

TABLE V. Shows the individual Targets (dB SPL) for Male Left Ear 60-69 G.R.A.S KEMAR Right Ear ISTS 65 dB SPL for each Frequencies (Hz), and whether the device meets them under two criteria

Frequencies (Hz)	Response (dB SPL)	Targets (dB SPL)	Meets Strict Criteria	Meets Loose Criteria
250	41	41.36	Yes	Yes
500	46	43.3	Yes	Yes
750	48	42.7	No	Yes
1000	51	42.9	No	Yes
1500	53	48.9	Yes	Yes
2000	55	56	Yes	Yes
3000	59	61.95	Yes	Yes
4000	61	64	Yes	Yes
6000	69	53.6	No	No
8000	57	48.77	No	Yes

TABLE VI. Shows the individual Targets (dB SPL) for Male Right Ear 60-69 ISTS 55 dB SPL for each Frequencies (Hz), and whether the device meets them under two criteria

.

Frequencies (Hz)	Response (dB SPL)	Targets (dB SPL)	Meets Strict Criteria	Meets Loose Criteria
250	48	55.67	No	Yes
500	61	57	Yes	Yes
750	62	56	No	Yes
1000	65	56	No	Yes
1500	66	60.4	No	Yes
2000	67	65	Yes	Yes
3000	71	70	Yes	Yes
4000	74	71.19	Yes	Yes
6000	81	62.6	No	No
8000	67	59.05	No	Yes

TABLE VII. Shows the individual Targets (dB SPL) for Male Right Ear 60-69 ISTS 65 dB SPL for each Frequencies (Hz), and whether the device meets them under two criteria

.

Frequencies (Hz)	Response (dB SPL)	Targets (dB SPL)	Meets Strict Criteria	Meets Loose Criteria
250	54	71	No	No
500	69	72	Yes	Yes
750	71	68.88	Yes	Yes
1000	76	66.89	No	Yes
1500	79	69.9	No	Yes
2000	81	73.55	No	Yes
3000	85	76.03	No	Yes
4000	89	76.8	No	No
6000	92	69.4	No	No
8000	76	66.51	No	Yes

TABLE VIII. Shows the individual Targets (dB SPL) for Male Right Ear 60-69 ISTS 80 dB SPL for each Frequencies (Hz), and whether the device meets them under two criteria

.

Frequencies (Hz)	Response (dB SPL)	Targets (dB SPL)	Meets Strict Criteria	Meets Loose Criteria
250	25	55.67	No	No
500	48	57	No	Yes
750	54	56	Yes	Yes
1000	55	56	Yes	Yes
1500	60	60.4	Yes	Yes
2000	65	65	Yes	Yes
3000	67	70	Yes	Yes
4000	67	71.19	Yes	Yes
6000	70	62.6	No	Yes
8000	50	59.05	No	Yes

TABLE IX. Shows the individual Targets (dB SPL) for Male Right Ear 60-69 G.R.A.S KEMAR Left Ear ISTS 65 dB SPL for each Frequencies (Hz), and whether the device meets them under two criteria

Frequencies (Hz)	Response (dB SPL)	Targets (dB SPL)	Meets Strict Criteria	Meets Loose Criteria
250	39	55.67	No	No
500	53	57	Yes	Yes
750	58	56	Yes	Yes
1000	56	56	Yes	Yes
1500	55	60	Yes	Yes
2000	62	65	Yes	Yes
3000	67	70	Yes	Yes
4000	66	71	Yes	Yes
6000	69	62.6	No	Yes
8000	55	59.05	Yes	Yes

TABLE X. Shows the individual Targets (dB SPL) for Male Right Ear 60-69 G.R.A.S KEMAR Right Ear ISTS 65 dB SPL for each Frequencies (Hz), and whether the device meets them under two criteria

Frequencies (Hz)	Response (dB SPL)	Targets (dB SPL)	Meets Strict Criteria	Meets Loose Criteria
250	41	41.35	Yes	Yes
500	46	47.72	Yes	Yes
750	48	48.55	Yes	Yes
1000	51	49.66	Yes	Yes
1500	53	56.58	Yes	Yes
2000	55	64.334	No	Yes
3000	59	67.94	No	Yes
4000	61	67.94	No	Yes
6000	69	59.07	No	Yes
8000	57	54.64	Yes	Yes

TABLE XI. Shows the individual Targets (dB SPL) for Male Left Ear 70-79 ISTS 55 dB SPL for each Frequencies (Hz), and whether the device meets them under two criteria

.

Frequencies (Hz)	Response (dB SPL)	Targets (dB SPL)	Meets Strict Criteria	Meets Loose Criteria
250	48	55.28	No	Yes
500	61	57.2	Yes	Yes
750	62	58.29	Yes	Yes
1000	65	59.68	No	Yes
1500	66	65.27	Yes	Yes
2000	67	71	Yes	Yes
3000	71	75.19	Yes	Yes
4000	74	75.54	Yes	Yes
6000	81	67.5	No	No
8000	67	63.72	Yes	Yes

TABLE XII. Shows the individual Targets (dB SPL) for Male Left Ear 70-79 ISTS 65 dB SPL for each Frequencies (Hz), and whether the device meets them under two criteria

.

Frequencies (Hz)	Response (dB SPL)	Targets (dB SPL)	Meets Strict Criteria	Meets Loose Criteria
250	54	71	No	No
500	69	72.7	Yes	Yes
750	71	69.78	Yes	Yes
1000	76	69.17	No	Yes
1500	79	73.2	No	Yes
2000	81	77.85	Yes	Yes
3000	85	81.02	Yes	Yes
4000	89	81.58	No	Yes
6000	92	74.56	No	No
8000	76	71.62	Yes	Yes

TABLE XIII. Shows the individual Targets (dB SPL) for Male Left Ear 70-79 ISTS 80 dB SPL for each Frequencies (Hz), and whether the device meets them under two criteria

.

Frequencies (Hz)	Response (dB SPL)	Targets (dB SPL)	Meets Strict Criteria	Meets Loose Criteria
250	39	55.28	No	No
500	53	57.2	Yes	Yes
750	58	58.29	Yes	Yes
1000	56	59.68	Yes	Yes
1500	55	65.27	No	No
2000	62	71	No	Yes
3000	67	75.19	No	Yes
4000	66	75.54	No	Yes
6000	69	67.5	Yes	Yes
8000	55	63.72	No	Yes

TABLE XIV. Shows the individual Targets (dB SPL) for Male Left Ear 70-79 G.R.A.S KEMAR Right Ear ISTS 65 dB SPL for each Frequencies (Hz), and whether the device meets them under two criteria

Frequencies (Hz)	Response (dB SPL)	Targets (dB SPL)	Meets Strict Criteria	Meets Loose Criteria
250	25	55.28	No	No
500	48	57.2	No	Yes
750	54	58.29	Yes	Yes
1000	55	59.68	Yes	Yes
1500	60	65.27	No	Yes
2000	65	71	No	Yes
3000	67	75.19	No	Yes
4000	67	75.54	No	Yes
6000	70	67.5	Yes	Yes
8000	50	63.72	No	No

TABLE XV. Shows the individual Targets (dB SPL) for Male Left Ear 70-79 G.R.A.S KEMAR Left Ear ISTS 65 dB SPL for each Frequencies (Hz), and whether the device meets them under two criteria

Frequencies (Hz)	Response (dB SPL)	Targets (dB SPL)	Meets Strict Criteria	Meets Loose Criteria
250	41	41.14	Yes	Yes
500	46	48.28	Yes	Yes
750	48	47.43	Yes	Yes
1000	51	48	Yes	Yes
1500	53	53.43	Yes	Yes
2000	55	60.28	No	Yes
3000	59	64.28	No	Yes
4000	61	64.28	Yes	Yes
6000	69	55.71	No	No
8000	57	52.28	Yes	Yes

TABLE XVI. Shows the individual Targets (dB SPL) for Male Right Ear 70-79 ISTS 55 dB SPL for each Frequencies (Hz), and whether the device meets them under two criteria

.

Frequencies (Hz)	Response (dB SPL)	Targets (dB SPL)	Meets Strict Criteria	Meets Loose Criteria
250	48	56	No	Yes
500	61	57.45	Yes	Yes
750	62	58.3	Yes	Yes
1000	65	58.86	No	Yes
1500	66	63.39	Yes	Yes
2000	67	68.207	Yes	Yes
3000	71	72.17	Yes	Yes
4000	74	72.17	Yes	Yes
6000	81	64.52	No	No
8000	67	61.13	No	Yes

TABLE XVII. Shows the individual Targets (dB SPL) for Male Right Ear 70-79 ISTS 65 dB SPL for each Frequencies (Hz), and whether the device meets them under two criteria

.

Frequencies (Hz)	Response (dB SPL)	Targets (dB SPL)	Meets Strict Criteria	Meets Loose Criteria
250	54	70.8	No	No
500	69	72.3	Yes	Yes
750	71	69.19	Yes	Yes
1000	76	68.6	No	Yes
1500	79	71.8	No	Yes
2000	81	75.8	No	Yes
3000	85	78.44	No	Yes
4000	89	78.46	No	No
6000	92	71.6	No	No
8000	76	69.1	No	Yes

TABLE XVIII. Shows the individual Targets (dB SPL) for Male Right Ear 70-79 ISTS 80 dB SPL for each Frequencies (Hz), and whether the device meets them under two criteria

.

Frequencies (Hz)	Response (dB SPL)	Targets (dB SPL)	Meets Strict Criteria	Meets Loose Criteria
250	39	56	No	No
500	53	57.45	Yes	Yes
750	58	58.3	Yes	Yes
1000	56	58.86	Yes	Yes
1500	55	63.39	No	No
2000	62	68.207	No	Yes
3000	67	72.17	No	Yes
4000	66	72.17	No	Yes
6000	69	64.52	Yes	Yes
8000	55	61.13	No	Yes

TABLE XIX. Shows the individual Targets (dB SPL) for Male Right Ear 70-79 G.R.A.S KEMAR Right Ear ISTS 65 dB SPL for each Frequencies (Hz), and whether the device meets them under two criteria

Frequencies (Hz)	Response (dB SPL)	Targets (dB SPL)	Meets Strict Criteria	Meets Loose Criteria
250	25	56	No	No
500	48	57.45	No	Yes
750	54	58.3	Yes	Yes
1000	55	58.86	Yes	Yes
1500	60	63.39	Yes	Yes
2000	65	68.207	Yes	Yes
3000	67	72.17	No	Yes
4000	67	72.17	No	Yes
6000	70	64.52	No	Yes
8000	50	61.13	No	No

TABLE XX. Shows the individual Targets (dB SPL) for Male Right Ear 70-79 G.R.A.S KEMAR Left Ear ISTS 65 dB SPL for each Frequencies (Hz), and whether the device meets them under two criteria

Frequencies (Hz)	Response (dB SPL)	Targets (dB SPL)	Meets Strict Criteria	Meets Loose Criteria
250	41	41.35	Yes	Yes
500	46	47.72	Yes	Yes
750	48	48.55	Yes	Yes
1000	51	49.66	Yes	Yes
1500	53	56.58	Yes	Yes
2000	55	64.334	No	Yes
3000	59	67.94	No	Yes
4000	61	67.94	No	Yes
6000	69	59.07	No	Yes
8000	57	54.64	Yes	Yes

TABLE XXI. Shows the individual Targets (dB SPL) for Male Left Ear 70-79 ISTS 55 dB SPL for each Frequencies (Hz), and whether the device meets them under two criteria

.

Frequencies (Hz)	Response (dB SPL)	Targets (dB SPL)	Meets Strict Criteria	Meets Loose Criteria
250	48	55.28	No	Yes
500	61	57.2	Yes	Yes
750	62	58.29	Yes	Yes
1000	65	59.68	No	Yes
1500	66	65.27	Yes	Yes
2000	67	71	Yes	Yes
3000	71	75.19	Yes	Yes
4000	74	75.54	Yes	Yes
6000	81	67.5	No	No
8000	67	63.72	Yes	Yes

TABLE XXII. Shows the individual Targets (dB SPL) for Male Left Ear 70-79 ISTS 65 dB SPL for each Frequencies (Hz), and whether the device meets them under two criteria

.

Frequencies (Hz)	Response (dB SPL)	Targets (dB SPL)	Meets Strict Criteria	Meets Loose Criteria
250	54	71	No	No
500	69	72.7	Yes	Yes
750	71	69.78	Yes	Yes
1000	76	69.17	No	Yes
1500	79	73.2	No	Yes
2000	81	77.85	Yes	Yes
3000	85	81.02	Yes	Yes
4000	89	81.58	No	Yes
6000	92	74.56	No	No
8000	76	71.62	Yes	Yes

TABLE XXIII. Shows the individual Targets (dB SPL) for Male Left Ear 70-79 ISTS 80 dB SPL for each Frequencies (Hz), and whether the device meets them under two criteria

.

Frequencies (Hz)	Response (dB SPL)	Targets (dB SPL)	Meets Strict Criteria	Meets Loose Criteria
250	39	56	No	No
500	53	57.45	Yes	Yes
750	58	58.3	Yes	Yes
1000	56	58.86	Yes	Yes
1500	55	63.39	No	No
2000	62	68.207	No	Yes
3000	67	72.17	No	Yes
4000	66	72.17	No	Yes
6000	69	64.52	Yes	Yes
8000	55	61.13	No	Yes

TABLE XXIV. Shows the individual Targets (dB SPL) for Male Left Ear 70-79 G.R.A.S KEMAR Right Ear ISTS 65 dB SPL for each Frequencies (Hz), and whether the device meets them under two criteria

Frequencies (Hz)	Response (dB SPL)	Targets (dB SPL)	Meets Strict Criteria	Meets Loose Criteria
250	25	55.28	No	No
500	48	57.2	No	Yes
750	54	58.29	Yes	Yes
1000	55	59.68	Yes	Yes
1500	60	65.27	No	Yes
2000	65	71	No	Yes
3000	67	75.19	No	Yes
4000	67	75.54	No	Yes
6000	70	67.5	Yes	Yes
8000	50	63.72	No	No

TABLE XXV. Shows the individual Targets (dB SPL) for Male Left Ear 70-79 G.R.A.S KEMAR Left Ear ISTS 65 dB SPL for each Frequencies (Hz), and whether the device meets them under two criteria

Frequencies (Hz)	Response (dB SPL)	Targets (dB SPL)	Meets Strict Criteria	Meets Loose Criteria
250	41	41.08	Yes	Yes
500	46	44.44	Yes	Yes
750	48	41.56	No	Yes
1000	51	40.11	No	No
1500	53	45.49	No	Yes
2000	55	51.16	Yes	Yes
3000	59	54.83	Yes	Yes
4000	61	53.95	No	Yes
6000	69	45.09	No	No
8000	57	41.37	No	No

TABLE XXVI. Shows the individual Targets (dB SPL) for Female Left Ear 60-69 ISTS 55 dB SPL for each Frequencies (Hz), and whether the device meets them under two criteria

.

Frequencies (Hz)	Response (dB SPL)	Targets (dB SPL)	Meets Strict Criteria	Meets Loose Criteria
250	48	56.03	No	Yes
500	61	57.59	Yes	Yes
750	62	54.45	No	Yes
1000	65	53.8	No	No
1500	66	57.59	No	Yes
2000	67	61.98	No	Yes
3000	71	64.808	No	Yes
4000	74	64.5	No	Yes
6000	81	56.18	No	No
8000	67	52.42	No	No

TABLE XXVII. Shows the individual Targets (dB SPL) for Female Left Ear 60-69 ISTS 65 dB SPL for each Frequencies (Hz), and whether the device meets them under two criteria

.

Frequencies (Hz)	Response (dB SPL)	Targets (dB SPL)	Meets Strict Criteria	Meets Loose Criteria
250	54	70.89	No	No
500	69	72.77	Yes	Yes
750	71	68.91	Yes	Yes
1000	76	66.23	No	Yes
1500	79	68.61	No	No
2000	81	71.28	No	Yes
3000	85	73.36	No	No
4000	89	72.62	No	No
6000	92	65.05	No	No
8000	76	61.63	No	No

TABLE XXVIII. Shows the individual Targets (dB SPL) for Female Left Ear 60-69 ISTS 80 dB SPL for each Frequencies (Hz), and whether the device meets them under two criteria

.

Frequencies (Hz)	Response (dB SPL)	Targets (dB SPL)	Meets Strict Criteria	Meets Loose Criteria
250	39	56.03	No	No
500	53	57.59	Yes	Yes
750	58	54.45	Yes	Yes
1000	56	53.8	Yes	Yes
1500	55	57.59	Yes	Yes
2000	62	61.98	Yes	Yes
3000	67	64.808	Yes	Yes
4000	66	64.5	Yes	Yes
6000	69	56.18	No	No
8000	55	52.42	Yes	Yes

TABLE XXIX. Shows the individual Targets (dB SPL) for Female Left Ear 60-69 G.R.A.S KEMAR Right Ear ISTS 65 dB SPL for each Frequencies (Hz), and whether the device meets them under two criteria.

Frequencies (Hz)	Response (dB SPL)	Targets (dB SPL)	Meets Strict Criteria	Meets Loose Criteria
250	25	56	No	No
500	48	57.59	No	Yes
750	54	54.45	Yes	Yes
1000	55	53.8	Yes	Yes
1500	60	57.59	Yes	Yes
2000	65	61.98	Yes	Yes
3000	67	64.808	Yes	Yes
4000	67	64.5	Yes	Yes
6000	70	56.18	No	No
8000	50	52.42	Yes	Yes

TABLE XXX. Shows the individual Targets (dB SPL) for Female Left Ear 60-69 G.R.A.S KEMAR Left Ear ISTS 65 dB SPL for each Frequencies (Hz), and whether the device meets them under two criteria.

Frequencies (Hz)	Response (dB SPL)	Targets (dB SPL)	Meets Strict Criteria	Meets Loose Criteria
250	41	41.18	Yes	Yes
500	46	43.36	Yes	Yes
750	48	41.13	No	Yes
1000	51	39.89	No	No
1500	53	45.71	No	Yes
2000	55	51.8	Yes	Yes
3000	59	54.8	Yes	Yes
4000	61	53.7	No	Yes
6000	69	44.55	No	No
8000	57	40.8	No	No

TABLE XXXI. Shows the individual Targets (dB SPL) for Female Right Ear 60-69 ISTS 55 dB SPL for each Frequencies (Hz), and whether the device meets them under two criteria

.

Frequencies (Hz)	Response (dB SPL)	Targets (dB SPL)	Meets Strict Criteria	Meets Loose Criteria
250	48	55.6	No	Yes
500	61	57.54	Yes	Yes
750	62	54.66	No	Yes
1000	65	54.07	No	No
1500	66	57.45	No	Yes
2000	67	61.41	No	Yes
3000	71	64.95	No	Yes
4000	74	63.06	No	No
6000	81	55.06	No	No
8000	67	50.81	No	No

TABLE XXXII. Shows the individual Targets (dB SPL) for Female Right Ear 60-69 ISTS 65 dB SPL for each Frequencies (Hz), and whether the device meets them under two criteria

.

Frequencies (Hz)	Response (dB SPL)	Targets (dB SPL)	Meets Strict Criteria	Meets Loose Criteria
250	54	70.89	No	No
500	69	72.77	Yes	Yes
750	71	68.91	Yes	Yes
1000	76	66.23	No	Yes
1500	79	68.61	No	No
2000	81	71.28	No	Yes
3000	85	73.36	No	No
4000	89	72.62	No	No
6000	92	65.05	No	No
8000	76	61.63	No	No

TABLE XXXIII. Shows the individual Targets (dB SPL) for Female Right Ear 60-69 ISTS 80 dB SPL for each Frequencies (Hz), and whether the device meets them under two criteria

.

Frequencies (Hz)	Response (dB SPL)	Targets (dB SPL)	Meets Strict Criteria	Meets Loose Criteria
250	39	55.6	No	No
500	53	57.54	Yes	Yes
750	58	54.66	Yes	Yes
1000	56	54.07	Yes	Yes
1500	55	57.45	Yes	Yes
2000	62	61.41	Yes	Yes
3000	67	64.95	Yes	Yes
4000	66	63.06	Yes	Yes
6000	69	55.06	No	No
8000	55	50.81	Yes	Yes

TABLE XXXIV. Shows the individual Targets (dB SPL) for Female Right Ear 60-69 G.R.A.S KEMAR Right Ear ISTS 65 dB SPL for each Frequencies (Hz), and whether the device meets them under two criteria.

Frequencies (Hz)	Response (dB SPL)	Targets (dB SPL)	Meets Strict Criteria	Meets Loose Criteria
250	25	55.6	No	No
500	48	57.54	No	Yes
750	54	54.66	Yes	Yes
1000	55	54.07	Yes	Yes
1500	60	57.45	Yes	Yes
2000	65	61.41	Yes	Yes
3000	67	64.95	Yes	Yes
4000	67	63.06	Yes	Yes
6000	70	55.06	No	No
8000	50	50.81	Yes	Yes

TABLE XXXV. Shows the individual Targets (dB SPL) for Female Right Ear 60-69 G.R.A.S KEMAR Left Ear ISTS 65 dB SPL for each Frequencies (Hz), and whether the device meets them under two criteria.

Frequencies (Hz)	Response (dB SPL)	Targets (dB SPL)	Meets Strict Criteria	Meets Loose Criteria
250	41	41.09	Yes	Yes
500	46	46.34	Yes	Yes
750	48	44.83	Yes	Yes
1000	51	44.45	No	Yes
1500	53	50.37	Yes	Yes
2000	55	56.83	Yes	Yes
3000	59	59.94	Yes	Yes
4000	61	58.98	Yes	Yes
6000	69	50.84	No	No
8000	57	47.41	No	Yes

TABLE XXXVI. Shows the individual Targets (dB SPL) for Female Left Ear 70-79 ISTS 55 dB SPL for each Frequencies (Hz), and whether the device meets them under two criteria

.

Frequencies (Hz)	Response (dB SPL)	Targets (dB SPL)	Meets Strict Criteria	Meets Loose Criteria
250	48	55.79	No	Yes
500	61	57.75	Yes	Yes
750	62	57.47	Yes	Yes
1000	65	57.75	No	Yes
1500	66	61.68	Yes	Yes
2000	67	66.44	Yes	Yes
3000	71	68.41	Yes	Yes
4000	74	68.41	No	Yes
6000	81	60.28	No	No
8000	67	57.19	No	Yes

TABLE XXXVII. Shows the individual Targets (dB SPL) for Female Left Ear 70-79 ISTS 65 dB SPL for each Frequencies (Hz), and whether the device meets them under two criteria

.

Frequencies (Hz)	Response (dB SPL)	Targets (dB SPL)	Meets Strict Criteria	Meets Loose Criteria
250	54	71.21	No	No
500	69	72.64	Yes	Yes
750	71	69.29	Yes	Yes
1000	76	68.17	No	Yes
1500	79	70.44	No	Yes
2000	81	74.11	No	Yes
3000	85	75.53	No	Yes
4000	89	75.26	No	No
6000	92	67.4	No	No
8000	76	64.88	No	No

TABLE XXXVIII. Shows the individual Targets (dB SPL) for Female Left Ear 70-79 ISTS 80 dB SPL for each Frequencies (Hz), and whether the device meets them under two criteria

.

Frequencies (Hz)	Response (dB SPL)	Targets (dB SPL)	Meets Strict Criteria	Meets Loose Criteria
250	39	55.95	No	No
500	53	57.47	Yes	Yes
750	58	56.17	Yes	Yes
1000	56	56.78	Yes	Yes
1500	55	60.23	No	Yes
2000	62	65.91	Yes	Yes
3000	67	67.77	Yes	Yes
4000	66	66.8	Yes	Yes
6000	69	59.47	No	Yes
8000	55	55.96	Yes	Yes

TABLE XXXIX. Shows the individual Targets (dB SPL) for Female Left Ear 70-79 G.R.A.S KEMAR Right Ear ISTS 65 dB SPL for each Frequencies (Hz), and whether the device meets them under two criteria.

Frequencies (Hz)	Response (dB SPL)	Targets (dB SPL)	Meets Strict Criteria	Meets Loose Criteria
250	25	55.95	No	No
500	48	57.47	No	Yes
750	54	56.17	Yes	Yes
1000	55	56.78	Yes	Yes
1500	60	60.23	Yes	Yes
2000	65	65.91	Yes	Yes
3000	67	67.77	Yes	Yes
4000	67	66.8	Yes	Yes
6000	70	59.47	No	No
8000	50	55.96	No	Yes

TABLE XL. Shows the individual Targets (dB SPL) for Female Left Ear 70-79 G.R.A.S KEMAR Left Ear ISTS 65 dB SPL for each Frequencies (Hz), and whether the device meets them under two criteria.

Frequencies (Hz)	Response (dB SPL)	Targets (dB SPL)	Meets Strict Criteria	Meets Loose Criteria
250	41	40.87	Yes	Yes
500	46	47.69	Yes	Yes
750	48	46.25	Yes	Yes
1000	51	46.22	Yes	Yes
1500	53	51.53	Yes	Yes
2000	55	57.17	Yes	Yes
3000	59	60.24	Yes	Yes
4000	61	59.93	Yes	Yes
6000	69	51.01	No	No
8000	57	47.467	No	Yes

TABLE XLI. Shows the individual Targets (dB SPL) for Female Right Ear 70-79 ISTS 55 dB SPL for each Frequencies (Hz), and whether the device meets them under two criteria

.

Frequencies (Hz)	Response (dB SPL)	Targets (dB SPL)	Meets Strict Criteria	Meets Loose Criteria
250	48	55.79	No	Yes
500	61	57.75	Yes	Yes
750	62	57.47	Yes	Yes
1000	65	57.75	No	Yes
1500	66	61.68	Yes	Yes
2000	67	66.44	Yes	Yes
3000	71	68.41	Yes	Yes
4000	74	68.41	No	Yes
6000	81	60.28	No	No
8000	67	57.19	No	Yes

TABLE XLII. Shows the individual Targets (dB SPL) for Female Right Ear 70-79 ISTS 65 dB SPL for each Frequencies (Hz), and whether the device meets them under two criteria.

Frequencies (Hz)	Response (dB SPL)	Targets (dB SPL)	Meets Strict Criteria	Meets Loose Criteria
250	54	71.03	No	No
500	69	72.17	Yes	Yes
750	71	69.34	Yes	Yes
1000	76	67.64	No	Yes
1500	79	70.47	No	Yes
2000	81	74.43	No	Yes
3000	85	75.56	No	Yes
4000	89	75.28	No	No
6000	92	67.92	No	No
8000	76	65.09	No	No

TABLE XLIII. Shows the individual Targets (dB SPL) for Female Right Ear 70-79 ISTS 80 dB SPL for each Frequencies (Hz), and whether the device meets them under two criteria.

Frequencies (Hz)	Response (dB SPL)	Targets (dB SPL)	Meets Strict Criteria	Meets Loose Criteria
250	39	55.79	No	No
500	53	57.75	Yes	Yes
750	58	57.47	Yes	Yes
1000	56	57.75	Yes	Yes
1500	55	61.68	No	Yes
2000	62	66.44	Yes	Yes
3000	67	68.41	Yes	Yes
4000	66	68.41	Yes	Yes
6000	69	60.28	No	Yes
8000	55	57.19	Yes	Yes

TABLE XLIV. Shows the individual Targets (dB SPL) for Female Right Ear 70-79 G.R.A.S KEMAR Right Ear ISTS 65 dB SPL for each Frequencies (Hz), and whether the device meets them under two criteria.

Frequencies (Hz)	Response (dB SPL)	Targets (dB SPL)	Meets Strict Criteria	Meets Loose Criteria
250	25	55.79	No	No
500	48	57.75	No	Yes
750	54	57.47	Yes	Yes
1000	55	57.75	Yes	Yes
1500	60	61.68	Yes	Yes
2000	65	66.44	Yes	Yes
3000	67	68.41	Yes	Yes
4000	67	68.41	Yes	Yes
6000	70	60.28	No	Yes
8000	50	57.19	No	Yes

TABLE XLV. Shows the individual Targets (dB SPL) for Female Right Ear 70-79 G.R.A.S KEMAR Left Ear ISTS 65 dB SPL for each Frequencies (Hz), and whether the device meets them under two criteria.

Frequencies (Hz)	Response (dB SPL)	Targets (dB SPL)	Meets Strict Criteria	Meets Loose Criteria
250	41	40.89	Yes	Yes
500	46	45.4	Yes	Yes
750	48	41.81	No	Yes
1000	51	40.31	No	No
1500	53	45.21	No	Yes
2000	55	51.23	Yes	Yes
3000	59	53.91	No	Yes
4000	61	52.54	No	Yes
6000	69	44.2	No	No
8000	57	40	No	No

TABLE XLVI. Shows the individual Targets (dB SPL) for Hearing Profile X ISTS 55 dB SPL for each Frequencies (Hz), and whether the device meets them under two criteria.

Frequencies (Hz)	Response (dB SPL)	Targets (dB SPL)	Meets Strict Criteria	Meets Loose Criteria
250	48	56	No	Yes
500	61	57.7	Yes	Yes
750	62	54.77	No	Yes
1000	65	54.35	No	No
1500	66	57.85	No	Yes
2000	67	61.77	No	Yes
3000	71	63.31	No	Yes
4000	74	62.47	No	No
6000	81	54.63	No	No
8000	67	50.43	No	No

TABLE XLVII. Shows the individual Targets (dB SPL) for Hearing Profile X ISTS 65 dB SPL for each Frequencies (Hz), and whether the device meets them under two criteria.

Frequencies (Hz)	Response (dB SPL)	Targets (dB SPL)	Meets Strict Criteria	Meets Loose Criteria
250	54	70.82	No	No
500	69	72.65	Yes	Yes
750	71	69.46	Yes	Yes
1000	76	67.64	No	Yes
1500	79	68.7	No	No
2000	81	71.59	No	Yes
3000	85	72.65	No	No
4000	89	71.12	No	No
6000	92	63.23	No	No
8000	76	59.59	No	No

TABLE XLVIII. Shows the individual Targets (dB SPL) for Hearing Profile X ISTS 80 dB SPL for each Frequencies (Hz), and whether the device meets them under two criteria.

Frequencies (Hz)	Response (dB SPL)	Targets (dB SPL)	Meets Strict Criteria	Meets Loose Criteria
250	39	56	No	No
500	53	57.7	No	Yes
750	58	54.77	Yes	Yes
1000	56	54.35	Yes	Yes
1500	55	57.85	No	Yes
2000	62	61.77	Yes	Yes
3000	67	63.31	Yes	Yes
4000	66	62.47	Yes	Yes
6000	69	54.63	No	Yes
8000	55	50.43	Yes	Yes

TABLE XLIX. Shows the individual Targets (dB SPL) for Hearing Profile X G.R.A.S KEMAR Right Ear ISTS 65 dB SPL for each Frequencies (Hz), and whether the device meets them under two criteria.

Frequencies (Hz)	Response (dB SPL)	Targets (dB SPL)	Meets Strict Criteria	Meets Loose Criteria
250	25	56	No	No
500	48	57.7	No	Yes
750	54	54.77	Yes	Yes
1000	55	54.35	Yes	Yes
1500	60	57.85	Yes	Yes
2000	65	61.77	Yes	Yes
3000	67	63.31	Yes	Yes
4000	67	62.47	Yes	Yes
6000	70	54.63	No	No
8000	50	50.43	Yes	Yes

TABLE L. Shows the individual Targets (dB SPL) for Hearing Profile X G.R.A.S KEMAR Left Ear ISTS 65 dB SPL for each Frequencies (Hz), and whether the device meets them under two criteria.

Frequencies (Hz)	Response (dB SPL)	Targets (dB SPL)	Meets Strict Criteria	Meets Loose Criteria
250	41	41.04	Yes	Yes
500	46	49.51	Yes	Yes
750	48	48.15	Yes	Yes
1000	51	47.85	Yes	Yes
1500	53	52.39	Yes	Yes
2000	55	58.79	Yes	Yes
3000	59	61.82	Yes	Yes
4000	61	61.61	Yes	Yes
6000	69	53.75	No	No
8000	57	50.11	No	Yes

TABLE LI. Shows the individual Targets (dB SPL) for Hearing Profile Y ISTS 55 dB SPL for each Frequencies (Hz), and whether the device meets them under two criteria.

Frequencies (Hz)	Response (dB SPL)	Targets (dB SPL)	Meets Strict Criteria	Meets Loose Criteria
250	48	56.14	No	Yes
500	61	59.14	Yes	Yes
750	62	58.84	Yes	Yes
1000	65	59.14	No	No
1500	66	62.75	Yes	Yes
2000	67	66.96	Yes	Yes
3000	71	69.96	Yes	Yes
4000	74	70.11	Yes	Yes
6000	81	62.75	No	No
8000	67	58.99	No	Yes

TABLE LII. Shows the individual Targets (dB SPL) for Hearing Profile Y ISTS 65 dB SPL for each Frequencies (Hz), and whether the device meets them under two criteria.

Frequencies (Hz)	Response (dB SPL)	Targets (dB SPL)	Meets Strict Criteria	Meets Loose Criteria
250	54	71.14	No	No
500	69	72.57	Yes	Yes
750	71	69.42	Yes	Yes
1000	76	68.85	No	Yes
1500	79	71.14	No	Yes
2000	81	74.85	No	Yes
3000	85	76.285	No	Yes
4000	89	77.14	No	No
6000	92	69.14	No	No
8000	76	66.85	No	Yes

TABLE LIII. Shows the individual Targets (dB SPL) for Hearing Profile Y ISTS 80 dB SPL for each Frequencies (Hz), and whether the device meets them under two criteria.

Frequencies (Hz)	Response (dB SPL)	Targets (dB SPL)	Meets Strict Criteria	Meets Loose Criteria
250	39	56	No	No
500	53	57.7	Yes	Yes
750	58	54.77	Yes	Yes
1000	56	54.35	Yes	Yes
1500	55	57.85	Yes	Yes
2000	62	61.77	Yes	Yes
3000	67	69.96	Yes	Yes
4000	66	70.11	Yes	Yes
6000	69	62.75	No	No
8000	55	58.99	Yes	Yes

TABLE LIV. Shows the individual Targets (dB SPL) for Hearing Profile Y G.R.A.S KEMAR Right Ear ISTS 65 dB SPL for each Frequencies (Hz), and whether the device meets them under two criteria.

Frequencies (Hz)	Response (dB SPL)	Targets (dB SPL)	Meets Strict Criteria	Meets Loose Criteria
250	25	56.14	No	No
500	48	59.14	No	No
750	54	58.84	Yes	Yes
1000	55	59.14	Yes	Yes
1500	60	62.75	Yes	Yes
2000	65	66.96	Yes	Yes
3000	67	69.96	Yes	Yes
4000	67	70.11	Yes	Yes
6000	70	62.75	No	Yes
8000	50	58.99	No	Yes

TABLE LV. Shows the individual Targets (dB SPL) for Hearing Profile Y G.R.A.S KEMAR Left Ear ISTS 65 dB SPL for each Frequencies (Hz), and whether the device meets them under two criteria.

Frequencies (Hz)	Response (dB SPL)	Targets (dB SPL)	Meets Strict Criteria	Meets Loose Criteria
250	41	41.46	Yes	Yes
500	46	48.44	Yes	Yes
750	48	49.08	Yes	Yes
1000	51	50.61	Yes	Yes
1500	53	58.78	No	Yes
2000	55	67.13	No	No
3000	59	71.18	No	No
4000	61	70.8	No	Yes
6000	69	61.91	No	Yes
8000	57	57.11	Yes	Yes

TABLE LVI. Shows the individual Targets (dB SPL) for Hearing Profile Z ISTS 55 dB SPL for each Frequencies (Hz), and whether the device meets them under two criteria.

Frequencies (Hz)	Response (dB SPL)	Targets (dB SPL)	Meets Strict Criteria	Meets Loose Criteria
250	48	56.76	No	Yes
500	61	57.91	Yes	Yes
750	62	59.08	Yes	Yes
1000	65	60.86	Yes	No
1500	66	67.74	Yes	Yes
2000	67	74.64	No	Yes
3000	71	78.82	No	Yes
4000	74	69.4	Yes	Yes
6000	81	70.83	No	No
8000	67	67.598	Yes	Yes

TABLE LVII. Shows the individual Targets (dB SPL) for Hearing Profile Z ISTS 65 dB SPL for each Frequencies (Hz), and whether the device meets them under two criteria.

Frequencies (Hz)	Response (dB SPL)	Targets (dB SPL)	Meets Strict Criteria	Meets Loose Criteria
250	54	70.86	No	No
500	69	72.33	Yes	Yes
750	71	69.44	Yes	Yes
1000	76	69.7	No	Yes
1500	79	74.25	Yes	Yes
2000	81	80.53	Yes	Yes
3000	85	84.51	Yes	Yes
4000	89	84.92	Yes	Yes
6000	92	78.15	No	No
8000	76	74.68	Yes	Yes

TABLE LVIII. Shows the individual Targets (dB SPL) for Hearing Profile Z ISTS 80 dB SPL for each Frequencies (Hz), and whether the device meets them under two criteria.

Frequencies (Hz)	Response (dB SPL)	Targets (dB SPL)	Meets Strict Criteria	Meets Loose Criteria
250	39	56.76	No	No
500	53	57.91	Yes	Yes
750	58	59	Yes	Yes
1000	56	60	Yes	Yes
1500	55	67.74	No	No
2000	62	74.64	No	No
3000	67	78.82	No	No
4000	66	69.4	Yes	Yes
6000	69	70.83	Yes	Yes
8000	55	67.598	No	No

TABLE LIX. Shows the individual Targets (dB SPL) for Hearing Profile Z G.R.A.S KEMAR Right Ear ISTS 65 dB SPL for each Frequencies (Hz), and whether the device meets them under two criteria.

Frequencies (Hz)	Response (dB SPL)	Targets (dB SPL)	Meets Strict Criteria	Meets Loose Criteria
250	25	56.76	No	No
500	48	57.91	No	Yes
750	54	59	Yes	Yes
1000	55	60	Yes	Yes
1500	60	67.74	No	Yes
2000	65	74.64	No	Yes
3000	67	78.82	No	No
4000	67	69.4	Yes	Yes
6000	70	70.83	Yes	Yes
8000	50	67.598	No	No

TABLE LX. Shows the individual Targets (dB SPL) for Hearing Profile Z G.R.A.S KEMAR Left Ear ISTS 65 dB SPL for each Frequencies (Hz), and whether the device meets them under two criteria.
