# Supplementary material for: LoCHAid: An ultra-low-cost hearing aid for age-related hearing loss
Source: PLoS One. 2020 Sep 23;15(9):e0238922. doi: 10.1371/journal.pone.0238922 (PMC7510997; doi:10.1371/journal.pone.0238922)
Supplement: Attachment — Submitted filename: PLOS One Reviewer Edits.pdf [file pone.0238922.s006.pdf]

Thank you to Reviewer 1 and 2 for the comments - below we have provided a line by line response to the reviewers' comments.

Reviewer 1 - Comments

There are a number of points where the authors should consider revisions. These are:

The Abstract claims the h/aid "costs only 98 cents (< $1) to mass manufacturer". However, the text states that mass manufacture would indeed cost substantially more than this. The authors should take care not to "over-sell" their proof-of-concept device in the Abstract.

- We have edited this to include that the hearing aid total component costs only 98 cents to mass manufacture. (Line 17-18). The sentence now reads:

  The LoCHAid components cost 98 cents (<$1) when purchased in bulk for 10,000 units and can be personalized for each user through a 3D-printable case.

The first sentence is rather odd and should be altered -- do the authors mean that hearing aids are the primary tool in non-medical rehabilitation for persons with hearing loss? The "audiological market" as is written is a very vague and wide term and should be avoided.

- We have edited this to include that hearing aids are the primary tool in non-medical rehabilitation for persons with hearing loss. (Line 12-13)

Consumer Technology Association for Hearing Aids. I don't understand why Hearing Aids is capitalized.

- We have fixed the capitalisation error for Hearing Aids. Thank you (Line 22)

p.2 Hearing aids are the "most frequently used" what in rehabilitation? Something missing. Same for "public policy" .. public policy what?

We have fixed the most frequently Hearing aids are the primary and the most frequent tool used to rehabilitate individuals, and improve their respective HRQoL. (Line 40-41)

We have reworded the sentence to - The reasons for the high cost include proprietary software and hardware, costs of distribution, and the refusal of coverage by public policy programs (like Medicare) and private insurance companies. (Line 48-50)

"..have been reported ... charateristics and .." is redundant and can be deleted.

-We have deleted them thank you. (Line 52-53)

p. 3 "supported" seems wrong in this context ... advocated would be more appropriate.

- We have fixed supported to advocated thank you. (Line 59-60)

line 70 .. both coupler and real-ear simulator measures were conducted through a Verifit Speechmap hearing aid analyzer. Or was another device used to measure the h/aid output from the KEMAR manikin? This sentence needs correction.

- No other device was used to measure the h/aid output from the KEMAR manikin, except the manikin itself. The original sentence reads as such -

  Second, we simulate the preferred gain for a range of ARHL profiles (SI Fig S1) in both a coupler (Verifit Speechmap), and a real-ear simulator (G.R.A.S KEMAR).

  We change the sentence to *Second, we simulate the preferred gain for a range of ARHL profiles (SI Fig S1) in a coupler using the Audioscan Verifit device, and through a real ear simulator using the G.R.A.S KEMAR manikin.*(Line 69-71)

p. 4 better if "requires few soldering points"

- We have fixed it to “requires few soldering points” (Line 91-92). Thank you.

p. 5 line 143 The THD statement is misleading. WHO only allows 8% at certain low frequencies, not as an overall figure. This statement needs correction.

- We have fixed the sentence to - The total harmonic distortion at 500, 1000, and 1600 Hz is very low at 1%, much less than the limits posed by WHO (8% at 500 Hz & 800 Hz, 2% at 1500 Hz), and CTA (5% at 500 Hz) (Line 143-145)

p. 7 "strength 65 dB SPL" is rather unscientific. Better if "an ISTS signal of 65 dB SPL was played".

- We have fixed this to “an ISTS signal of 65 dB SPL was played.” (Line 201)

p. 9 To avoid over-generalization besterr to state "which often do not exist in LMIC".

- We have fixed this to include which often do not exist in LMIC (Line 241)

Line 259 Why the "($140)" in the text? Seems not reason for this to appear.

- We have removed the $140 from the text. (Line 259)

p. 10 should be "mass manufacturer" [singular not plural].

- We have fixed this this to mass manufacturer. (Line 286)

p. 11 line 317 better if "a lower EIN for the device".

- We have fixed this to “a lower EIN for the device” (Line 317)

References : Not highlighted in text, as fixed in bibliography file.

Ref 3 should be "Beaver Dam"

Fixed.

Ref 4 should be Journal of Neuroscience - delete "The" and "the official..."

Fixed.

Ref 5 has no journal pages numbers

Fixed - added pg numbers 187-195.

Ref 15 National Academies of Sciences, Engineering, and Medicine should be capitalized.

Fixed.

Ref 20 World Health Organization is duplicated.

Fixed

Ref 36 "McPherson" is incorrectly spelled.

Fixed

Ref 37 No publication etc. details are provided.

Website - reference, url, access date is provided.

Ref 38 No publication etc. details are provided.

Website - reference, url, access date is provided

Figures 3 and 4b are almost impossible to read. I suggest creating multiple figures, one for male data, one for female data and one for hearing profile X, Y, Z data.

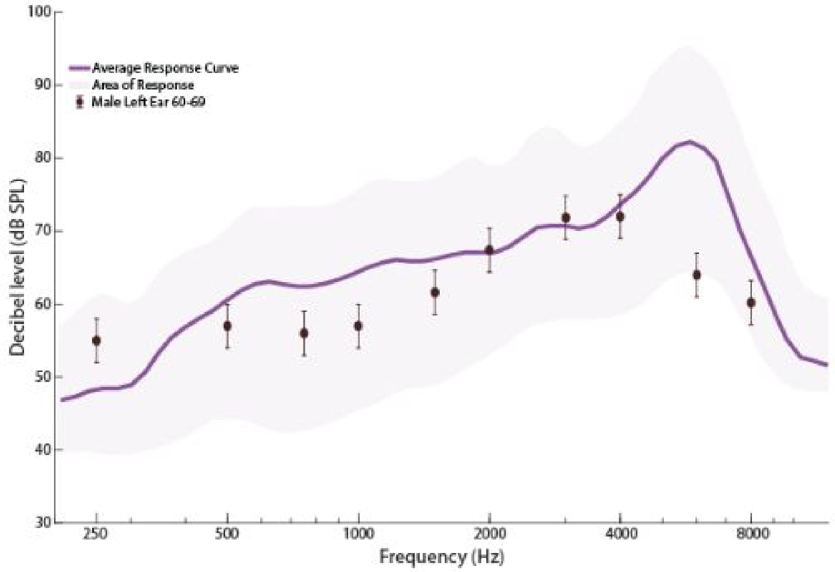

FIG. S14. Shows the ISTS 65 dB SPL Response (dB SPL) Curve with Male 60-69 Left Ear Targets (dB SPL).

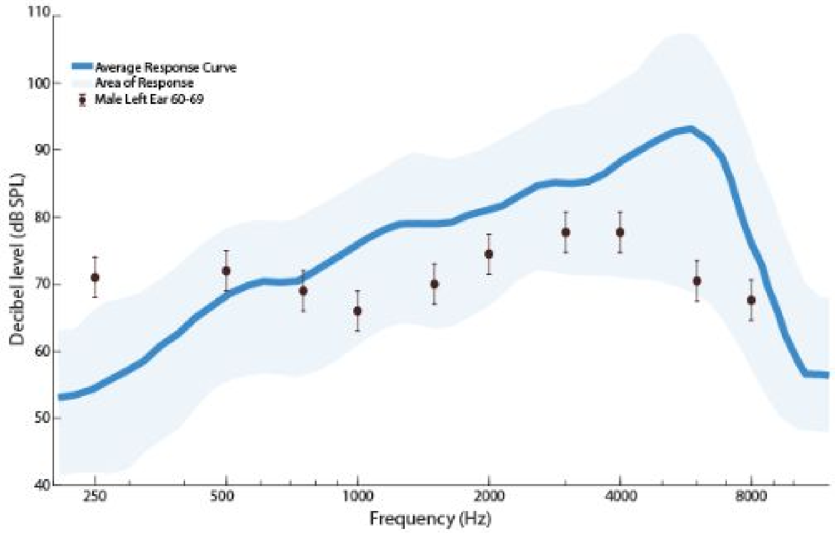

FIG. S15. Shows the ISTS 80 dB SPL Response (dB SPL) Curve with Male 60-69 Left Ear Targets (dB SPL).

Screenshot from SI showing individual graphs. (we have figures for each profile separately in the SI).

We have furthermore added to figure captions in Figure 3 and Figure 4 the following sentence - The reader is referred to SI Figs S9-S79 and Tables I-LX for individual profile targets and responses.

Table II legend Need to write "PPP" in full

- We have edited this to include Preferred Product Profile.

Throughout the m/s there were times when my PDF file showed missing spaces between letters, such as page 2, line 43 "... (e.g.,lack of...". Check the m/s thoroughly for such typos.

- We have fixed the space typo on line 43.
- We have fixed the space on between Fig 1c and d on line 92.
- We have added a space between the end of the sentence on Line 106 and the start of the next sentence
- We have added space between the end of the sentence on Line 115 and the start of the next sentence.
- We have added a space between the end of the sentence on Line 125 and the start of the next sentence.

Reviewer #2: - Comments in paper are given in Green.

Reposition the METHODS section, which should be after the introduction;

- We have repositioned methods after introduction.

The video mentioned in line 98 (Video 4) should show the impact of LoCHAid from different perspectives;

- Please refer to SI Figure 5a. We had already shown a still from the video.

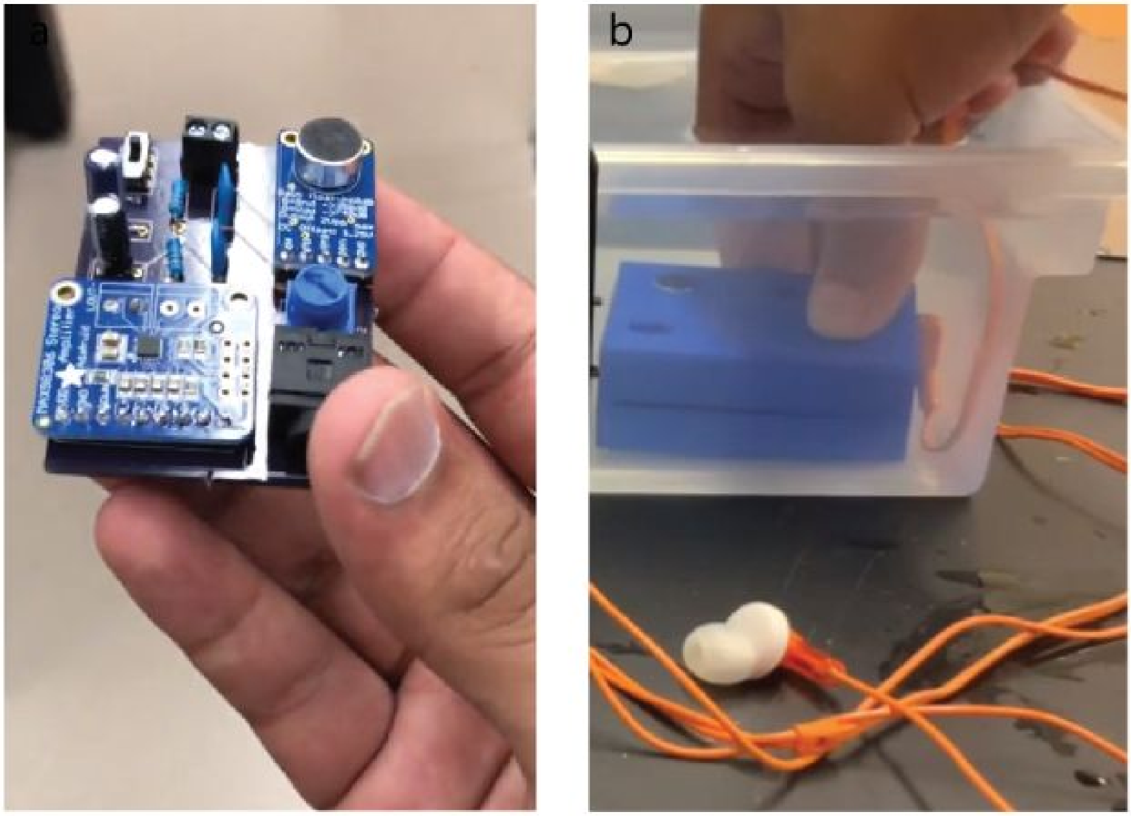

FIG. S5. **a** Still from SI Movie 4 detailing drop test of the device. **b** Still from SI Movie 3 detailing water test of the device.

Line 116: I suggest a sub-topic on the cost of the device;

- We have added a sub-topic - Cost of Manufacturing the LoCHAid. Line 116

Cost of Manufacturing the LoCHAid

When mass produced at 10,000 units with earphones, a coin-cell battery and a holder, the LoCHAid has a cost of \$0.98 (Table I) considering all components are bought from the listed suppliers in Table I description. Since the LoCHAid is constructed out of mass produced open source electronics, it does not require specialty made parts. As a result, repairs can be completed by a minimally skilled user with a soldering iron and solder. Moreover, the low cost nature allows LoCHAid to be be replaced very quickly and cheaply if parts are damaged, resulting in a relatively easy-to-use OTC device. Labor costs are not considered in the price point, as the device is intended to be manufactured by the individual (see Discussion below). A personalisable (and potentially fashionable) custom case can be readily 3D-printed using other polymers than Nylon 12 (which is shown in Fig 1a) at potentially a slightly higher price point. However,other materials can be readily used for the case, including acrylic, cardboard, and foam. Given that most hearing aids and PSAPs cost around \$4700 and \$300 for a pair respectively, our device shows a reduction of cost by 99.98%.

- Furthermore we also had a table in the original manuscript showing the cost of individual components in the mass-production.

**Components**	**Mass Production Cost**	
Earphones (**i**)	$0.04	
Audio Jack (**ii**)	$0.03	
2 x 1000 pF Capacitor (**iii**)	$0.02	
2 x 1 uF Capacitors (**iv**)	$0.02	
1 x 15 uF Capacitor (**v**)	$0.01	
5 kΩ Trim Pot Potentiometer (**vi**)	$0.06	
6 pronged - Slide Switch (**vii**)	$0.03	
Open Source Electret Microphone (**viii**)	$0.10	
Open Source Stereo Class D 3.7 Amplifier (**ix**)	$0.48	
Circuit Board (**x**)	$0.05	
3D Printed PLA Casing (**xi**)	$0.06	
2 x 6.8 kΩ Resistors (**xii**)	$0.02	
**Total Cost Without Batteries**	**$0.92**	**Total Cost With Batteries**
2 AA Alkaline Batteries and Holder (**xiii**)	$0.13	$1.05
3 V Coin Cell Battery and Holder (**xiv**)	$0.06	**$0.98**

TABLE I. **Component Costs of the LoCHAid.** The table lists the costs for acquiring individual components in bulk of 10000 pieces. The LoCHAid is assumed has been created from the following: (**i**) a set of earphones (ModelGF-923, In-Ear, 3.5mm Connector, from Boluo Golden Fortune Electronic Manufacture Factory, wwww.alibaba.com, P/N 60249739970), (**ii**) a audio jack (1/4" 3.5mm PCB Mount Female Socket 5 pin, from Yueqing Daier Electron Co. LTD, from www.alibaba.com, M/N EJ-214M); (**iii**) 2 1000pF capacitors (SMD/SMT 1000 pF 50V Multilayer Ceramic Capacitor, from Part Rescue Technology, from www.alibaba.com, M/N VJ0603Y102KXACW1BC);

Line 289: About the smaller prototype, I suggest a figure that shows the final device.

- We have added a figure to SI - Figure S8 that shows front and back views of the small device.

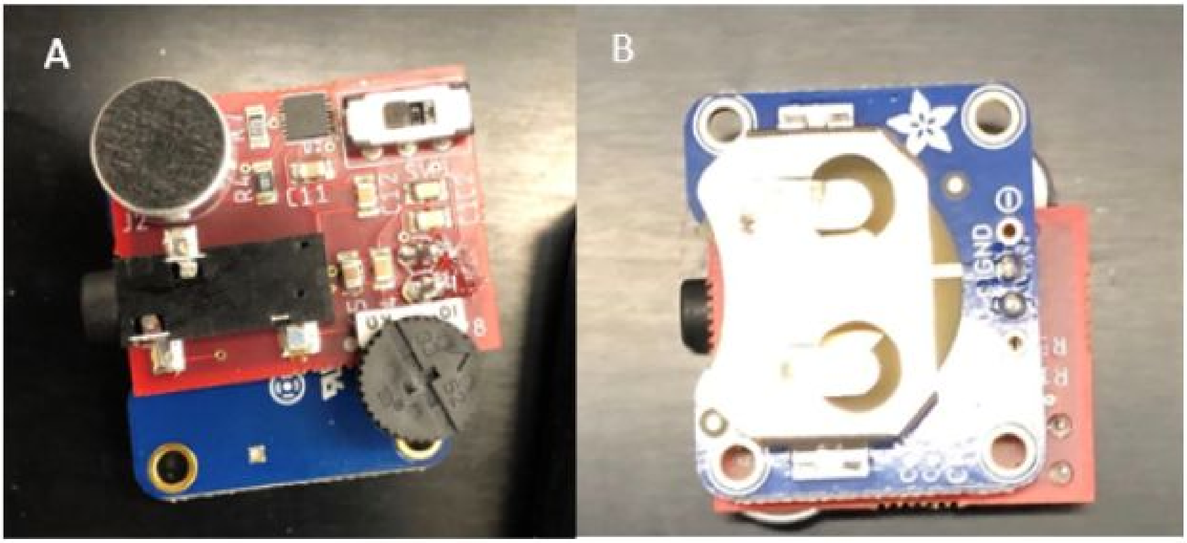

FIG. S8. A. Shows the front side view of the smaller version (1.05 x 0.81 in) of the LoCHAid. B. Shows the back side of the smaller version of the LoCHAid. It was assembled by third party fabrication house Macrofab (www.macrofab.com)
