# Supplementary material for: LoCHAid: An ultra-low-cost hearing aid for age-related hearing loss
Source: PLoS One. 2020 Sep 23;15(9):e0238922. doi: 10.1371/journal.pone.0238922 (PMC7510997; doi:10.1371/journal.pone.0238922)
Supplement: Attachment — Submitted filename: Round 2 Reviewer Comments.pdf [file pone.0238922.s007.pdf]

Reviewer #1: The revised m/s meets almost all my initial review comments and now has improved readability and focus. The m/s covers an area that is under-researched and I commend the research team for their exploratory work.

Bold: Reviewer 1 Comments

In Blue: Author Response

My remaining comments concern minor points. Attention to these would further improve the final m/s:

page 4/16 better if just "... with the manikin inside.";
-Thank you, we have fixed the sentence to "...with the manikin inside." (Previously it was "...with the manikin being inside.")

inconsistent use of units in the paper - here and in S4 Video legend feet are used rather than metric units. Metric units should be used at all times throughout the paper.
- Thank you - we have fixed units from imperial to metric units. (2.64 by 2.24 inches have been written as 6.70 mm by 5.70 mm). S4 video legend has been changed (5 feet has been changed to 1.5 meters)

page 5/16 mentions an estimated device lifespan of only 1.5 years. This issue should also be very briefly considered in the discussion.
- We have added the following paragraph to the discussion section - Potential Shortcomings of the LoCHAid

We have conservatively estimated the device lifetime to be 1.5 years, which is considerably less than an ideal lifetime of 5 years. However, with the low cost of manufacture, we anticipate that even with replacement costs, the LoCHAid will still be affordable. We have not conducted failure tests or lifetime tests of the LoCHAid to a considerable extent in this paper. A part of our future work will be seeing the effectiveness and usability in different environments and locations throughout the world.

Table 2, page 7/16. change "HZ" to "Hz" as in all other instances in the table.
- Thank you, we have changed HZ to Hz.

page 10/16 makes the claim that the low cost device can be used in "screening for potential hearing loss". This is a puzzling statement because nowhere in the m/s is a device screening function mentioned. I think this goes well beyond the scope of the present research paper and should be deleted.
- Thank you we have removed the sentence "screening for potential hearing loss." We have now written "Like other free and open source hardware (FOSH), our open-source device empowers local communities to be involved in every step of use of the device, from its creation and construction, to repair and disbursement of devices to those in need. "

Some references still have problems: #4 should be just "The Journal of Neuroscience"; #15 and #19 just "JAMA"; #23, #24, #26, #27 and #34 are all incorrectly titled and need revision. #43 there should be no space before "?"
-Thank you - we have fixed references 4, 15, 19, 23-26, 27, 34, and 43.
